# Supplementary material for: The ZIKV NS5 Protein Aberrantly Alters the Tubulin Cytoskeleton, Induces the Accumulation of Autophagic p62 and Affects IFN Production: HDAC6 Has Emerged as an Anti-NS5/ZIKV Factor
Source: Cells. 2024 Mar 29;13(7):598. doi: 10.3390/cells13070598 (PMC11011779; doi:10.3390/cells13070598)

Figure S1A. Western-blot replicates associated with Figure 1A.

Replicate 1 as figure format

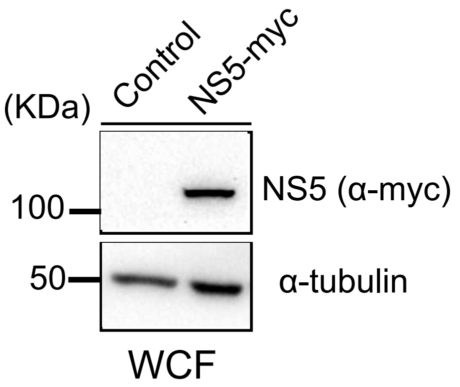

Replicate 2 as figure format

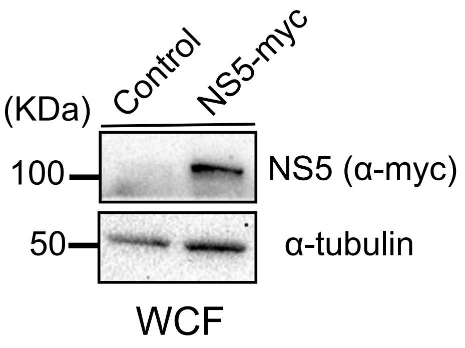

Figure S1A. *Replicate 1 myc Total Lysate complete gel*

Western-blot associated with Figure 1A
Pérez-Yanes, S., et al.

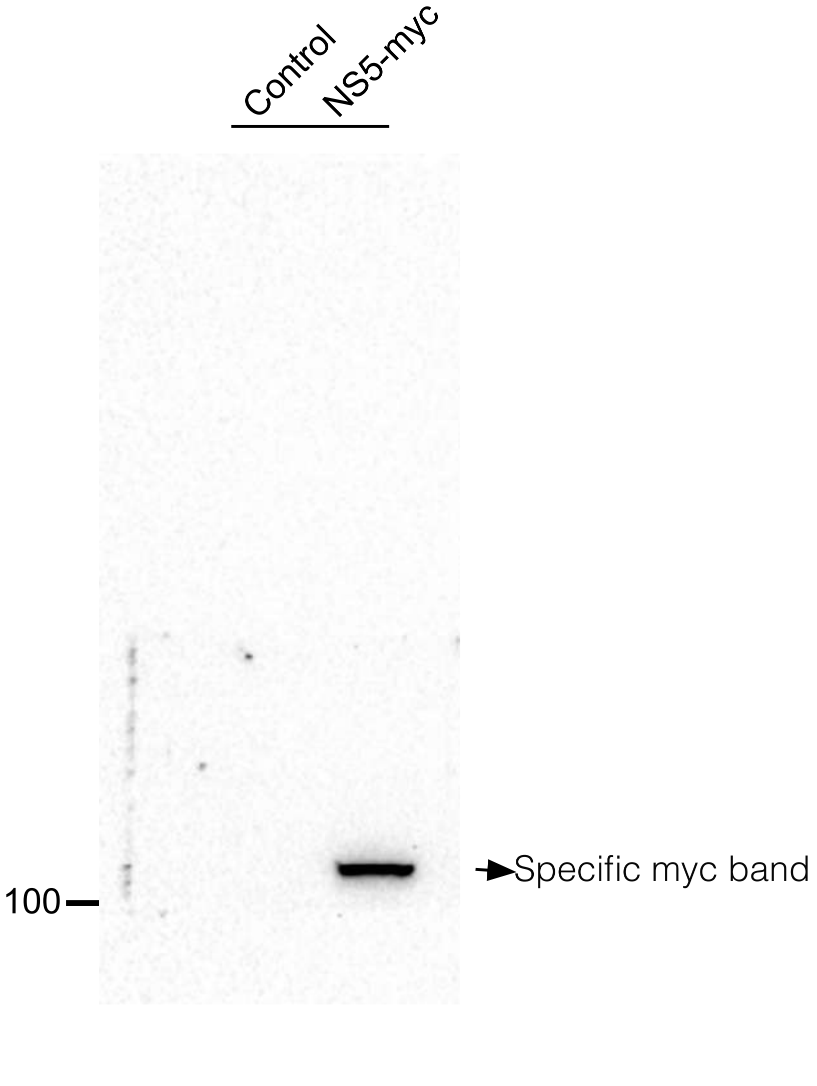

Figure S1A. *Replicate 1 Total α-Tubulin Total Lysate complete gel*

Western-blot associated with Figure 1A Pérez-Yanes, S., et al.

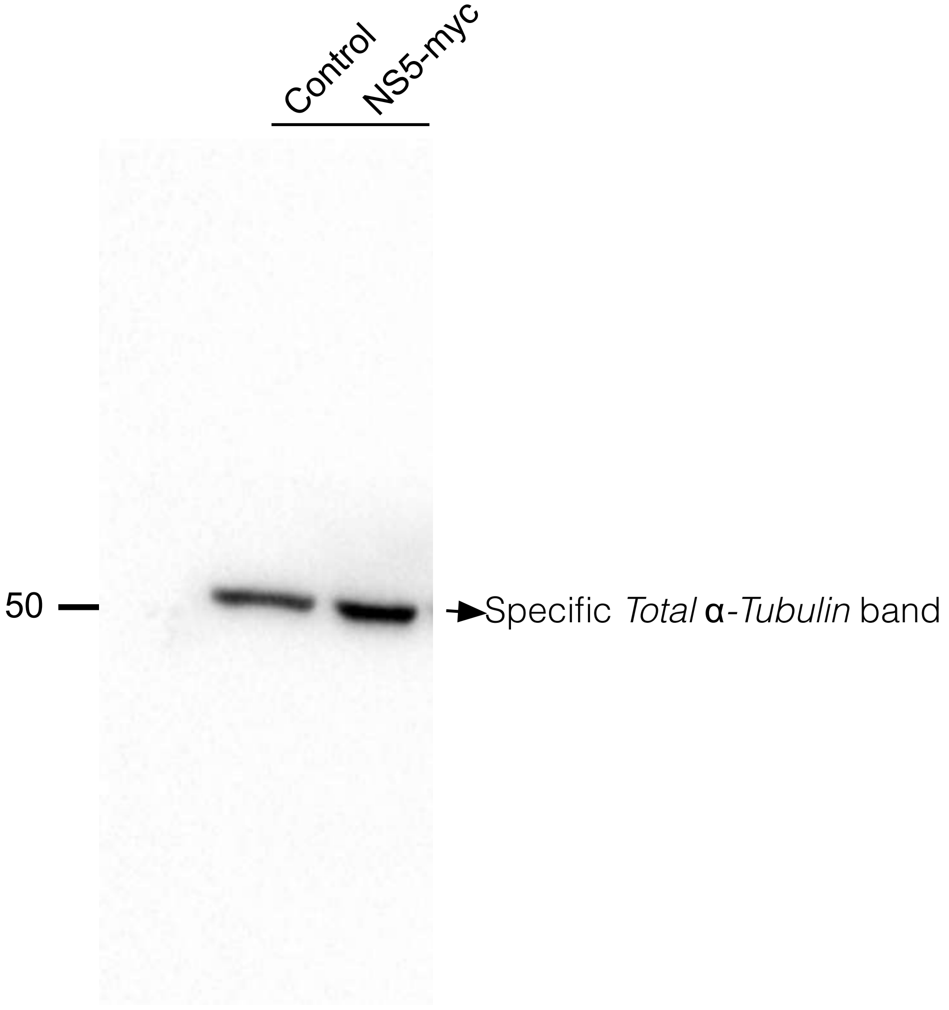

Figure S1A. *Replicate 2 myc Total Lysate complete gel*

Western-blot associated with Figure 1A
Pérez-Yanes, S., et al.

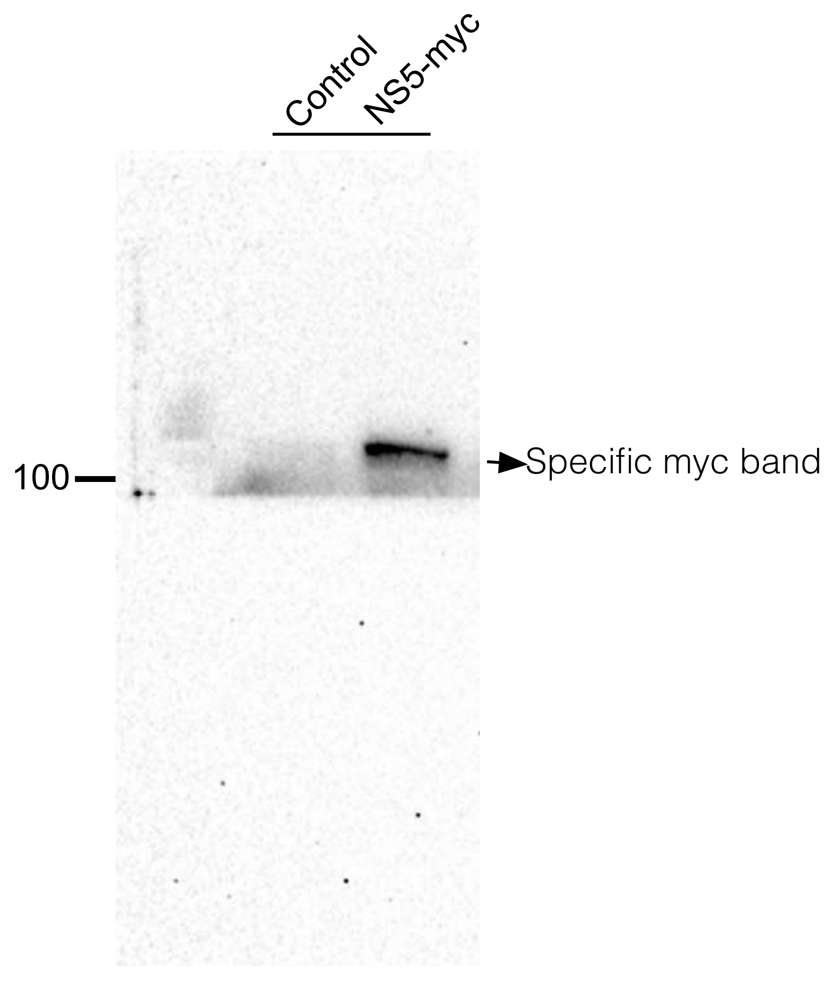

Figure S1A. *Replicate 2 Total* α*-Tubulin Total Lysate complete gel*

Western-blot associated with Figure 1A Pérez-Yanes, S., et al.

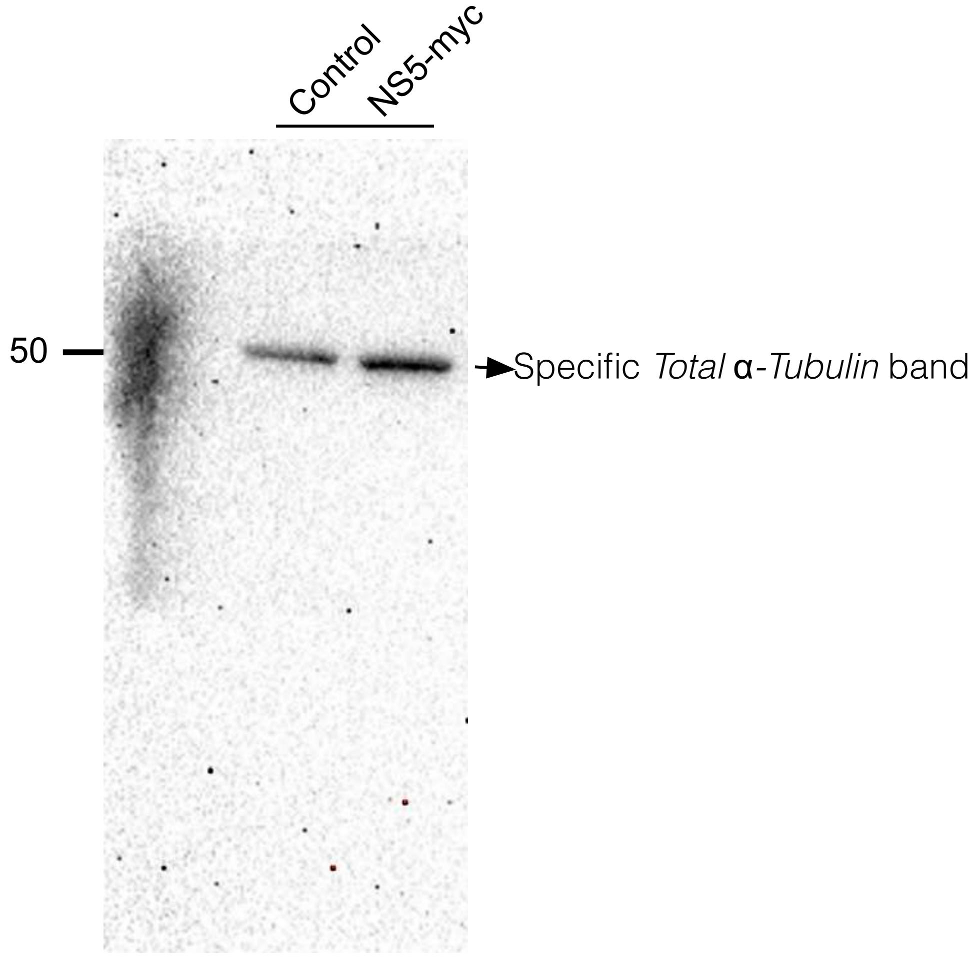

Figure S1B. Western-blot replicates associated with Figure 1B.

Replicate 1 as figure format

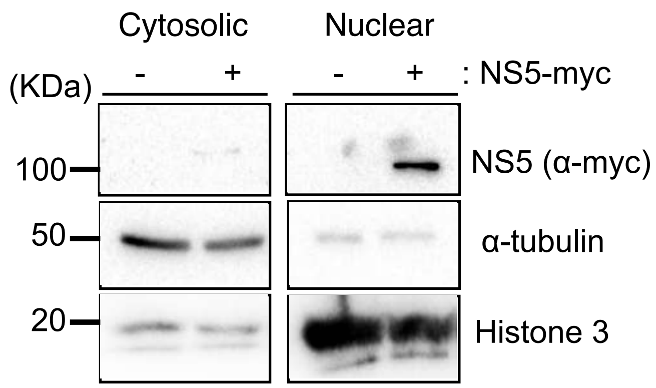

Replicate 2 as figure format

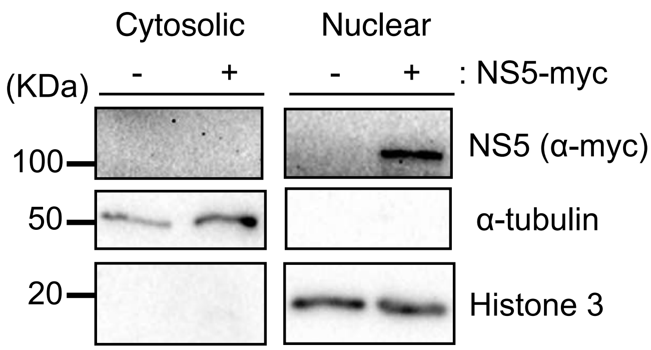

Figure S1B. *Replicate 1 Histone 3 Fraction complete gel*

Western-blot associated with Figure 1B
Pérez-Yanes, S., et al.

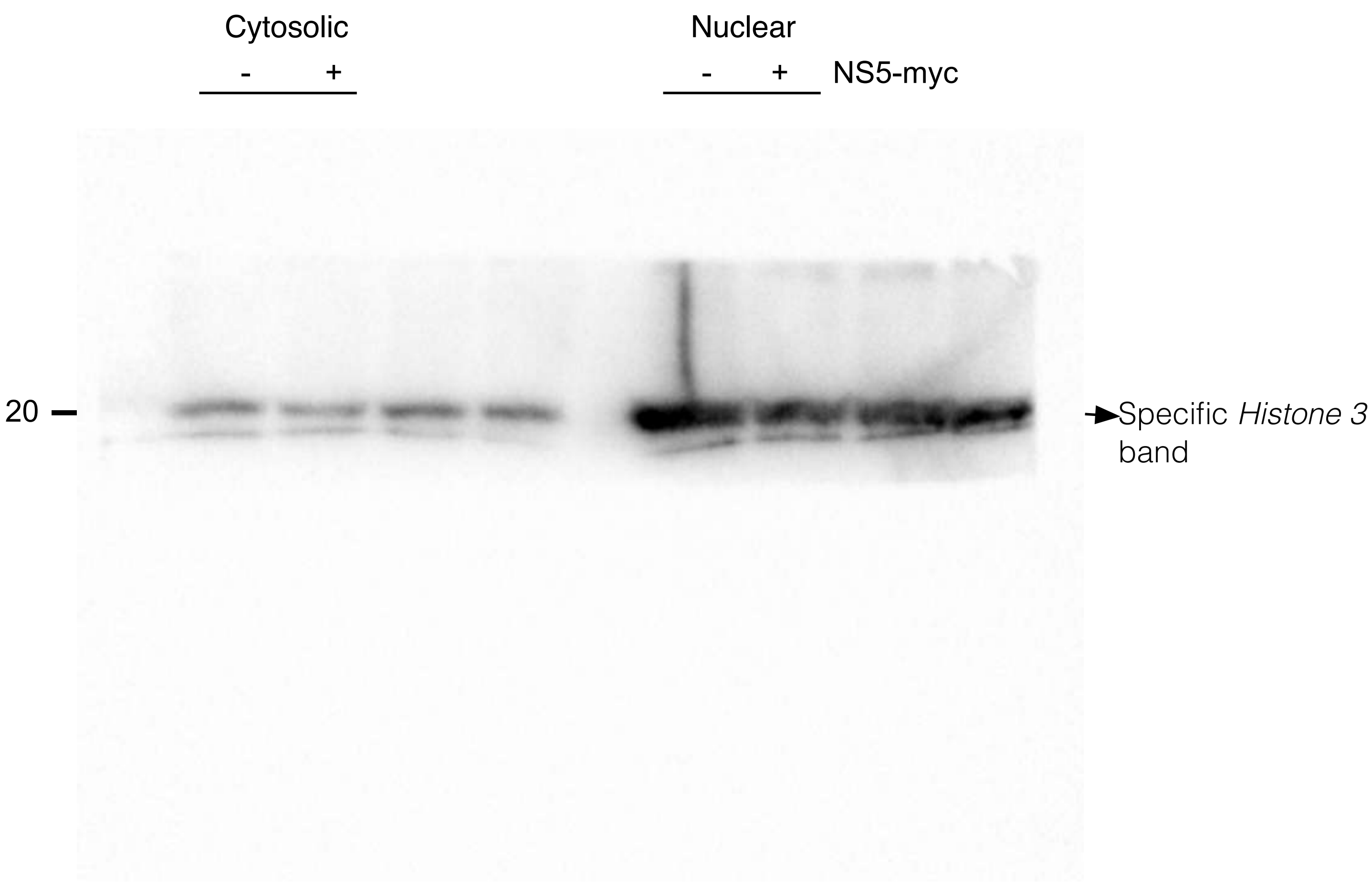

Figure S1B. *Replicate 1 myc Fraction complete gel*

Western-blot associated with Figure 1B
Pérez-Yanes, S., et al.

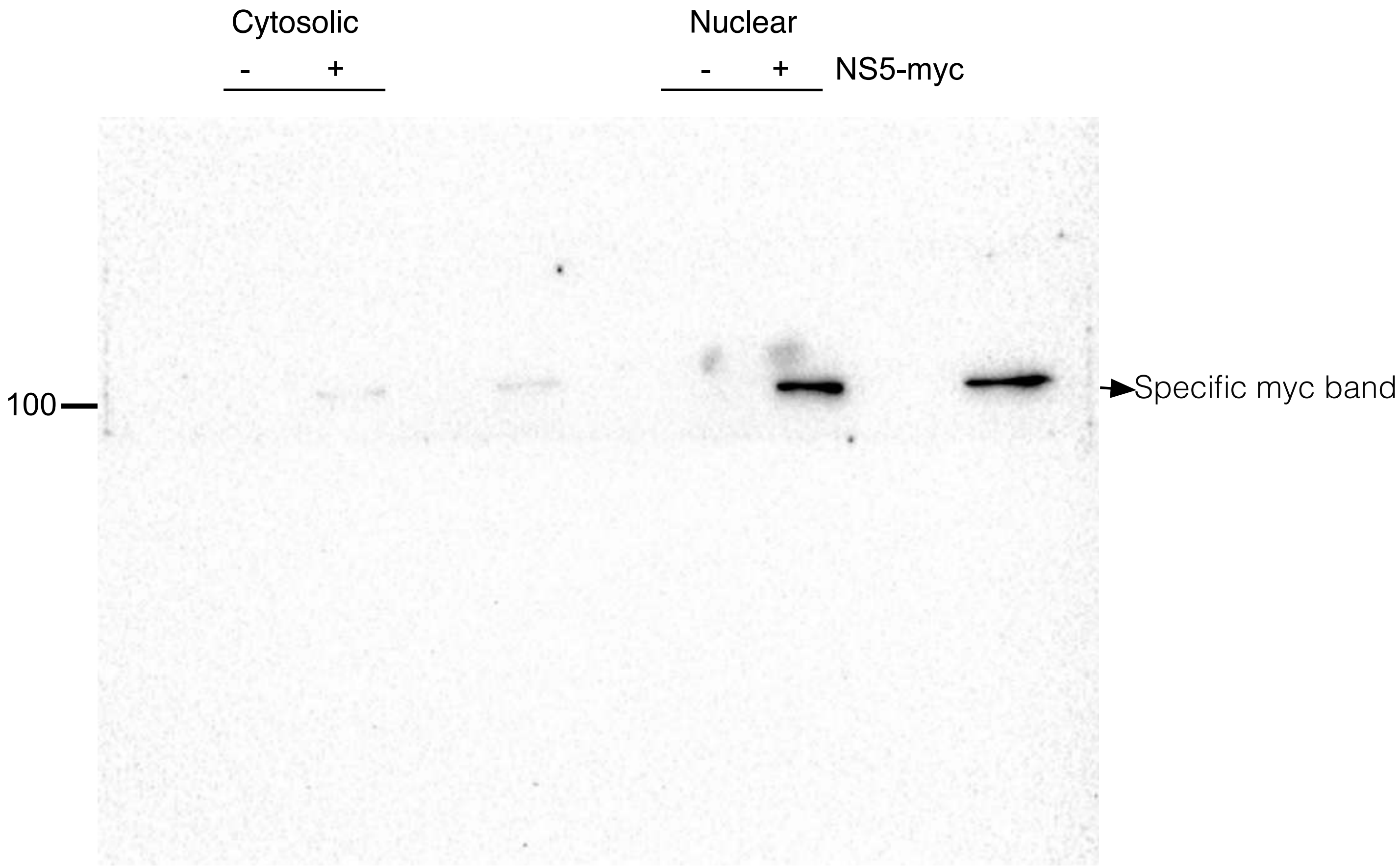

Figure S1B. *Replicate 1 Total* α*-Tubulin Fraction complete gel*

Western-blot associated with Figure 1B
Pérez-Yanes, S., et al.

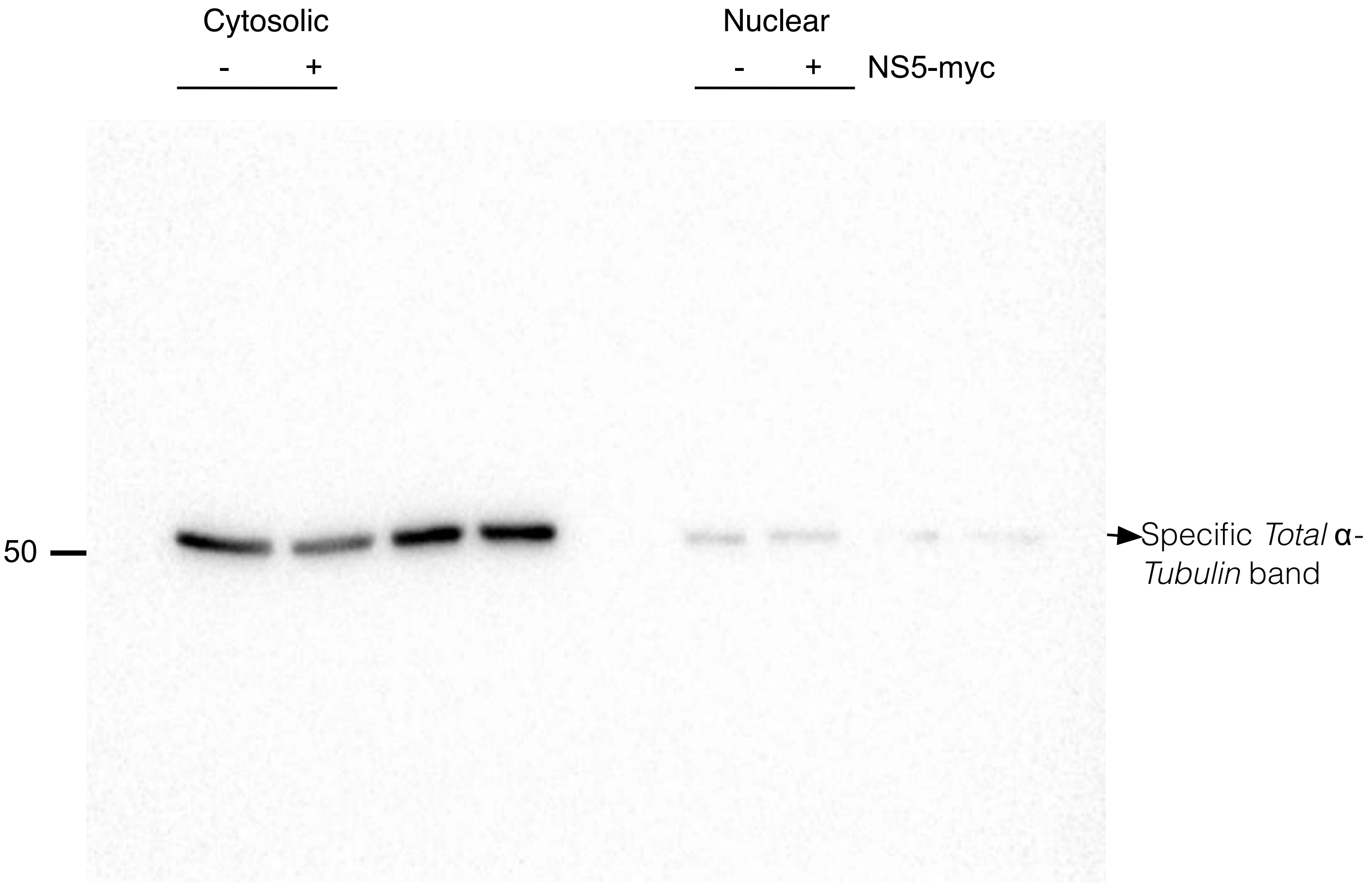

Figure S1B. *Replicate 2 Histone 3 Fraction complete gel*

Western-blot associated with Figure 1B
Pérez-Yanes, S., et al.

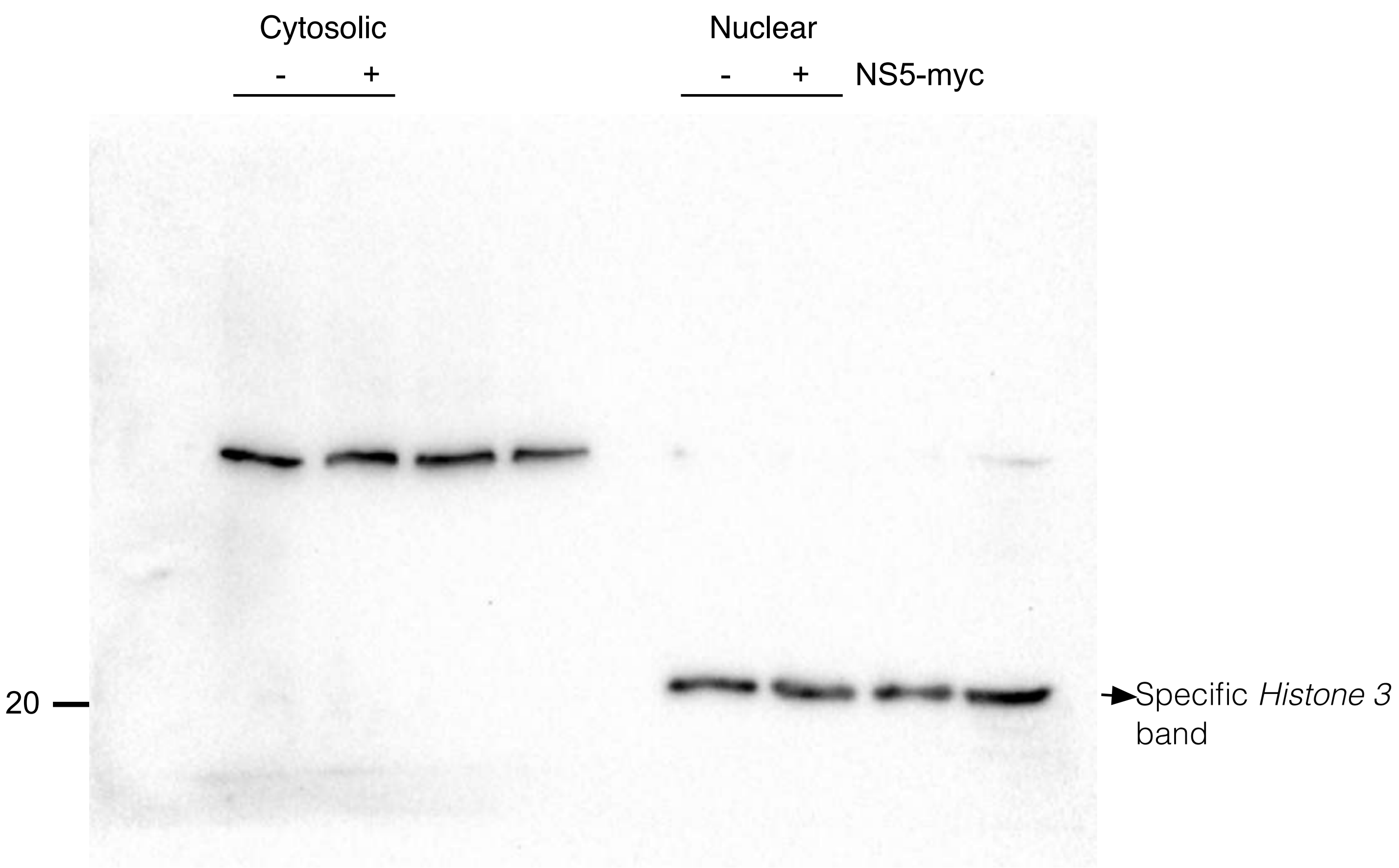

Figure S1B. *Replicate 2 myc Fraction complete gel*

Western-blot associated with Figure 1B
Pérez-Yanes, S., et al.

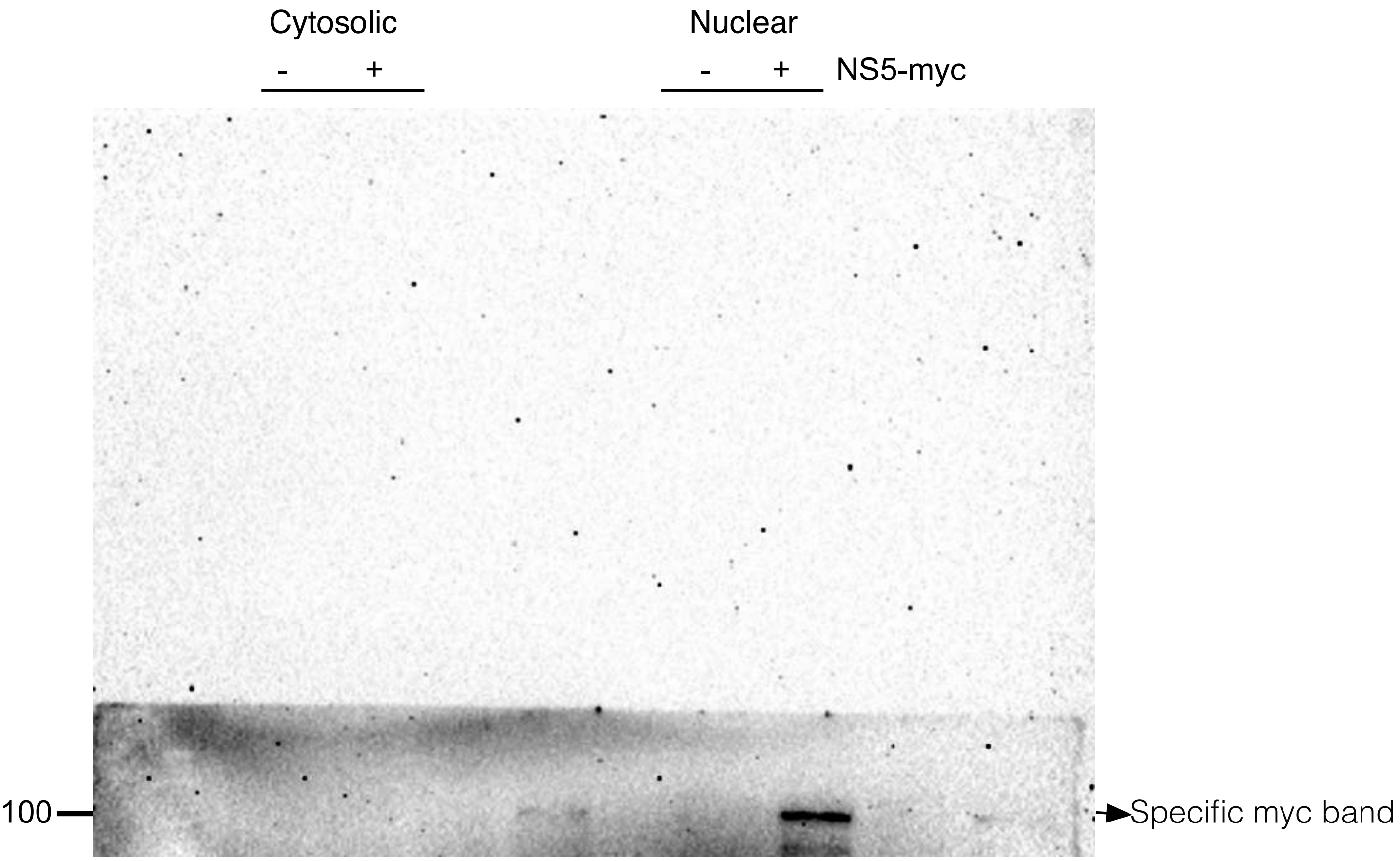

Figure S1B. *Replicate 2 Total α-Tubulin Fraction complete gel*

Western-blot associated with Figure 1B
Pérez-Yanes, S., et al.

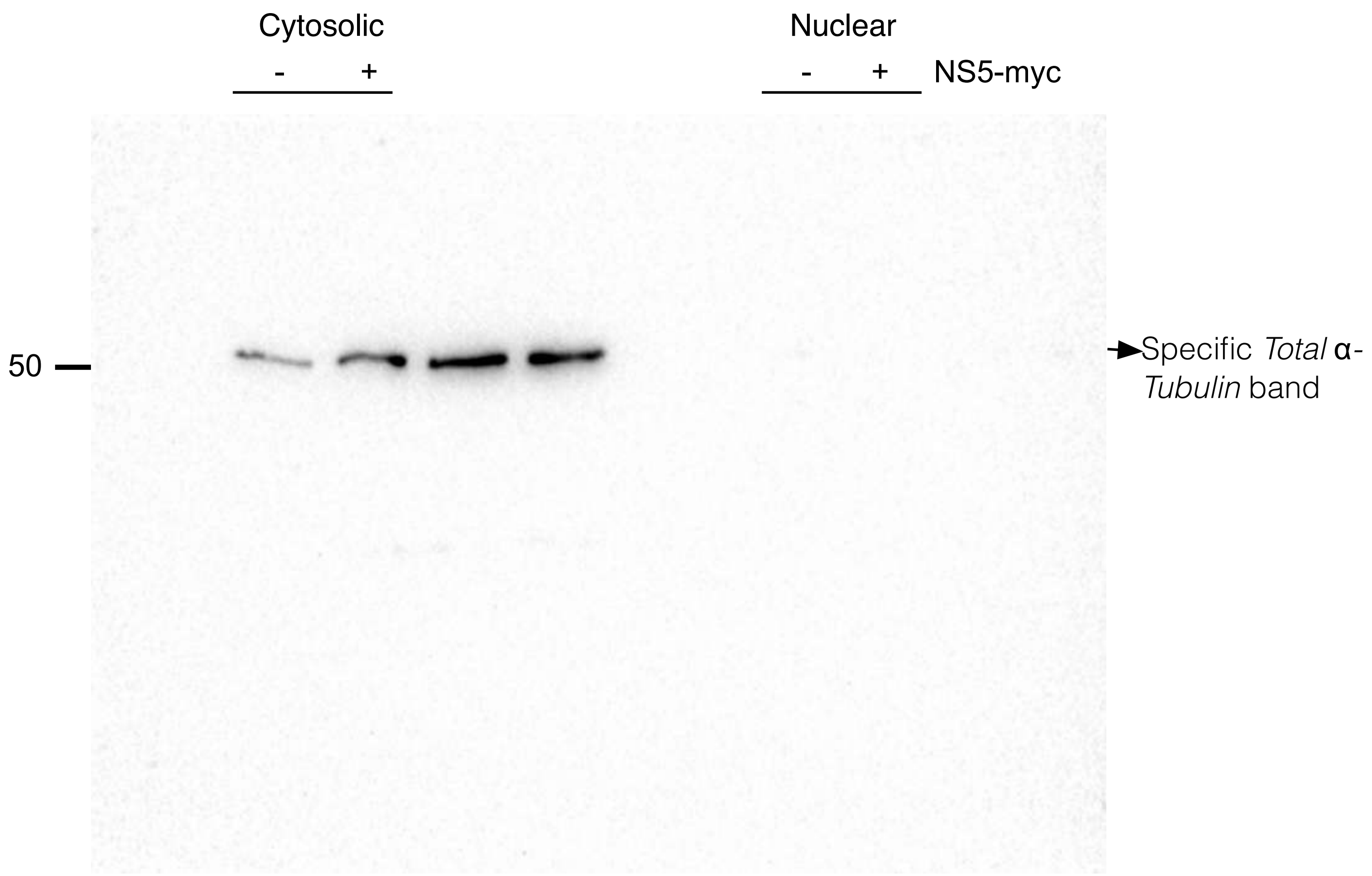

Figure S2C. Western-blot replicates associated with Figure 2C.

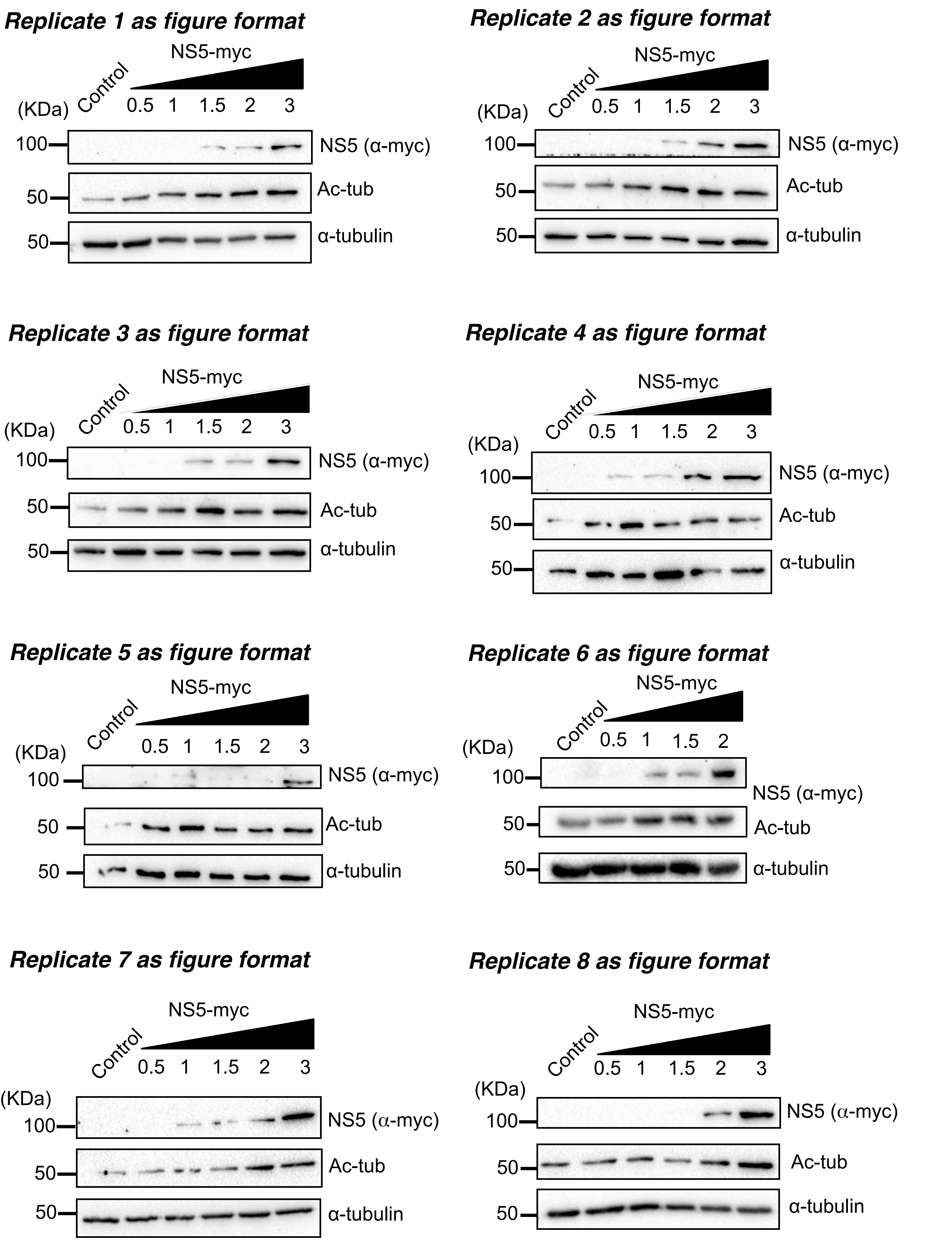

Figure S2C. Western-blot replicates associated with Figure 2C.

Replicate 9 as figure format

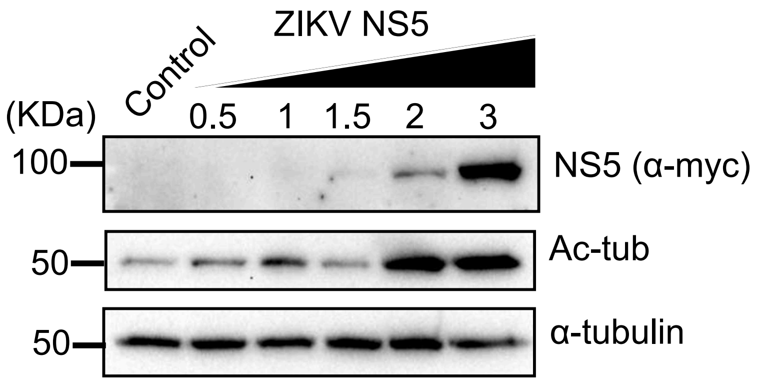

Figure S2C. *Replicate 1 myc complete gel*

Western-blot associated with Figure S2C
Pérez-Yanes, S., et al.

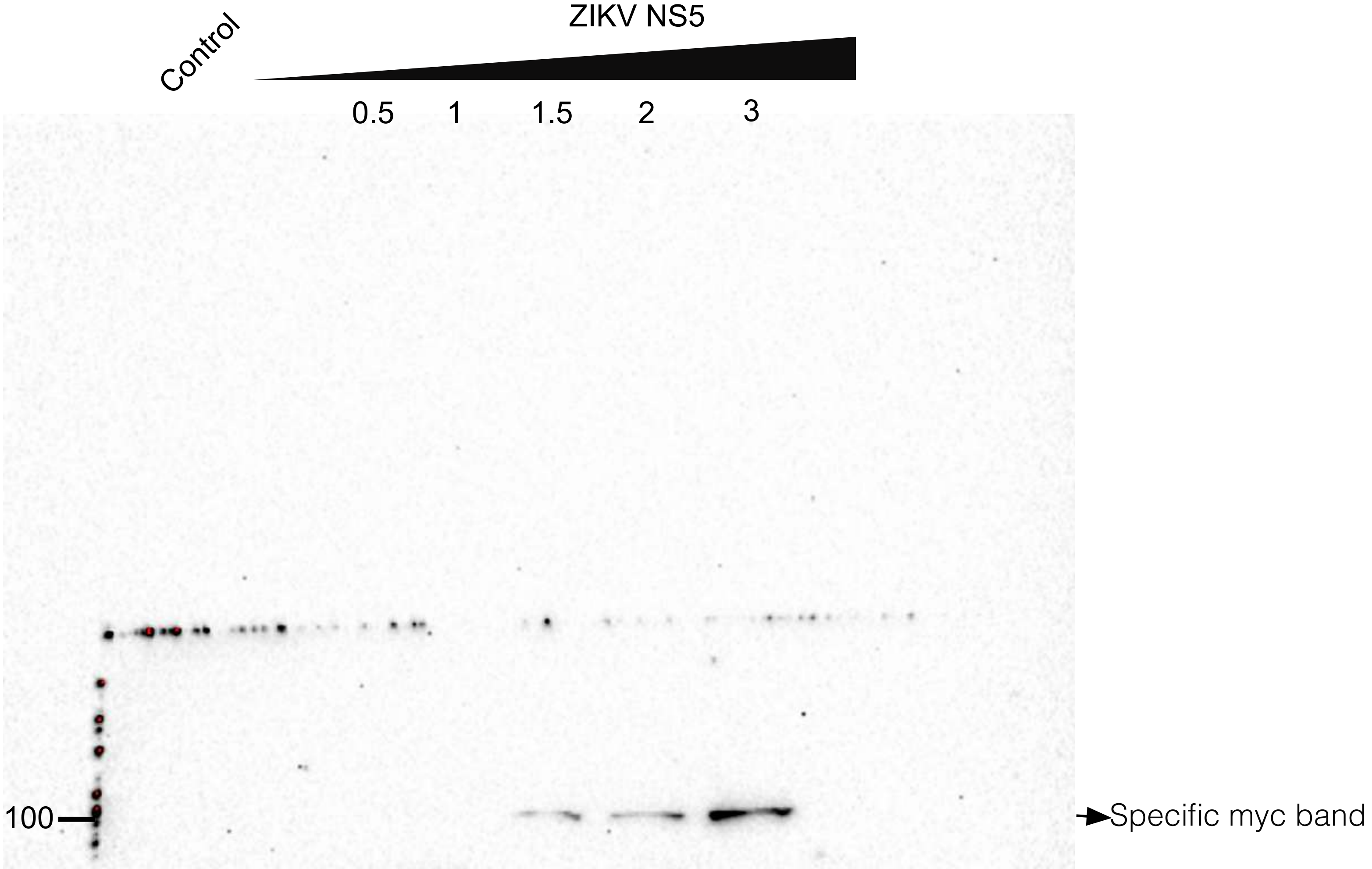

Figure S2C. *Replicate 1 Total* α*-Tubulin complete gel*

Western-blot associated with Figure 2C Pérez-Yanes, S., et al.

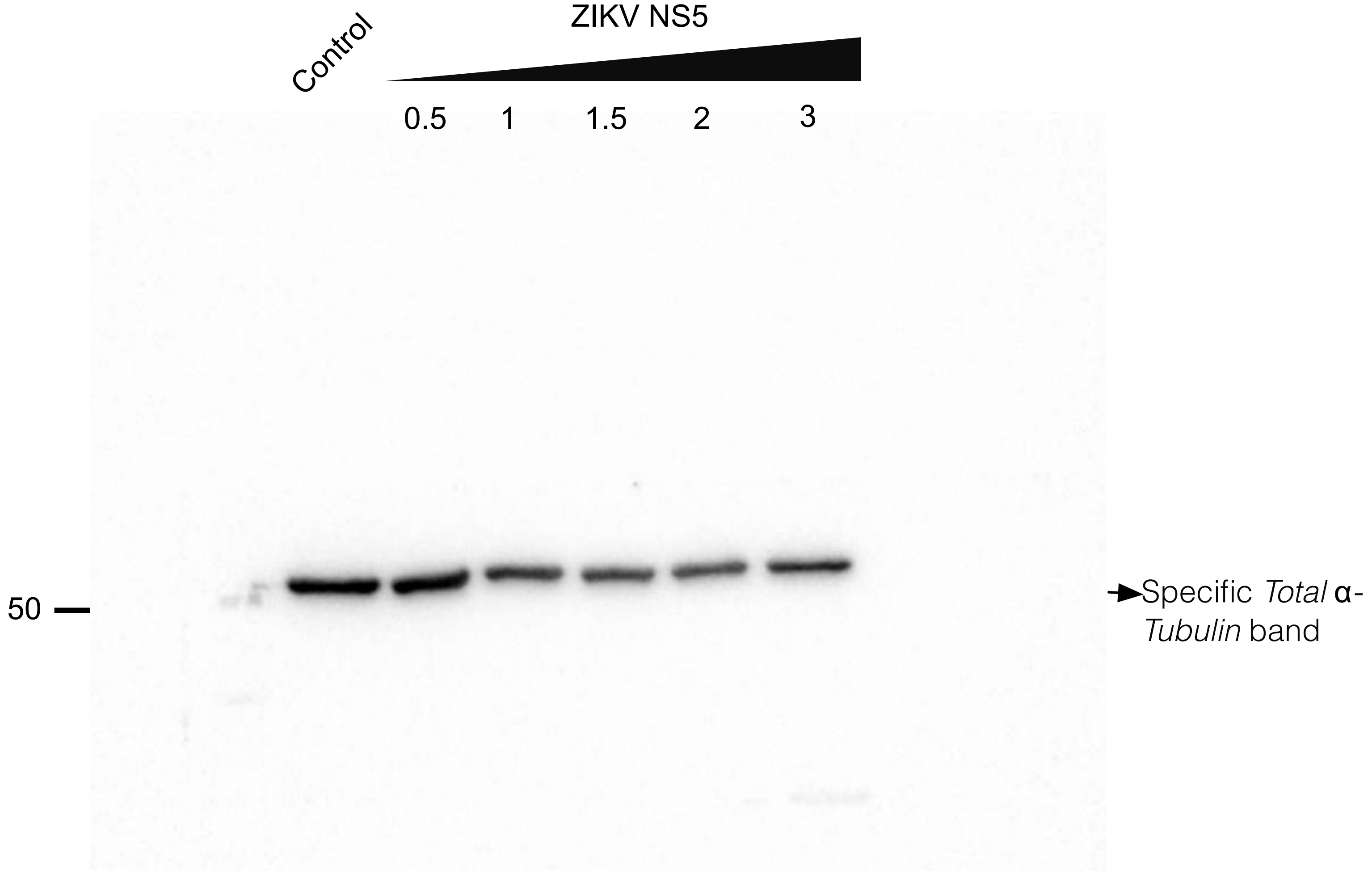

Figure S2C. *Replicate 1 Acetylated* α*-Tubulin complete gel*

Western-blot associated with Figure S2C Pérez-Yanes, S., et al.

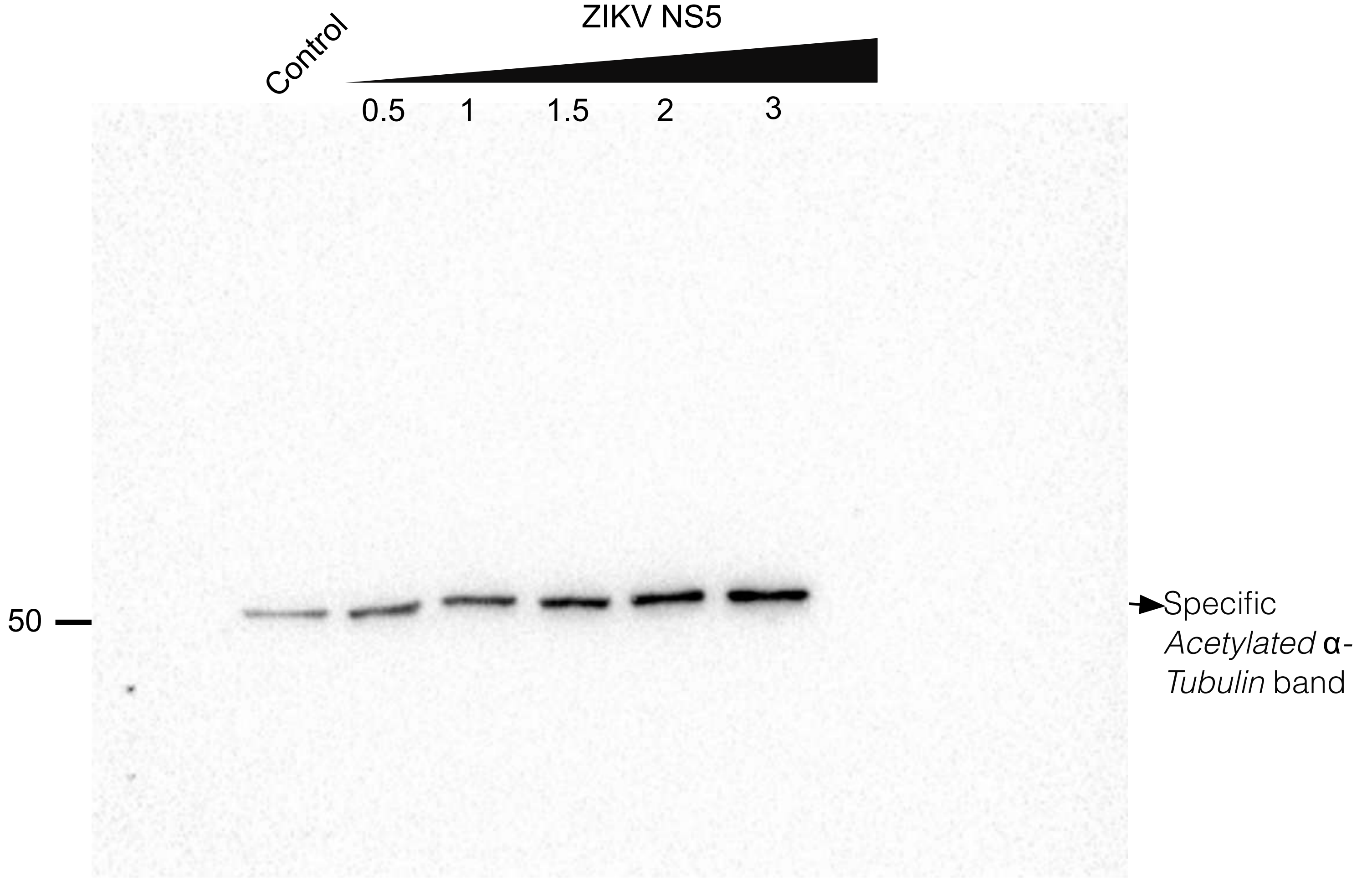

Figure S2C. *Replicate 2 myc complete gel*

Western-blot associated with Figure S2C
Pérez-Yanes, S., et al.

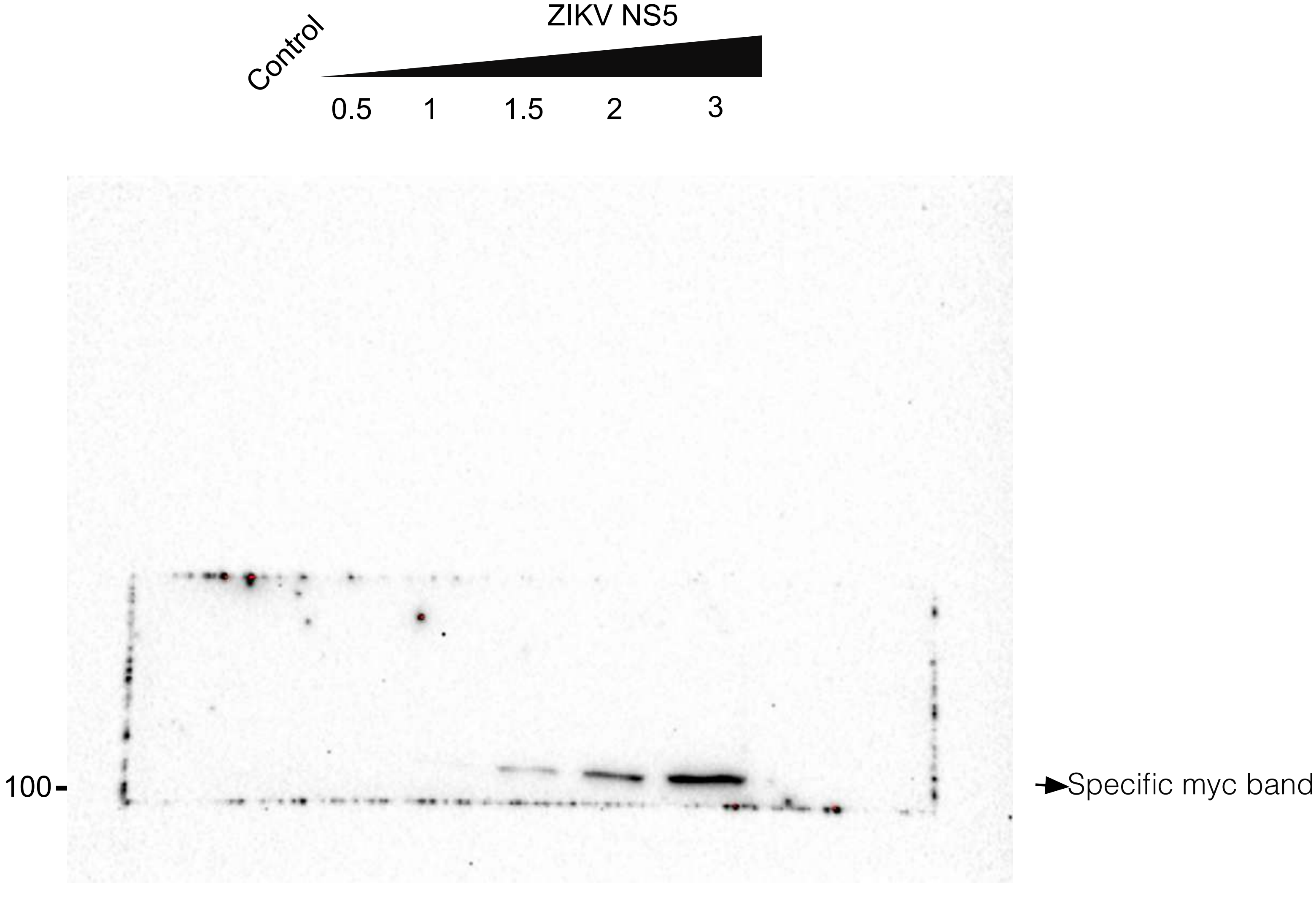

Figure S2C. *Replicate 2 Total* α*-Tubulin complete gel*

Western-blot associated with Figure 2C
Pérez-Yanes, S., et al.

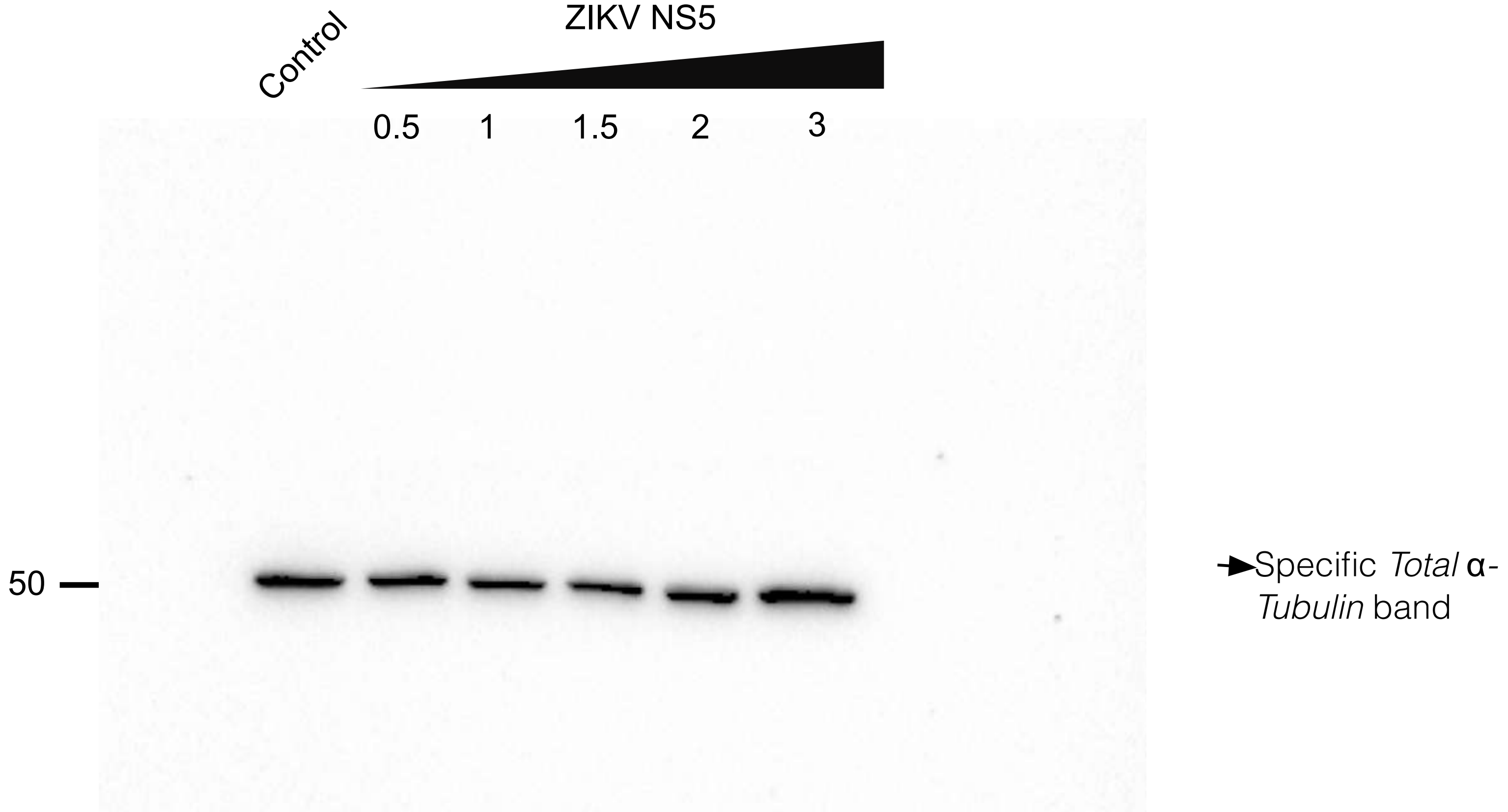

Figure S2C. *Replicate 2 Acetylated* α*-Tubulin complete gel*

Western-blot associated with Figure S2C
Pérez-Yanes, S., et al.

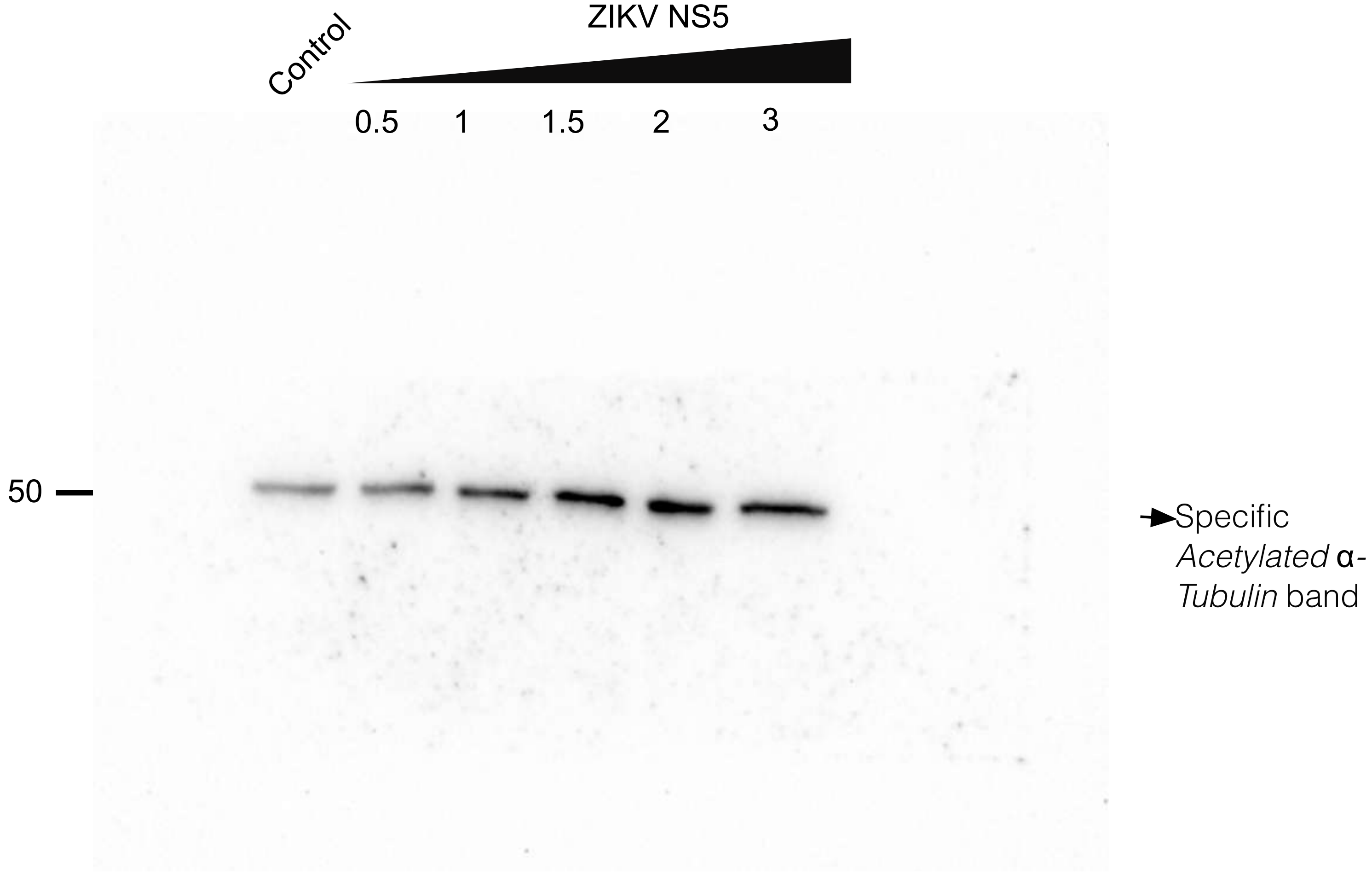

Figure S2C. *Replicate 3 myc complete gel*

Western-blot associated with Figure S2C
Pérez-Yanes, S., et al.

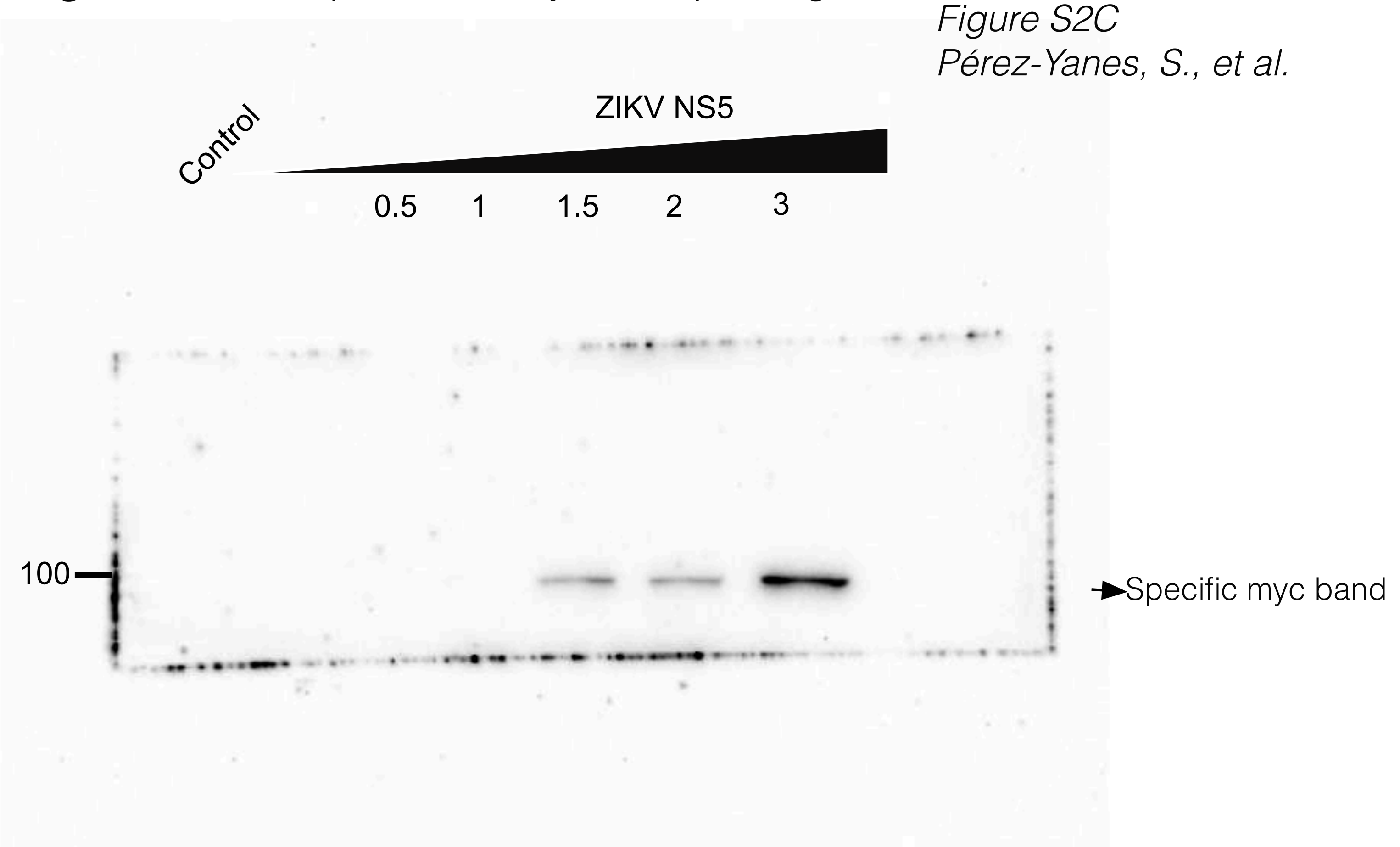

Figure S2C. *Replicate 3 Total* α-*Tubulin complete gel*

Western-blot associated with Figure 2C Pérez-Yanes, S., et al.

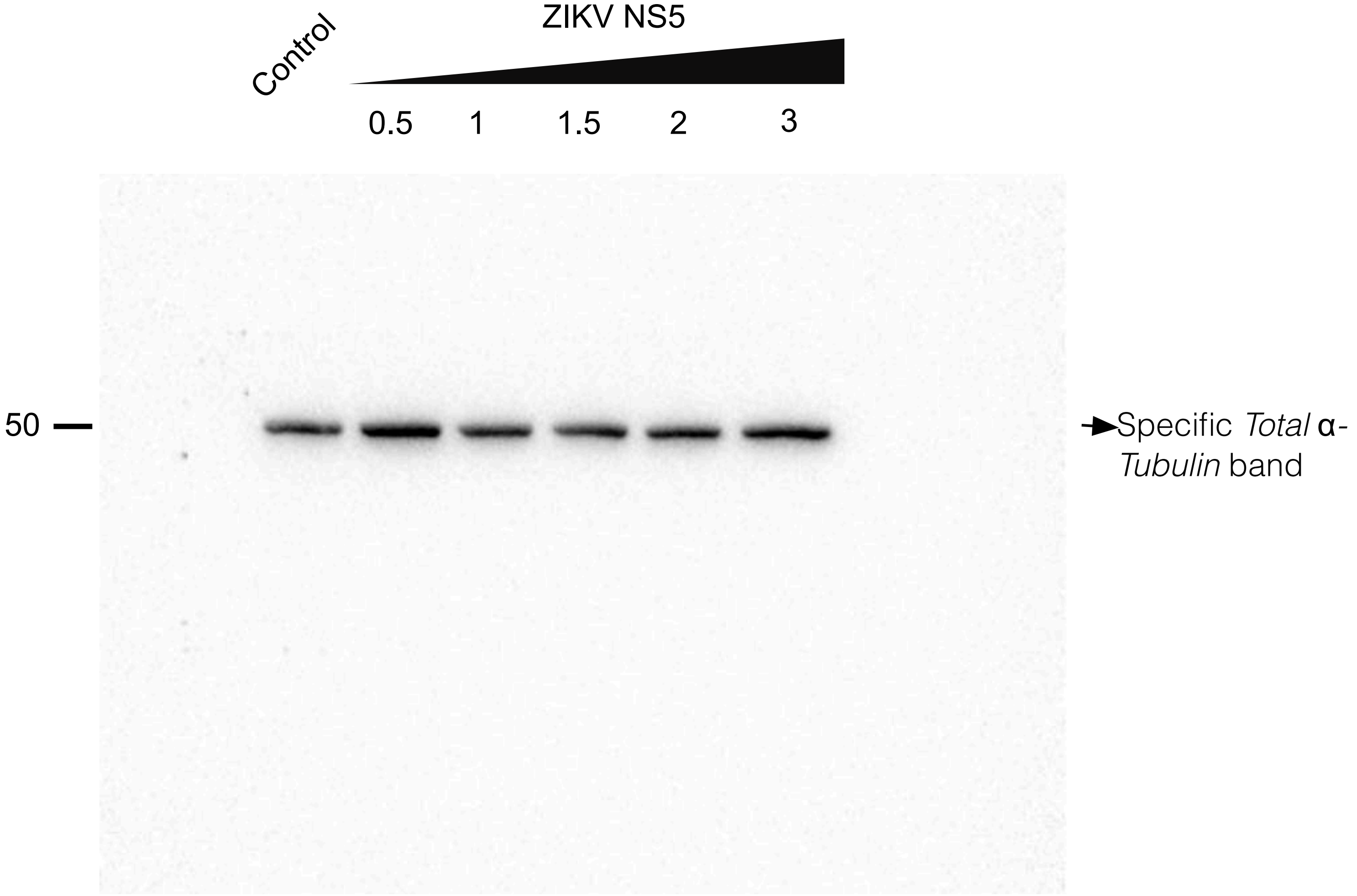

Figure S2C. *Replicate 3 Acetylated* α*-Tubulin complete gel*

Western-blot associated with Figure S2C Pérez-Yanes, S., et al.

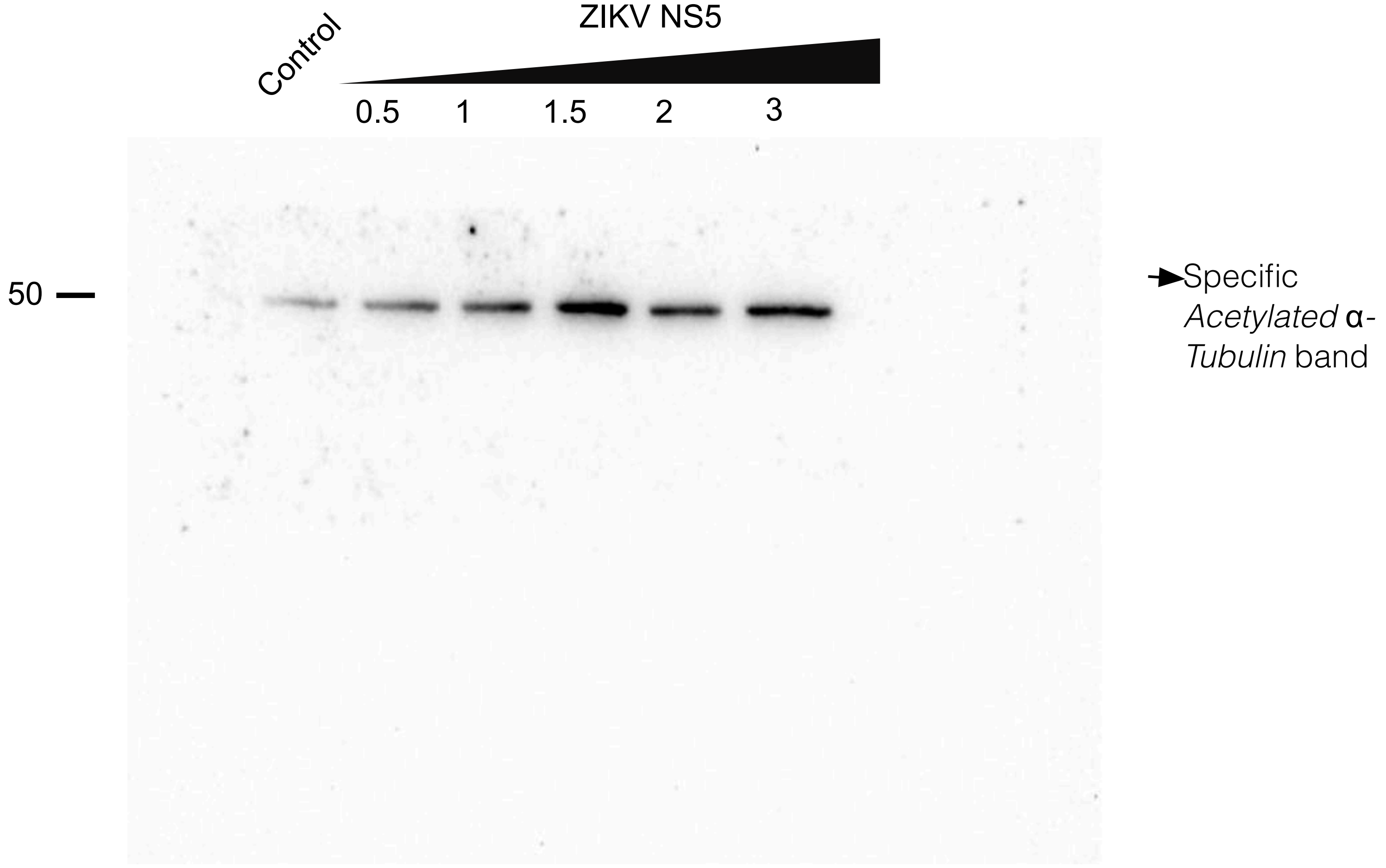

Figure S2C. *Replicate 4 myc complete gel*

Western-blot associated with Figure S2C
Pérez-Yanes, S., et al.

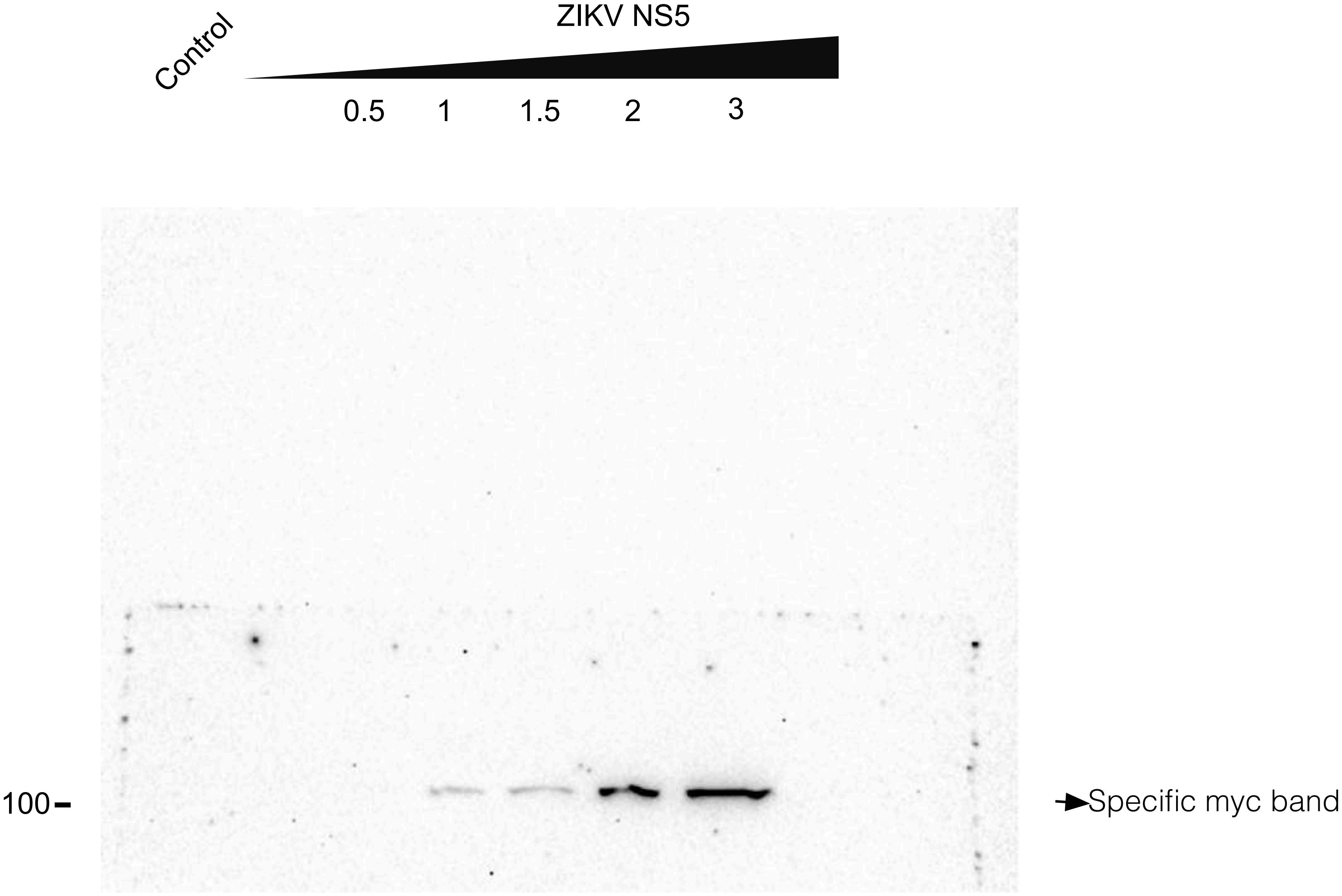

Figure S2C. *Replicate 4 Total* α*-Tubulin complete gel*

Western-blot associated with Figure 2C
Pérez-Yanes, S., et al.

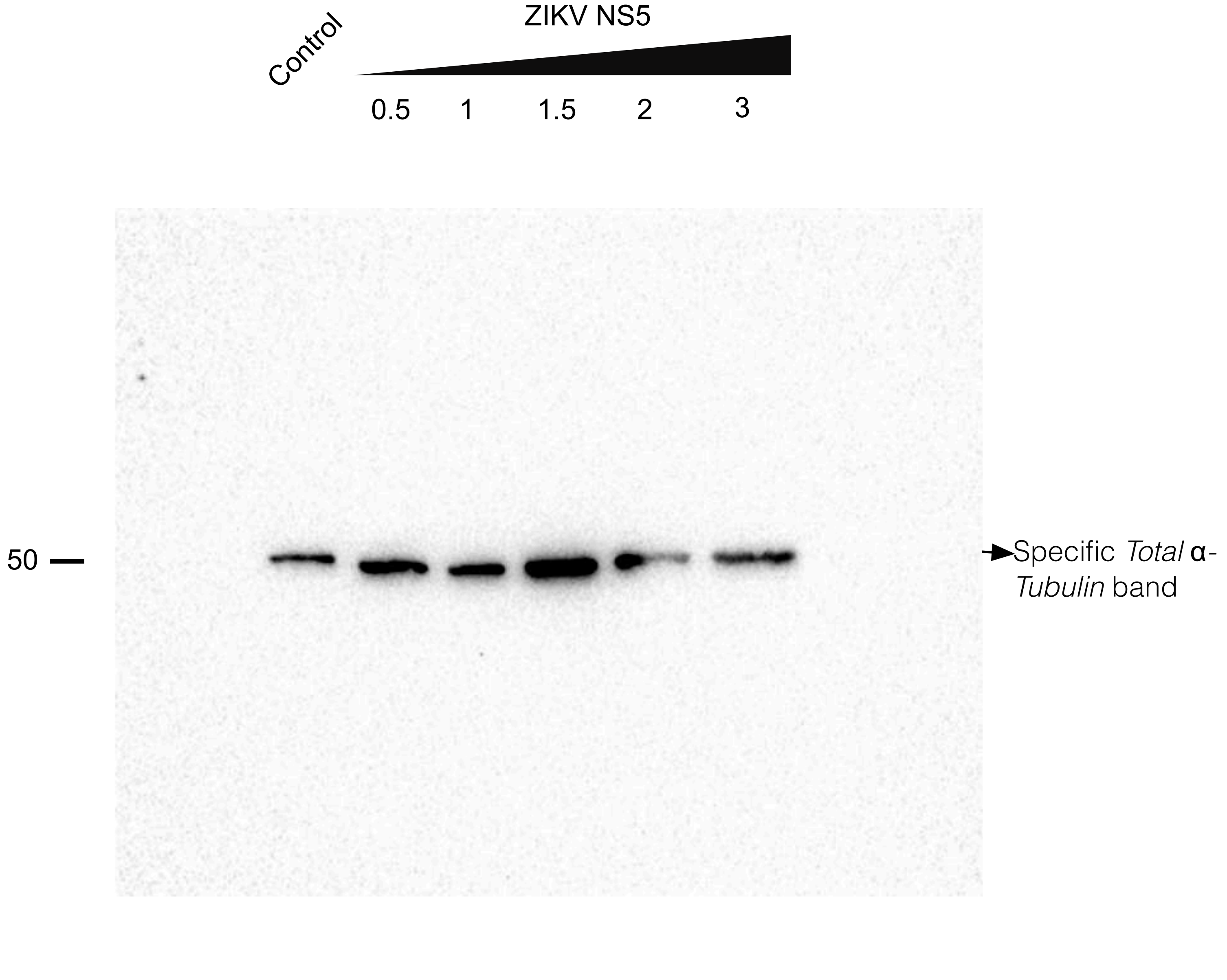

Figure S2C. *Replicate 4 Acetylated* α*-Tubulin complete gel*

Western-blot associated with Figure S2C
Pérez-Yanes, S., et al.

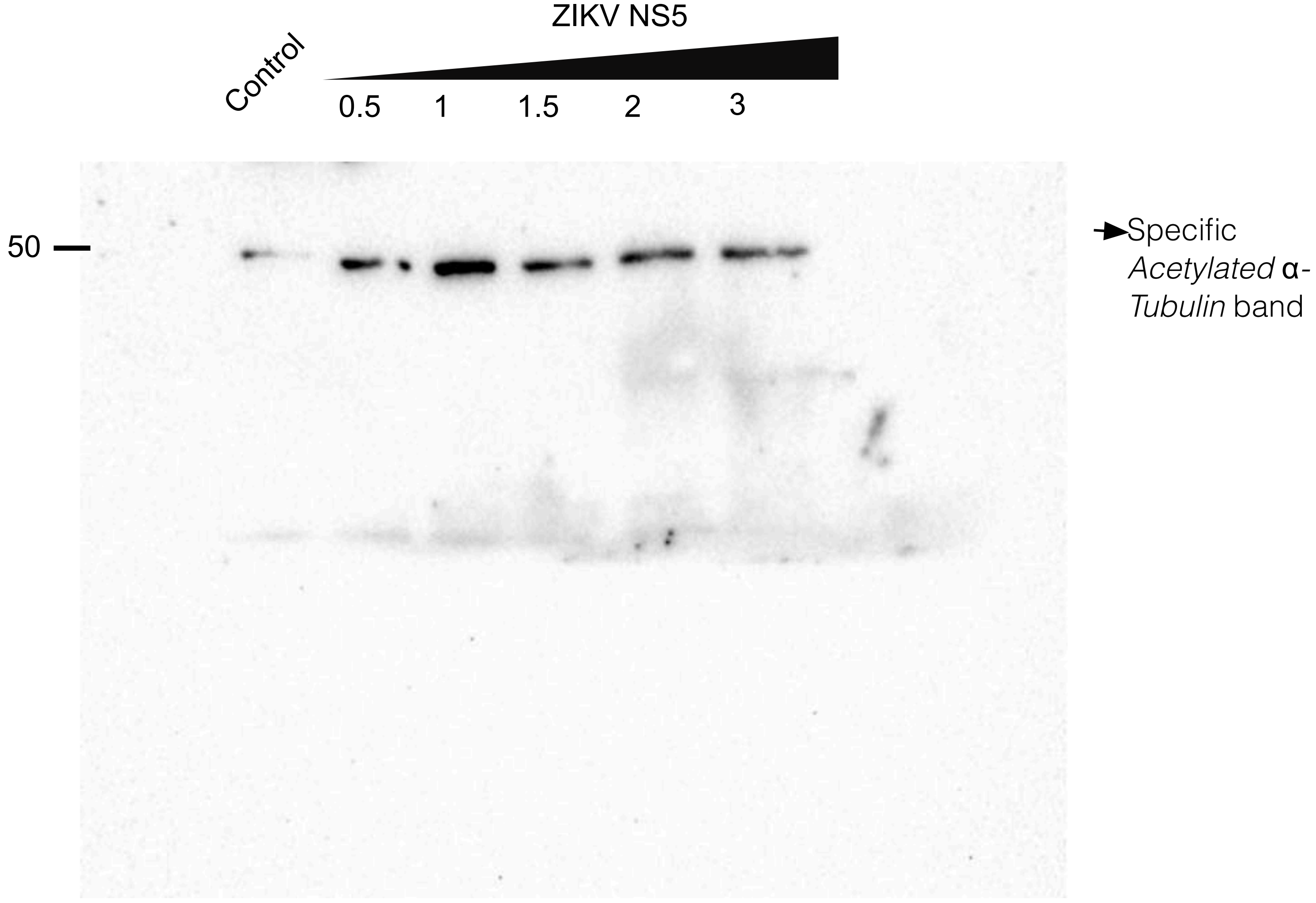

Figure S2C. *Replicate 5 myc complete gel*

Western-blot associated with Figure S2C
Pérez-Yanes, S., et al.

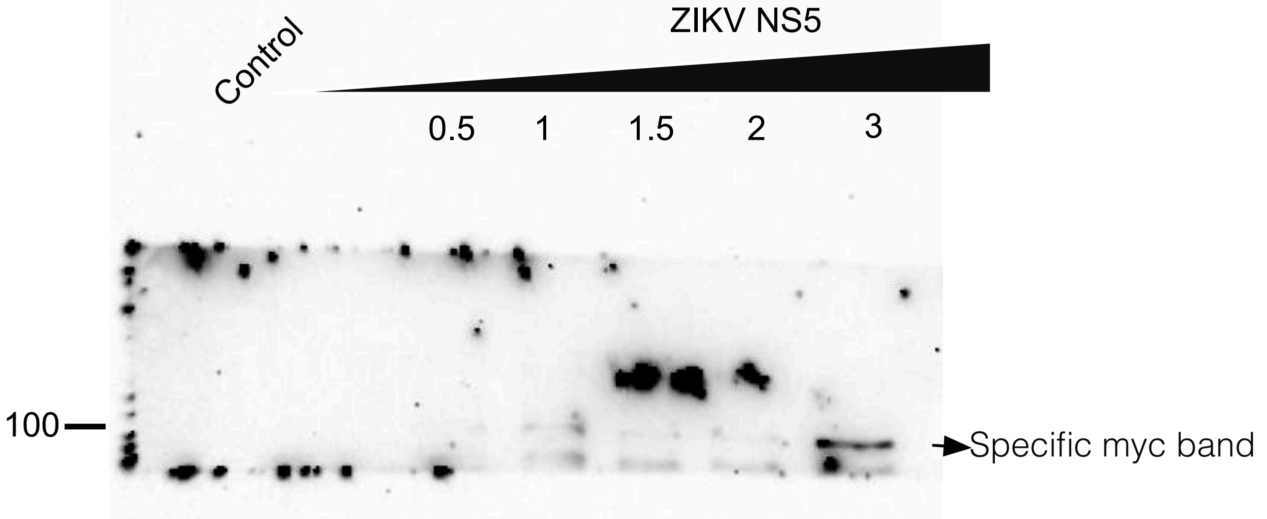

Figure S2C. *Replicate 5 Total α-Tubulin complete gel*

Western-blot associated with Figure 2C
Pérez-Yanes, S., et al.

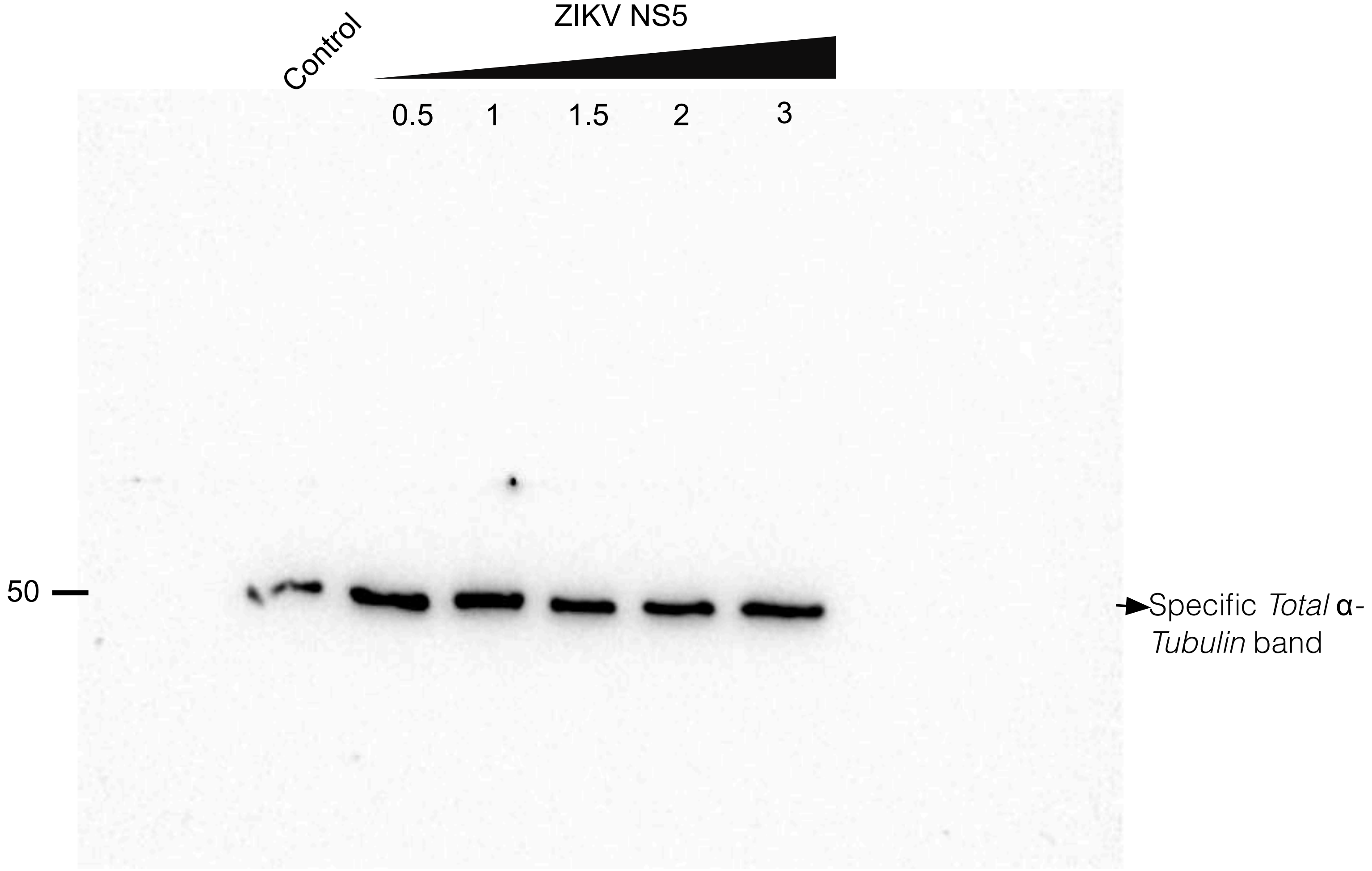

Figure S2C. *Replicate 5 Acetylated* α*-Tubulin complete gel*

Western-blot associated with Figure S2C Pérez-Yanes, S., et al.

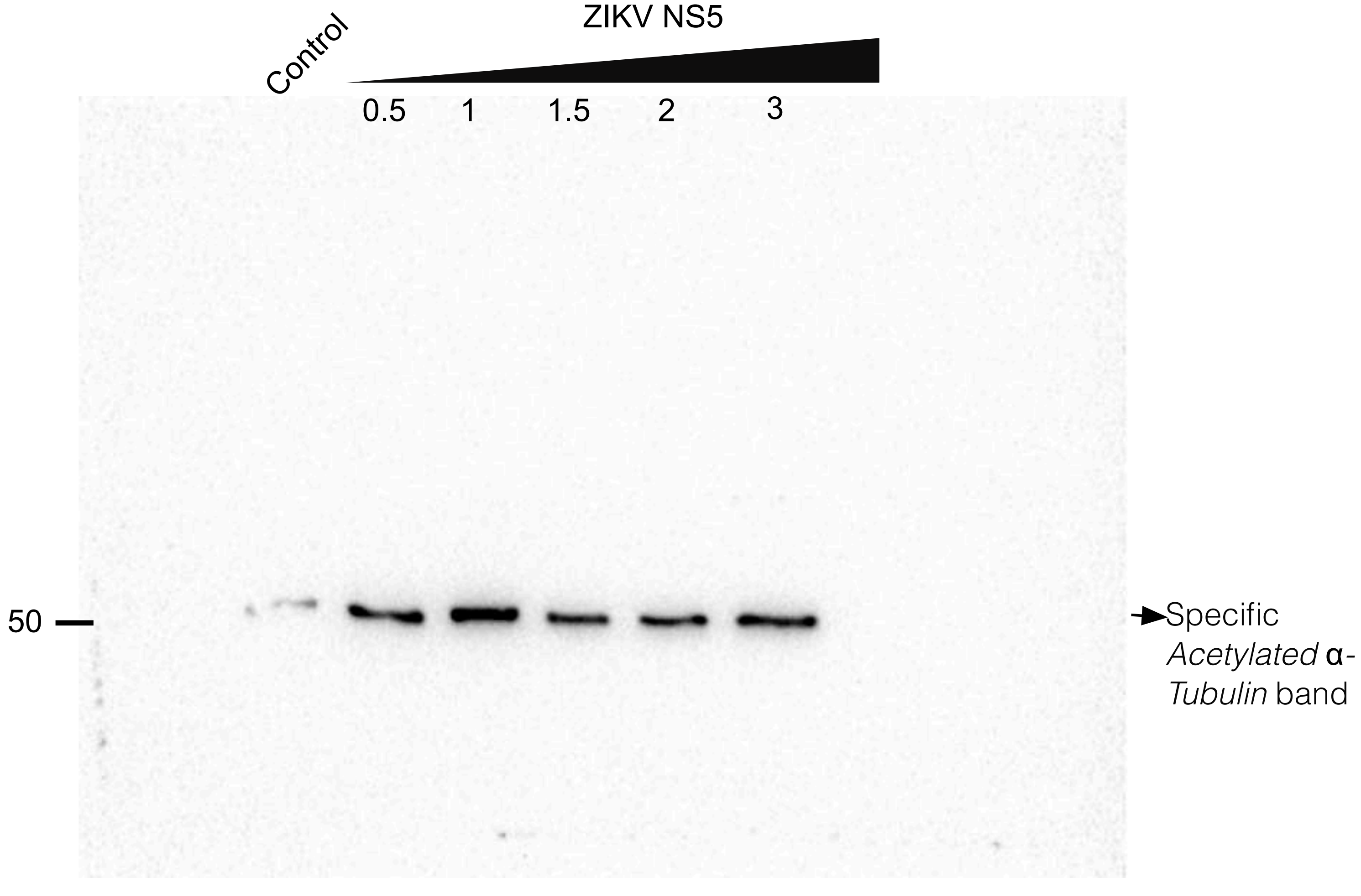

Figure S2C. *Replicate 6 myc complete gel*

Western-blot associated with Figure S2C
Pérez-Yanes, S., et al.

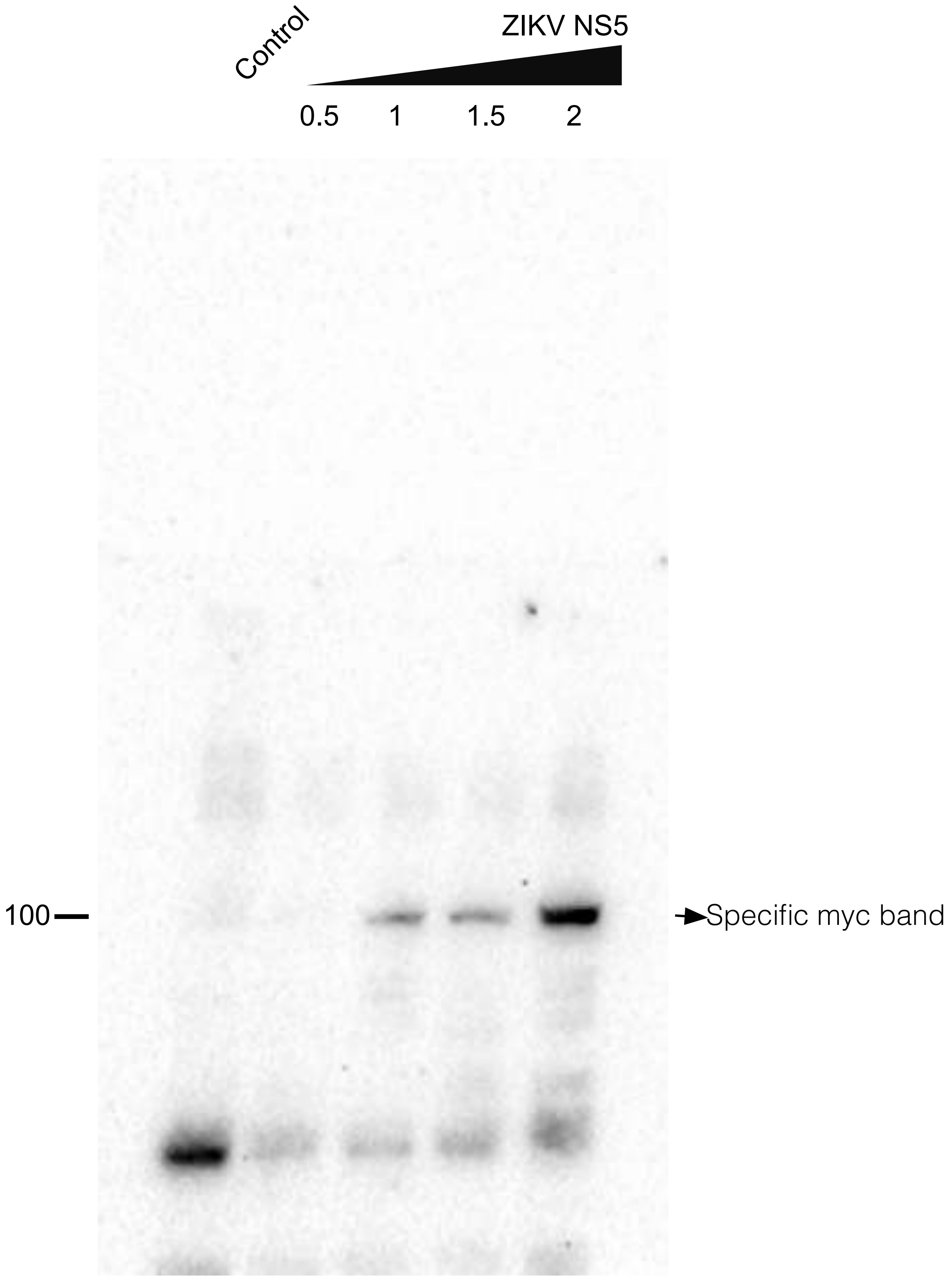

Figure S2C. *Replicate 6 Total* α-*Tubulin complete gel*

Western-blot associated with Figure 2C
Pérez-Yanes, S., et al.

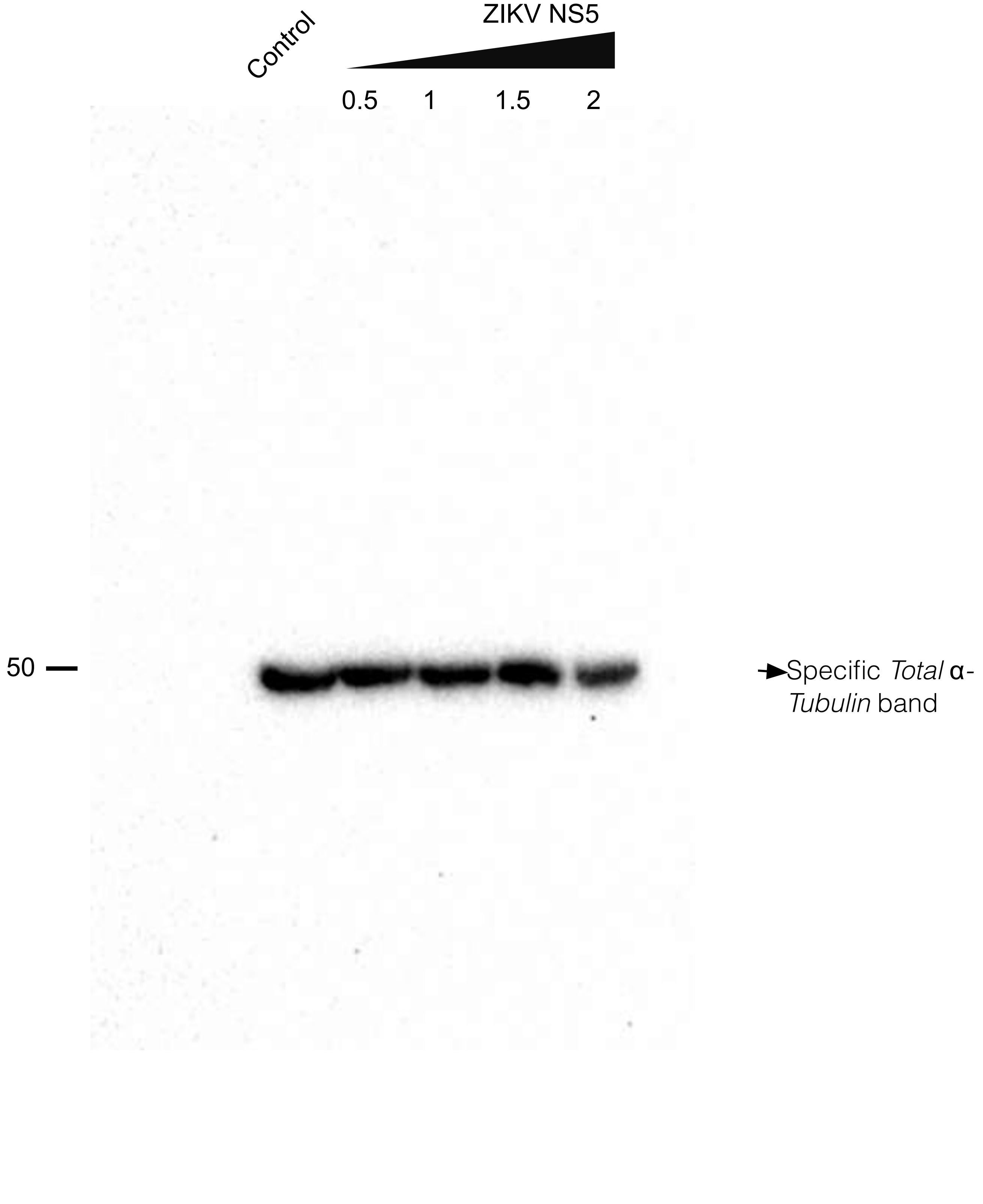

Figure S2C. *Replicate 6 Acetylated α-Tubulin complete gel*

Western-blot associated with Figure S2C Pérez-Yanes, S., et al.

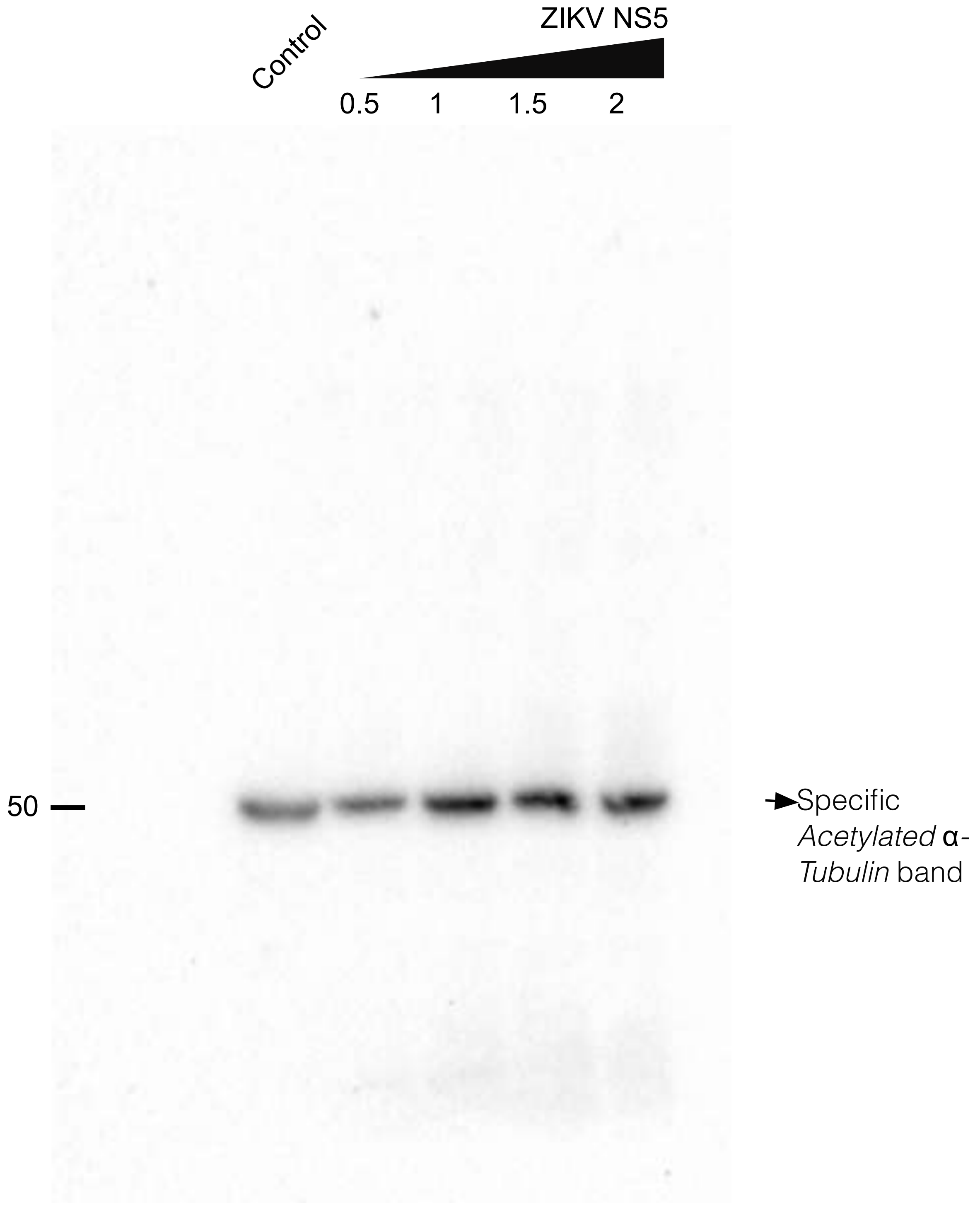

Figure S2C. *Replicate 7 myc complete gel*

Western-blot associated with Figure S2C
Pérez-Yanes, S., et al.

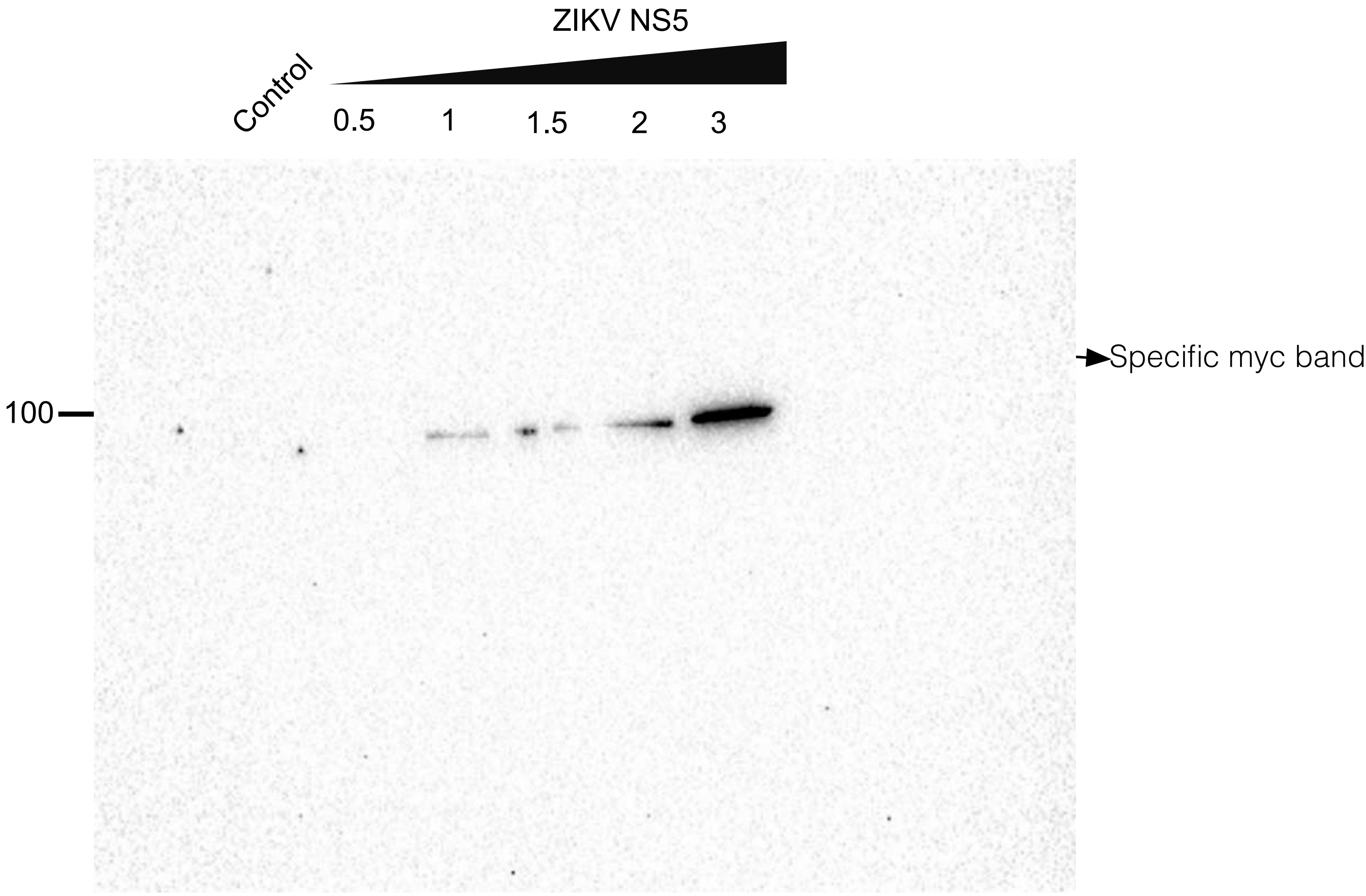

Figure S2C. *Replicate 7 Total* α*-Tubulin complete gel*

Western-blot associated with Figure 3C Pérez-Yanes, S., et al.

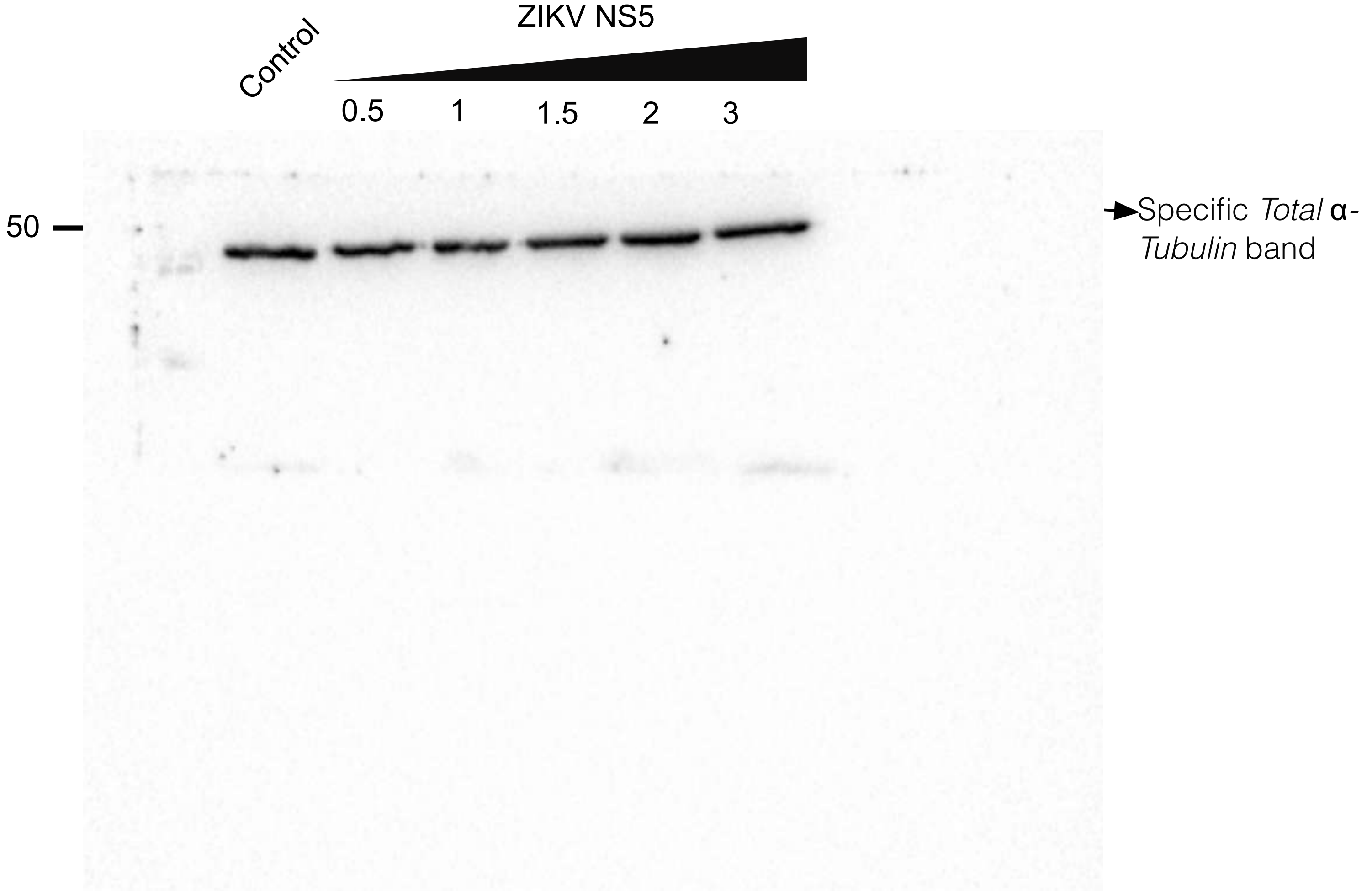

Figure S2C. *Replicate 7 Acetylated α-Tubulin complete gel*

Western-blot associated with Figure S2C Pérez-Yanes, S., et al.

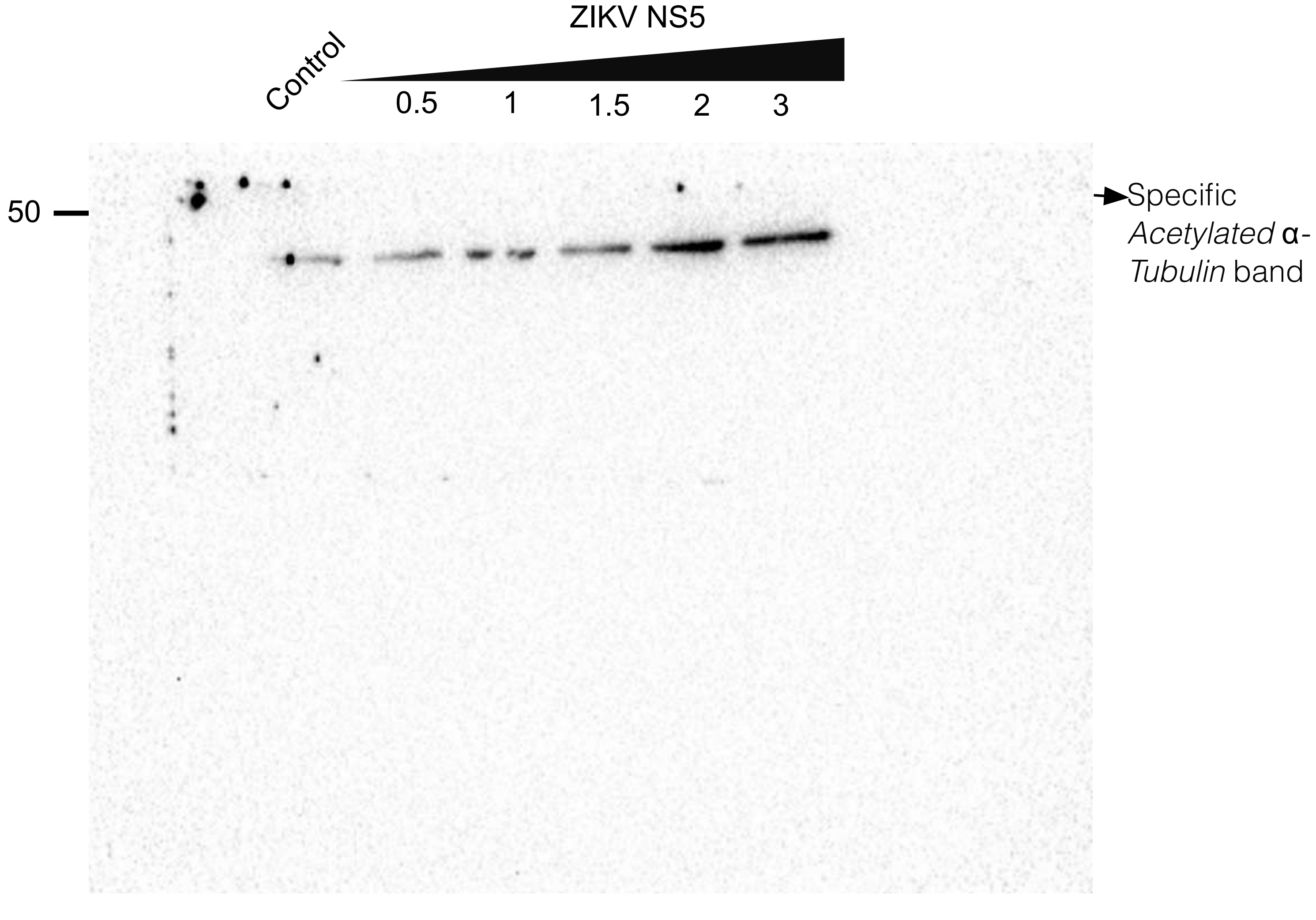

Figure S2C. *Replicate 8 myc complete gel*

Western-blot associated with Figure S2C
Pérez-Yanes, S., et al.

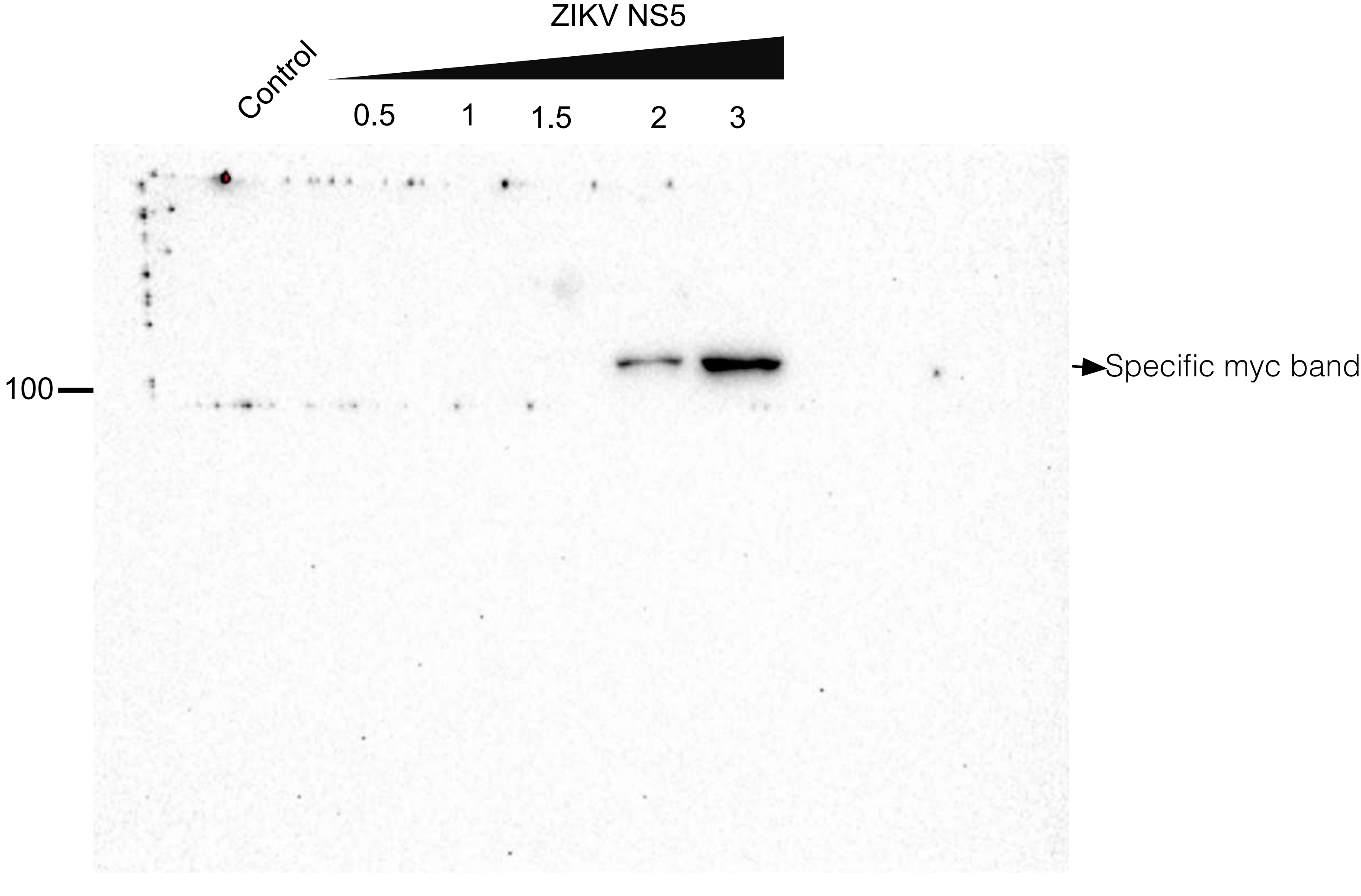

Figure S2C. *Replicate 8 Total* α*-Tubulin complete gel*

Western-blot associated with Figure 2C
Pérez-Yanes, S., et al.

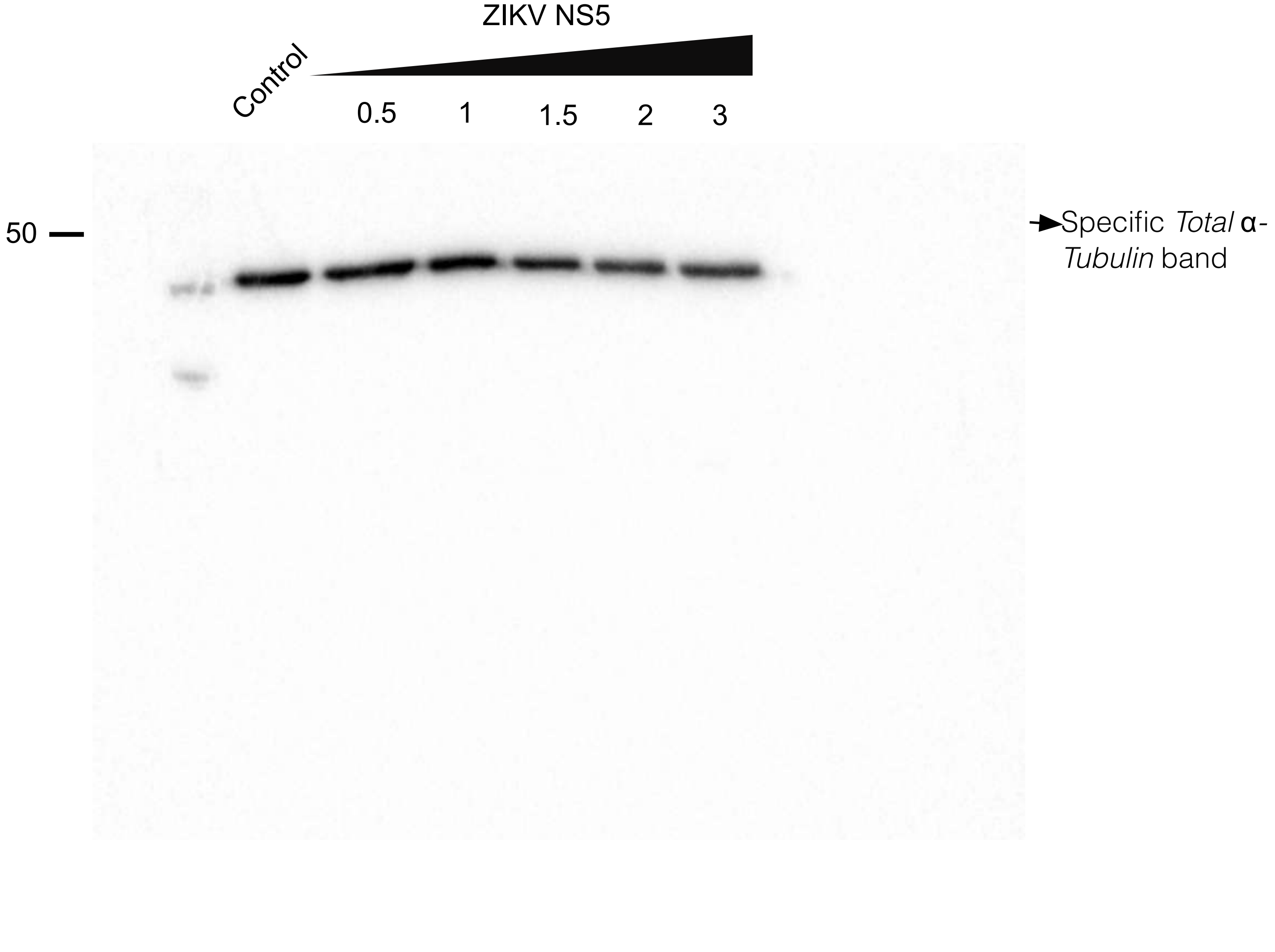

Figure S2C. *Replicate 8 Acetylated* α*-Tubulin complete gel*

Western-blot associated with Figure S2C Pérez-Yanes, S., et al.

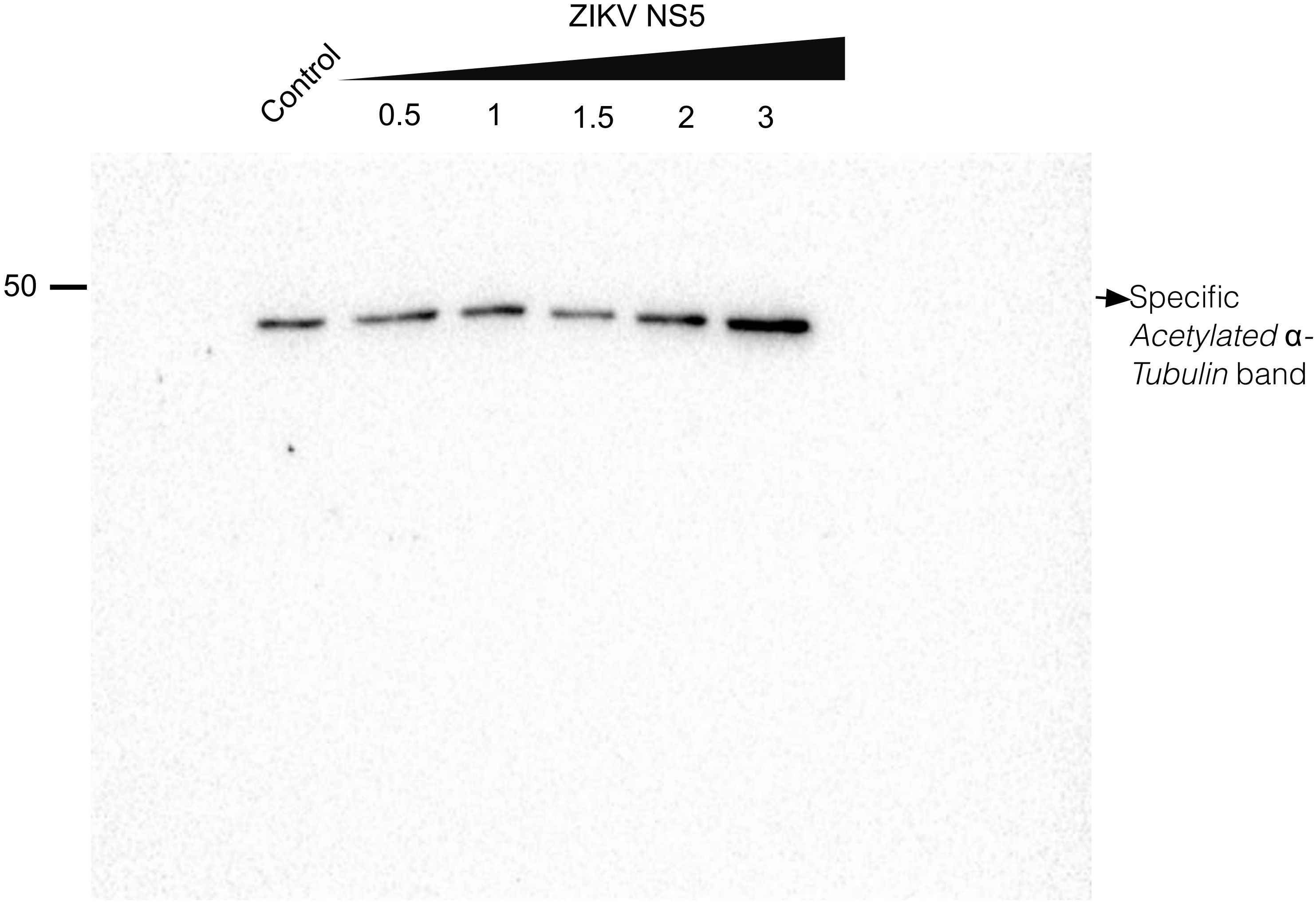

Figure S2C. *Replicate 9 myc complete gel*

Western-blot associated with Figure S2C
Pérez-Yanes, S., et al.

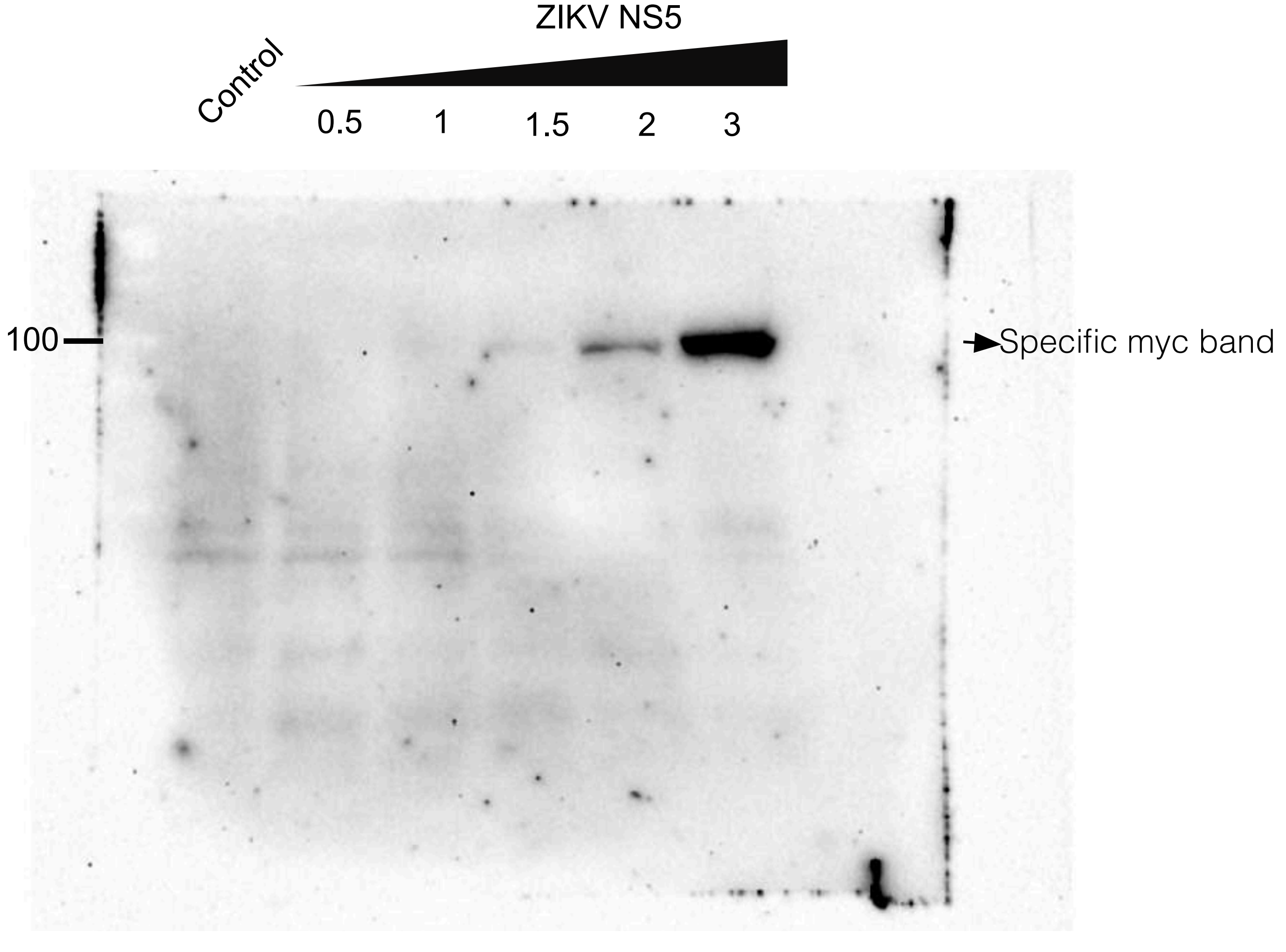

Figure S2C. *Replicate 9 Total* α*-Tubulin complete gel*

Western-blot associated with Figure 2C
Pérez-Yanes, S., et al.

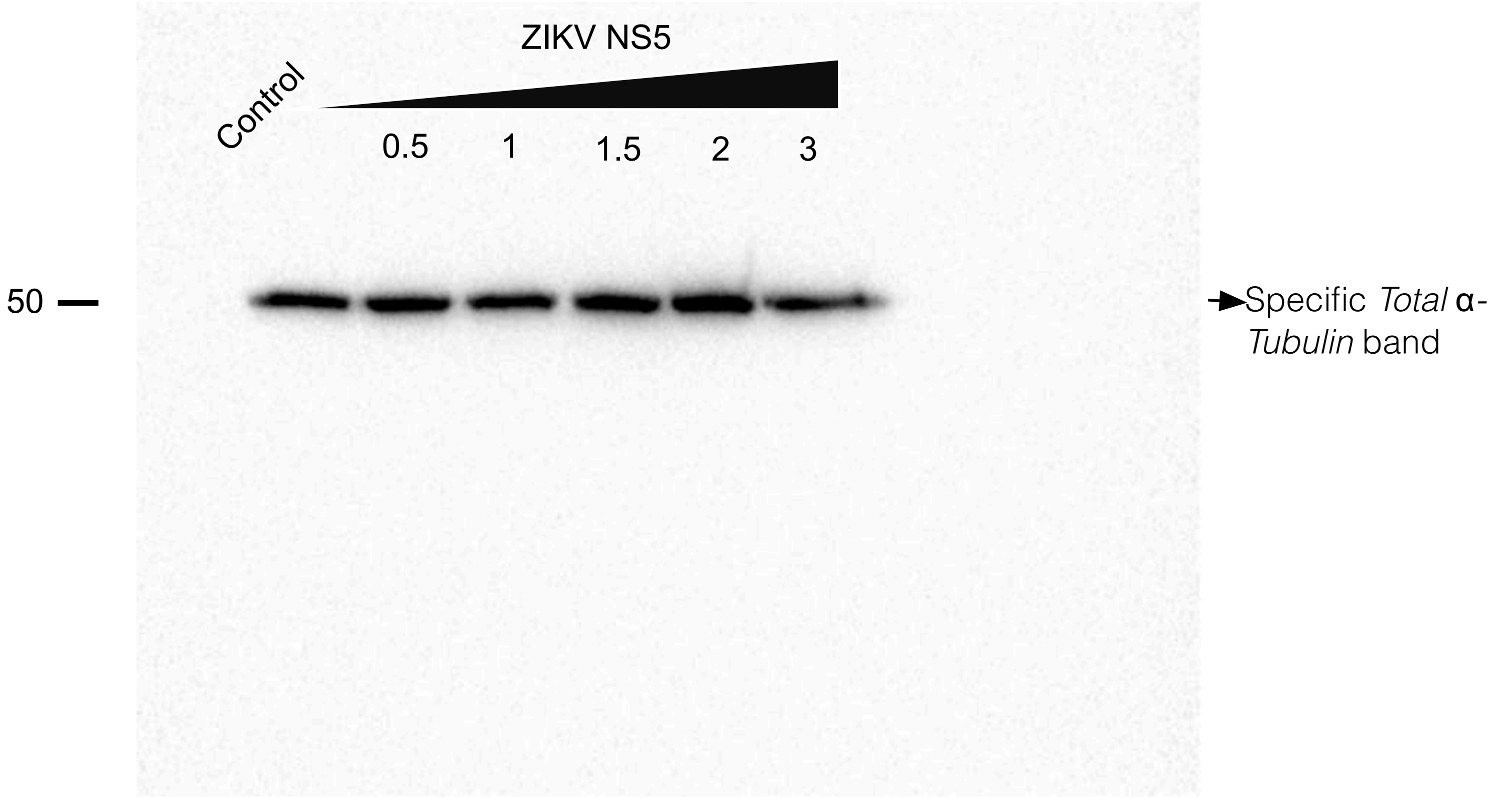

Figure S2C. *Replicate 9 Acetylated* α-*Tubulin complete gel*

Western-blot associated with Figure S2C
Pérez-Yanes, S., et al.

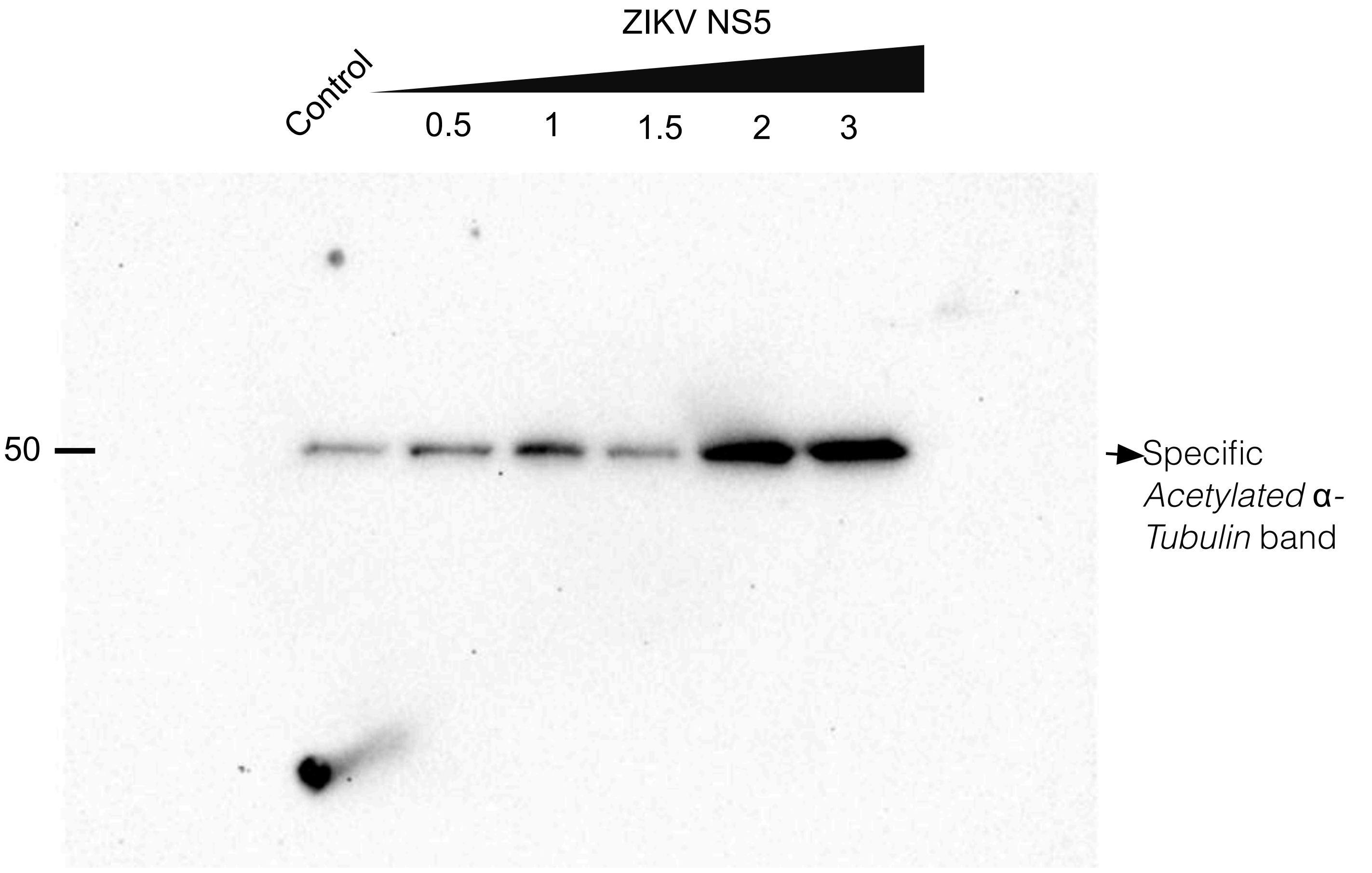

Figura S2D. Western-blot replicates associated with Figure S2D.

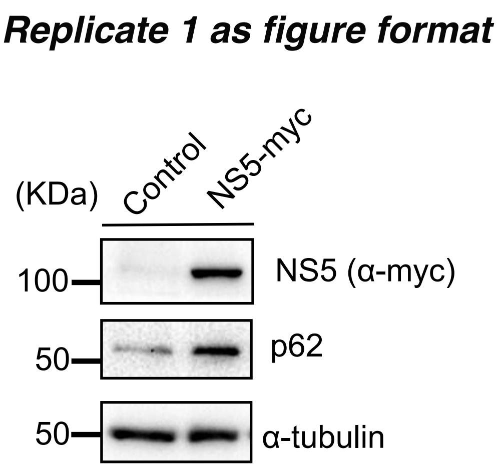

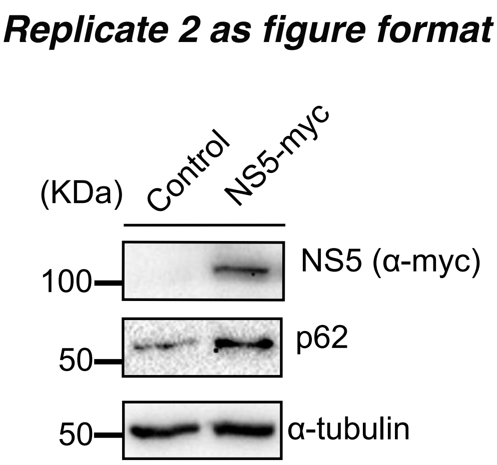

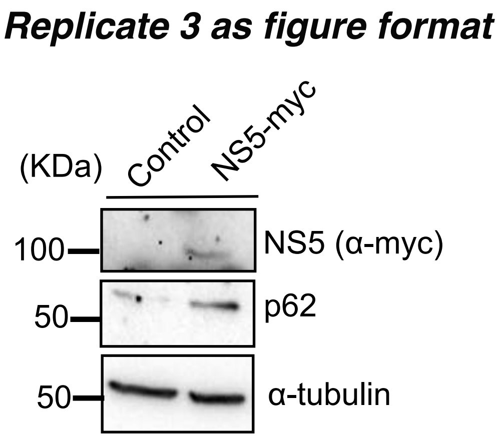

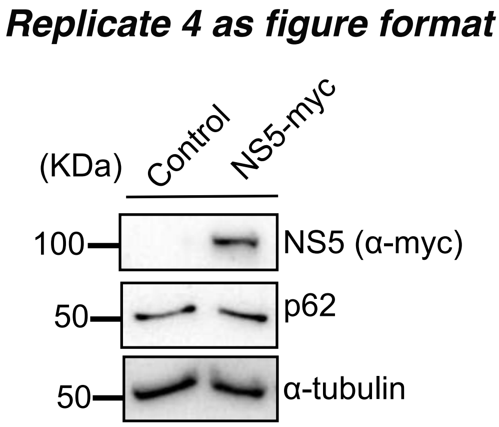

Figure S2D. *Replicate 1 myc complete gel*

Western-blot associated with Figure S2D
Pérez-Yanes, S., et al.

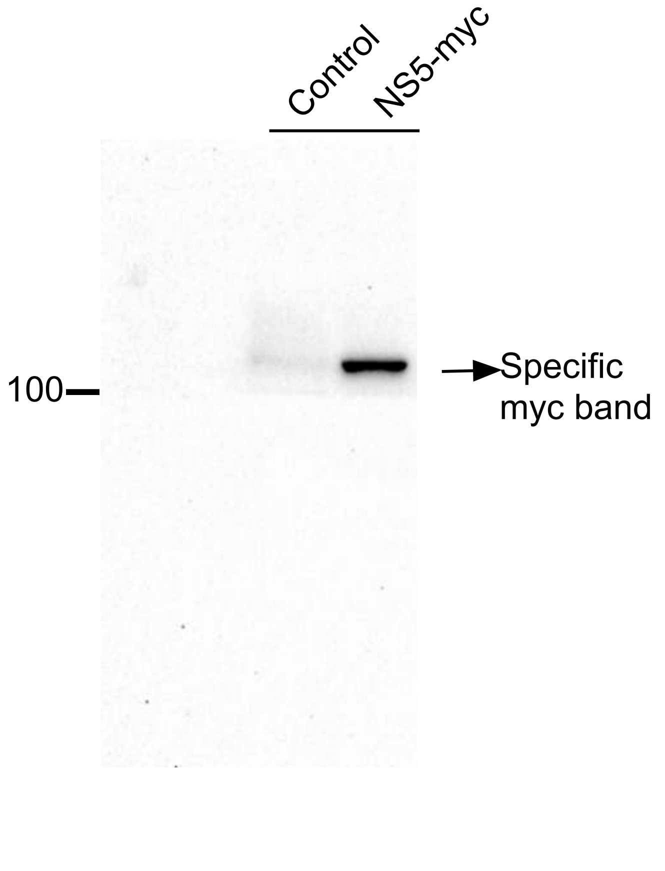

Figure S2D. *Replicate 1 p62 complete gel*

Western-blot associated with Figure S2D
Pérez-Yanes, S., et al.

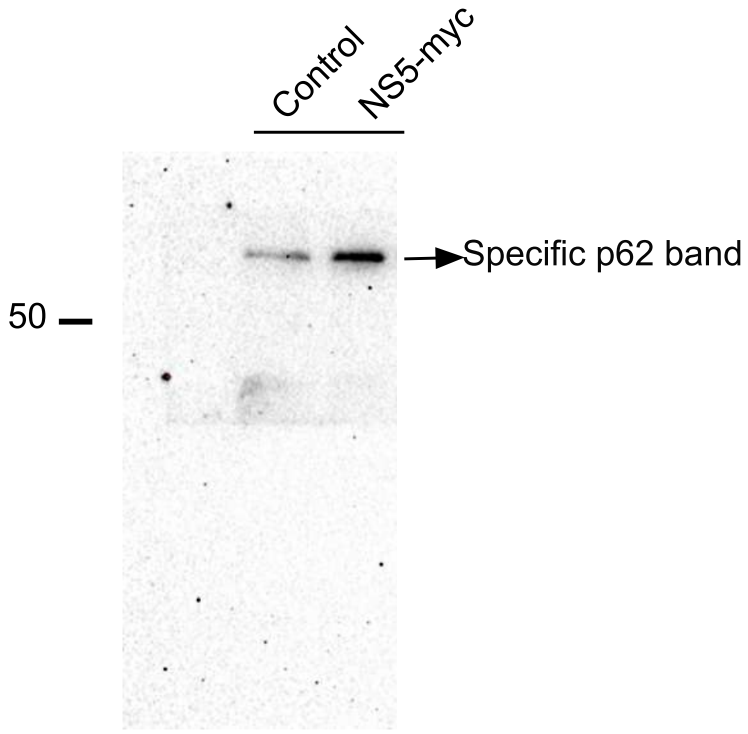

Figure S2D. *Replicate 1 Total α-Tubulin complete gel*

Western-blot associated with Figure S2D
Pérez-Yanes, S., et al.

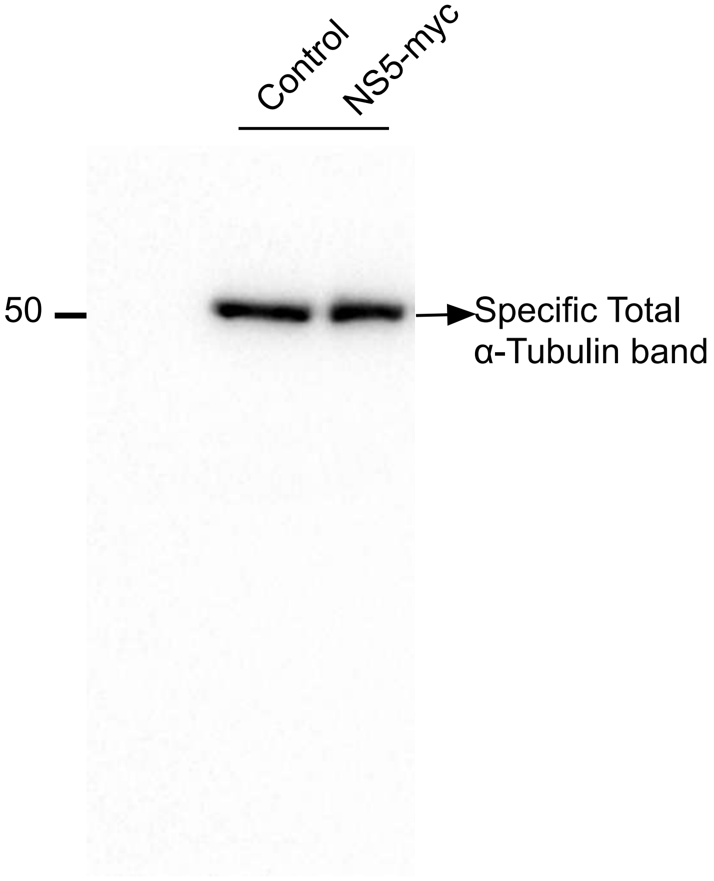

Figure S2D. *Replicate 2 myc complete gel*

Western-blot associated with Figure S2D
Pérez-Yanes, S., et al.

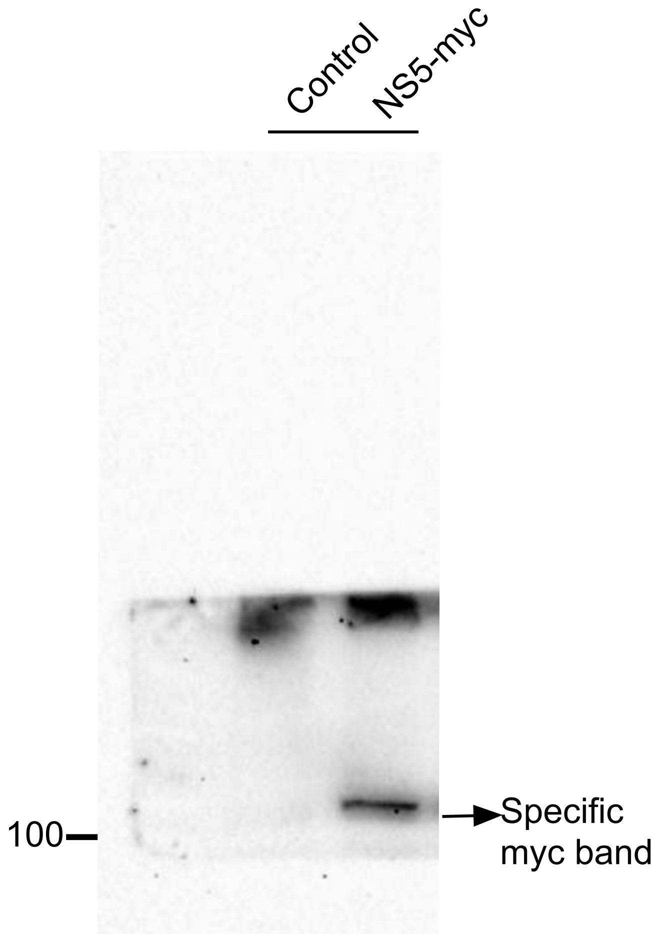

Figure S2D. *Replicate 2 p62 complete gel*

Western-blot associated with Figure S2D
Pérez-Yanes, S., et al.

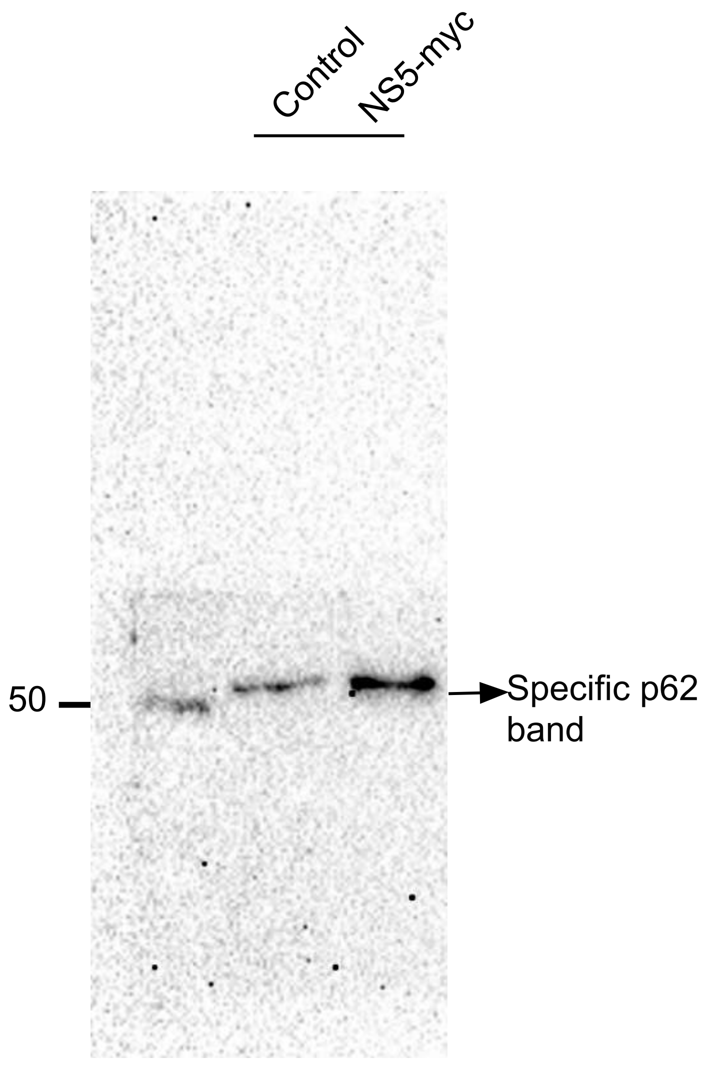

Figure S2D. *Replicate 2 Total α-Tubulin complete gel*

Western-blot associated with Figure S2D
Pérez-Yanes, S., et al.

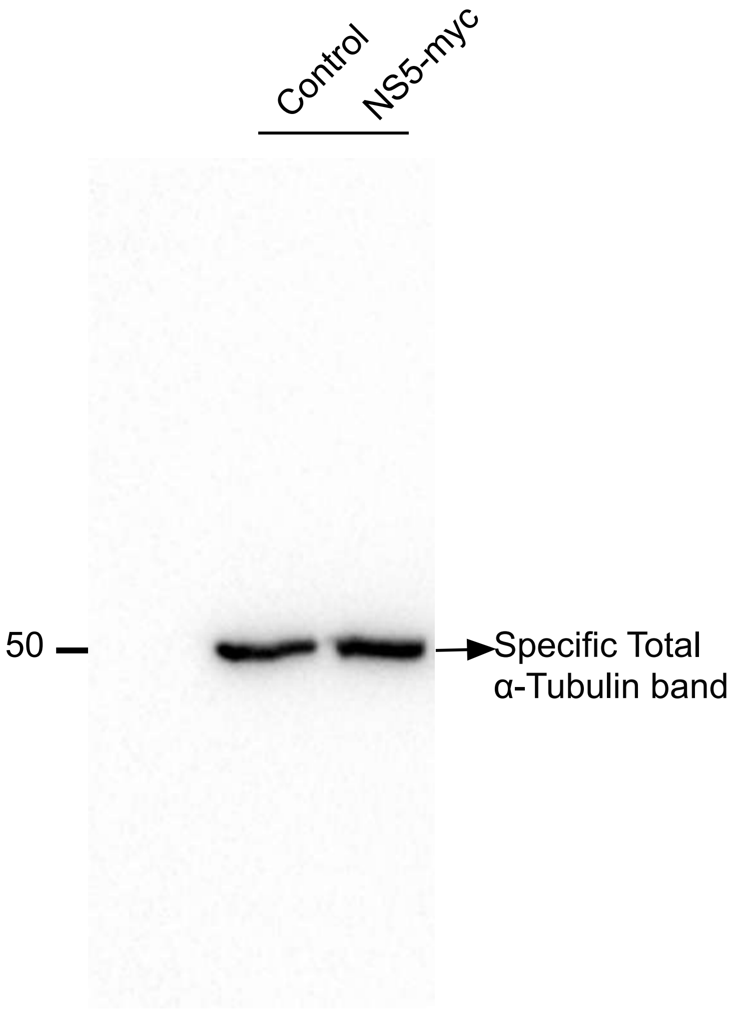

Figure S2D. *Replicate 3 myc complete gel*

Western-blot associated with Figure S2D
Pérez-Yanes, S., et al.

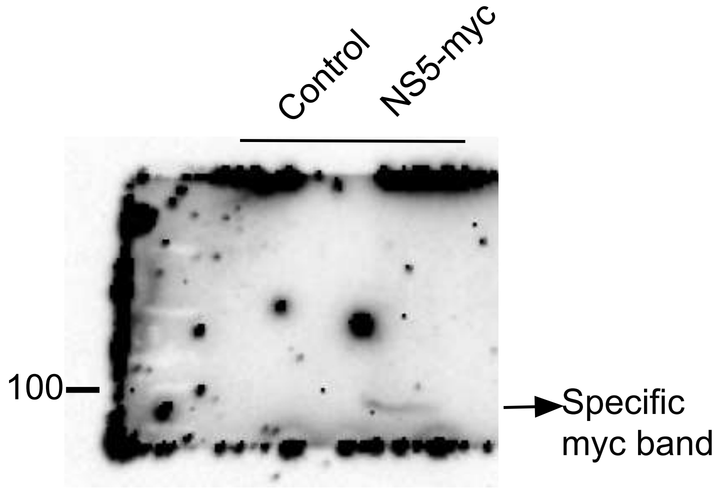

Figure S2D. *Replicate 3 p62 complete gel*

Western-blot associated with Figure S2D
Pérez-Yanes, S., et al.

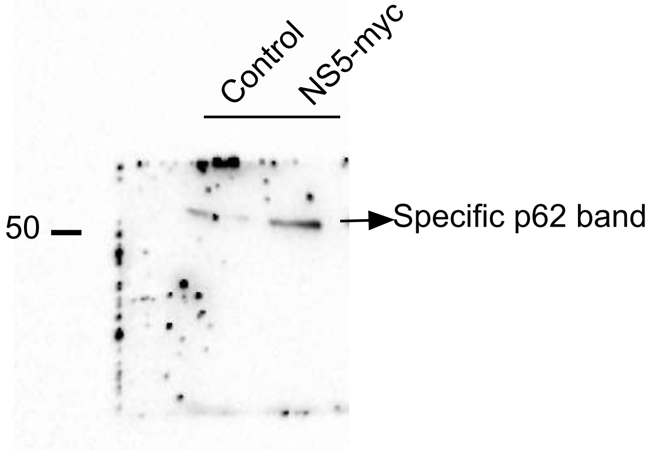

Figure S2D. *Replicate 3 Total α-Tubulin complete gel*

Western-blot associated with Figure S2D
Pérez-Yanes, S., et al.

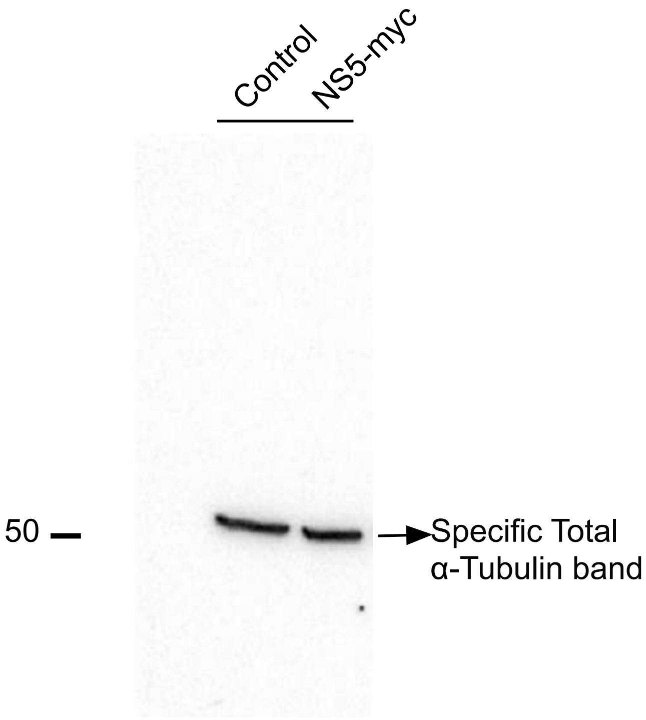

Figure S2D. *Replicate 4 myc complete gel*

Western-blot associated with Figure S2D
Pérez-Yanes, S., et al.

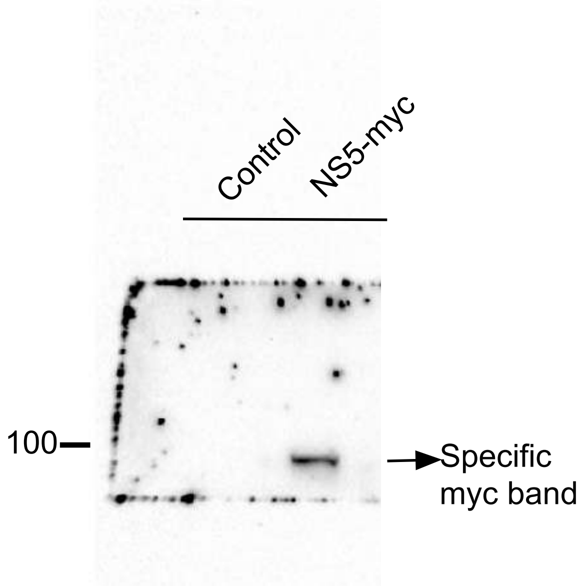

Figure S2D. *Replicate 4 p62 complete gel*

Western-blot associated with Figure S2D
Pérez-Yanes, S., et al.

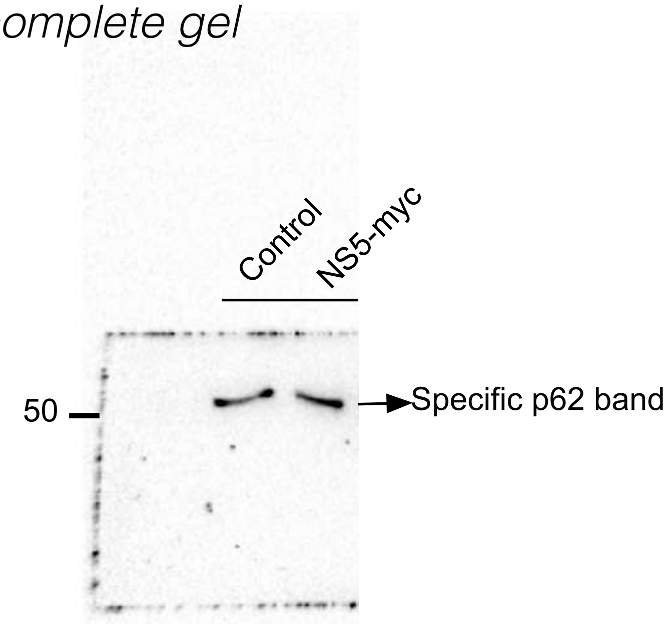

Figure S2D. *Replicate 4 Total α-Tubulin complete gel*

Western-blot associated with Figure S2D
Pérez-Yanes, S., et al.

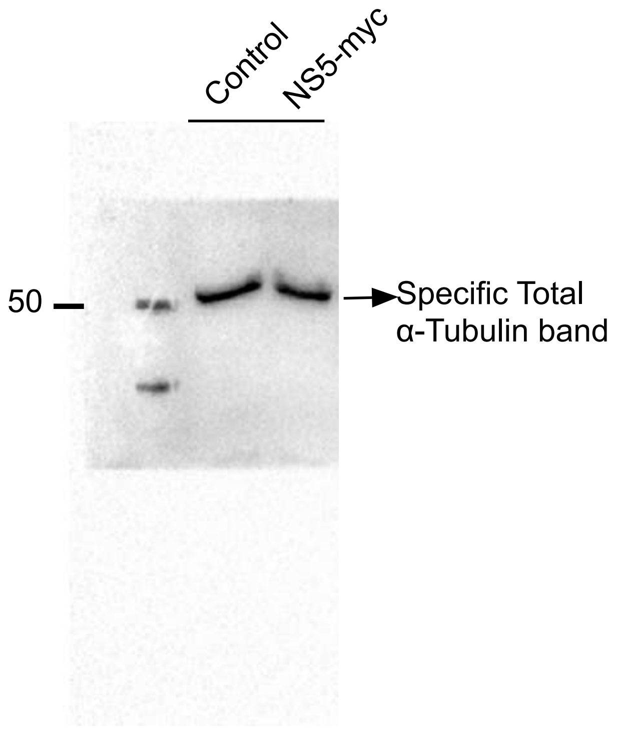

Figure S2E. Western-blot replicates associated with Figure 2E.

Replicate 1 as figure format

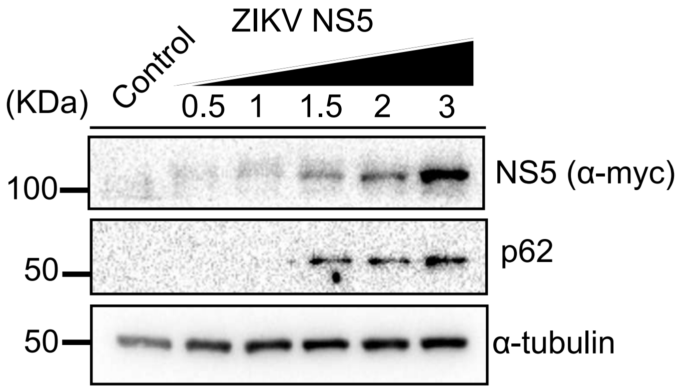

Replicate 2 as figure format

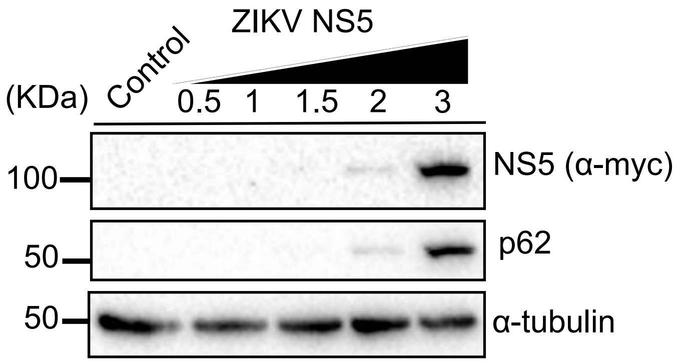

Replicate 3 as figure format

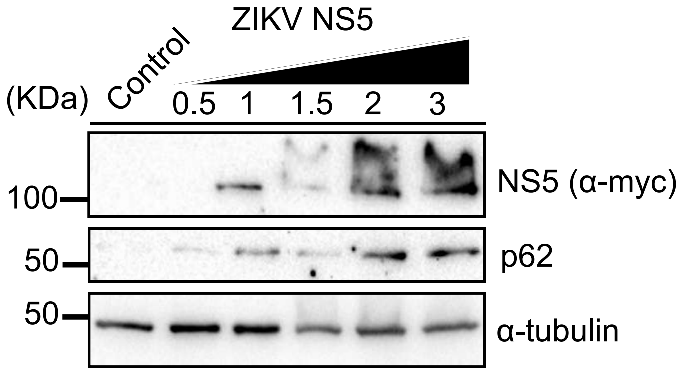

Figure S2E. *Replicate 1 myc complete gel*

Western-blot associated with Figure S2E
Pérez-Yanes, S., et al.

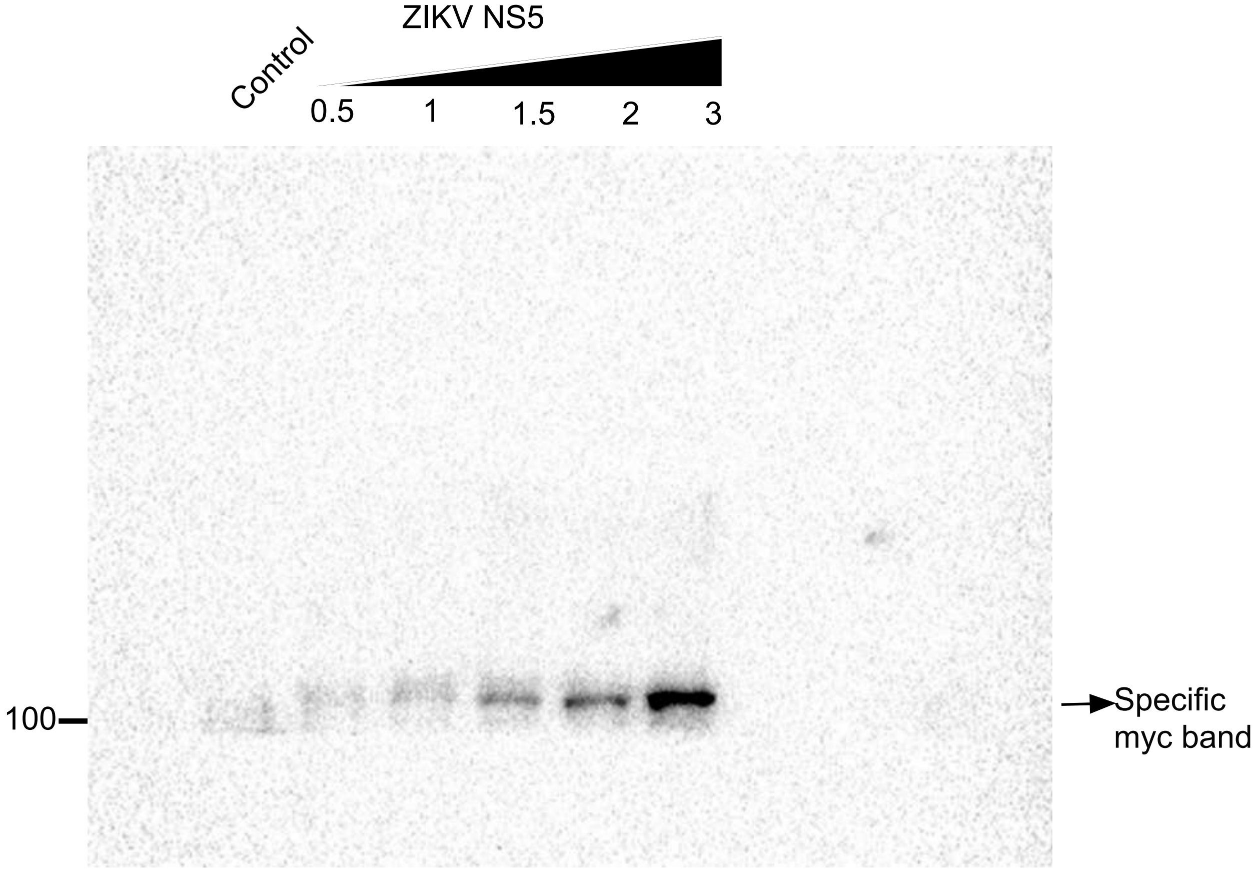

Figure S2E. *Replicate 1 p62 complete gel*

Western-blot associated with Figure S2E
Pérez-Yanes, S., et al.

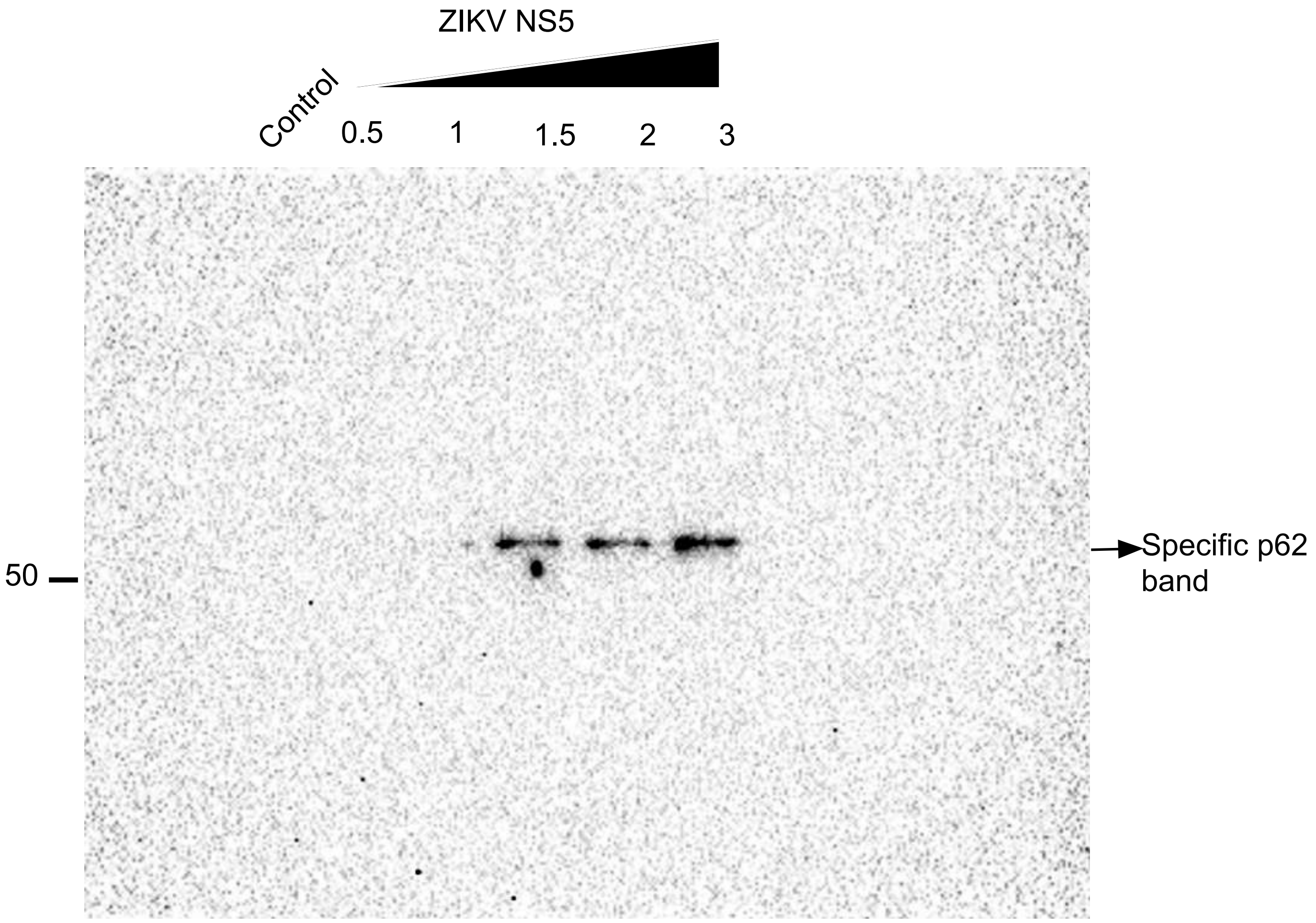

Figure S2E. *Replicate 1 Total α-Tubulin complete gel*

Western-blot associated with Figure S2E
Pérez-Yanes, S., et al.

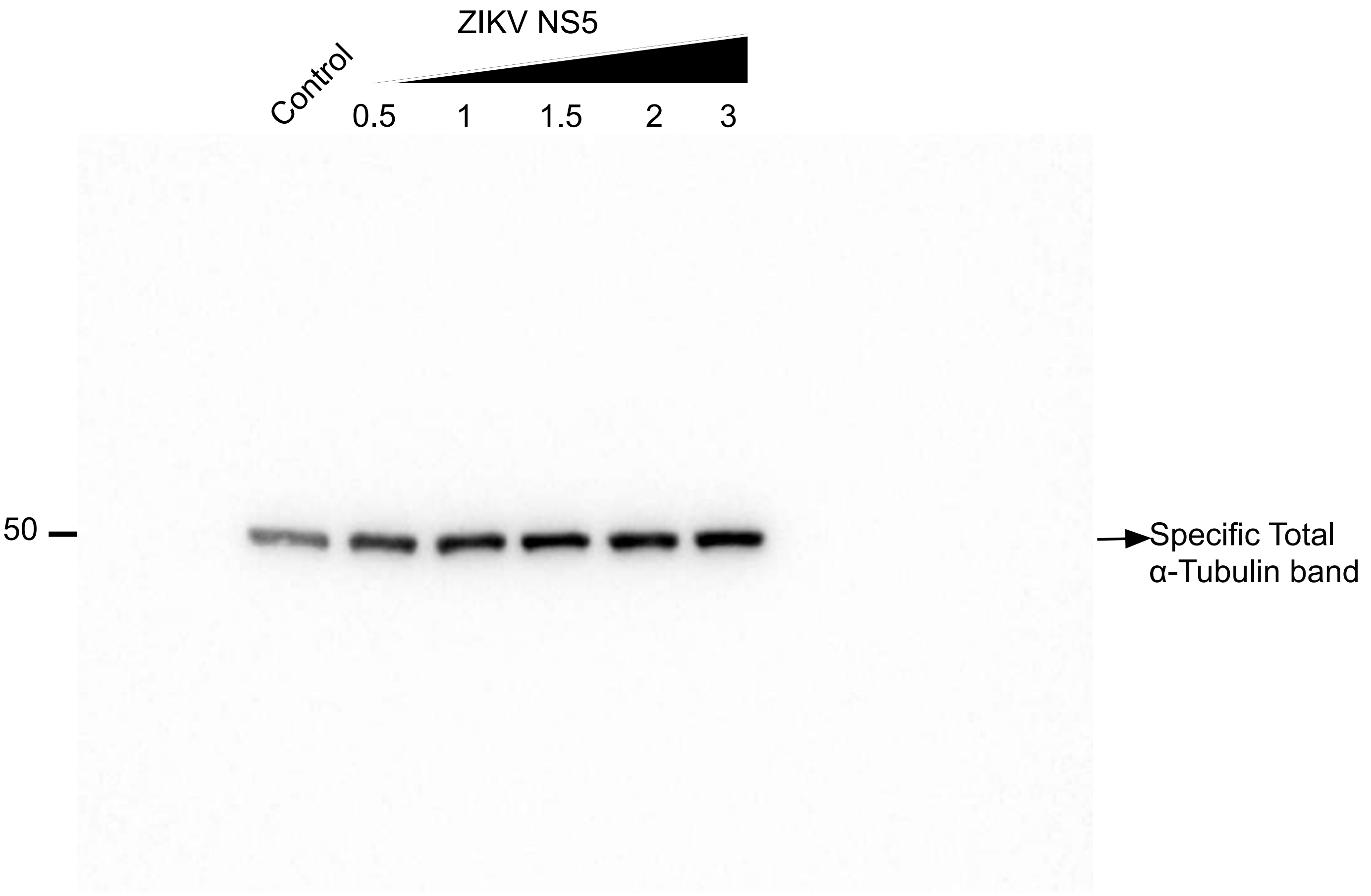

Figure S2E. *Replicate 2 myc complete gel*

Western-blot associated with Figure S2E
Pérez-Yanes, S., et al.

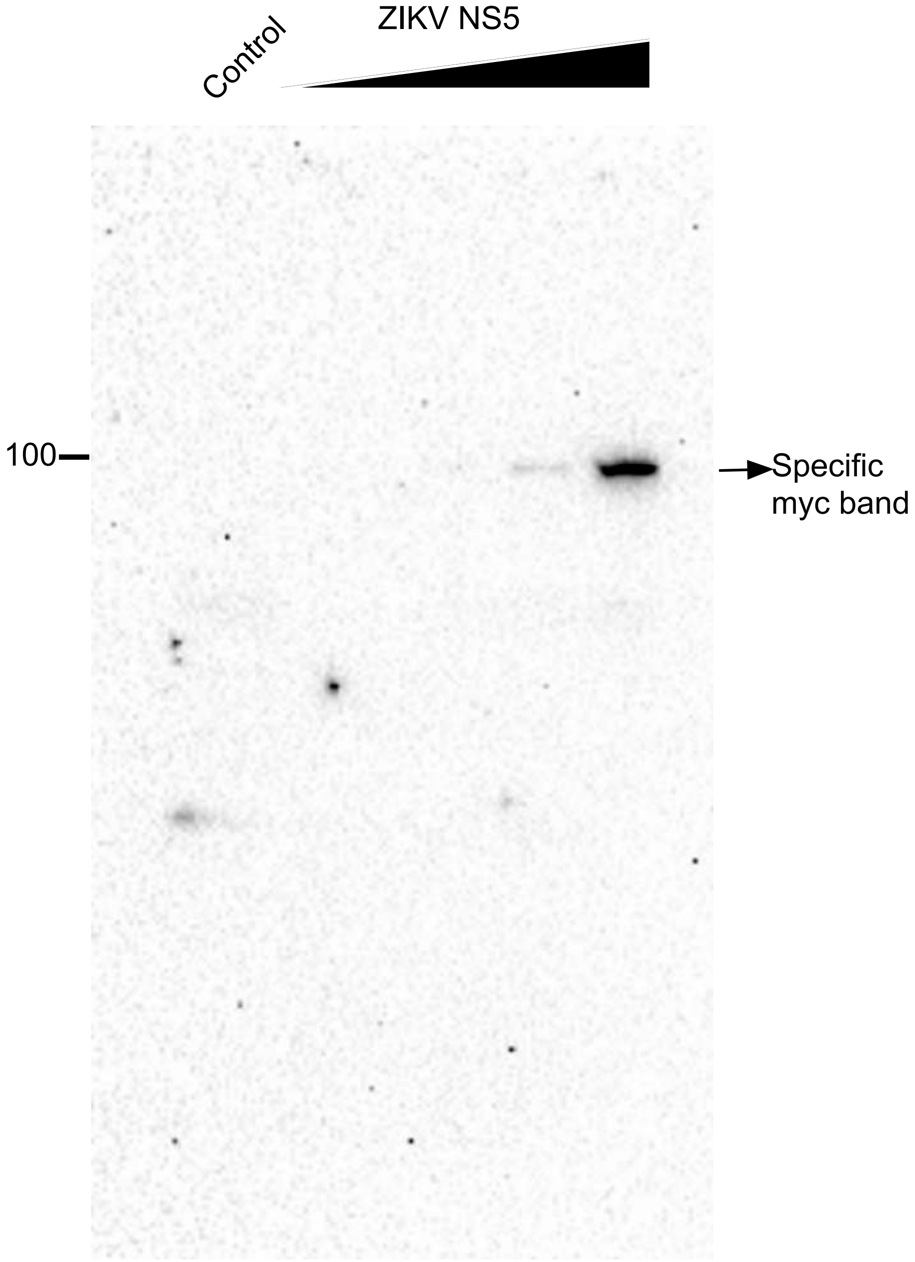

Figure S2E. *Replicate 2 p62 complete gel*

Western-blot associated with Figure S2E
Pérez-Yanes, S., et al.

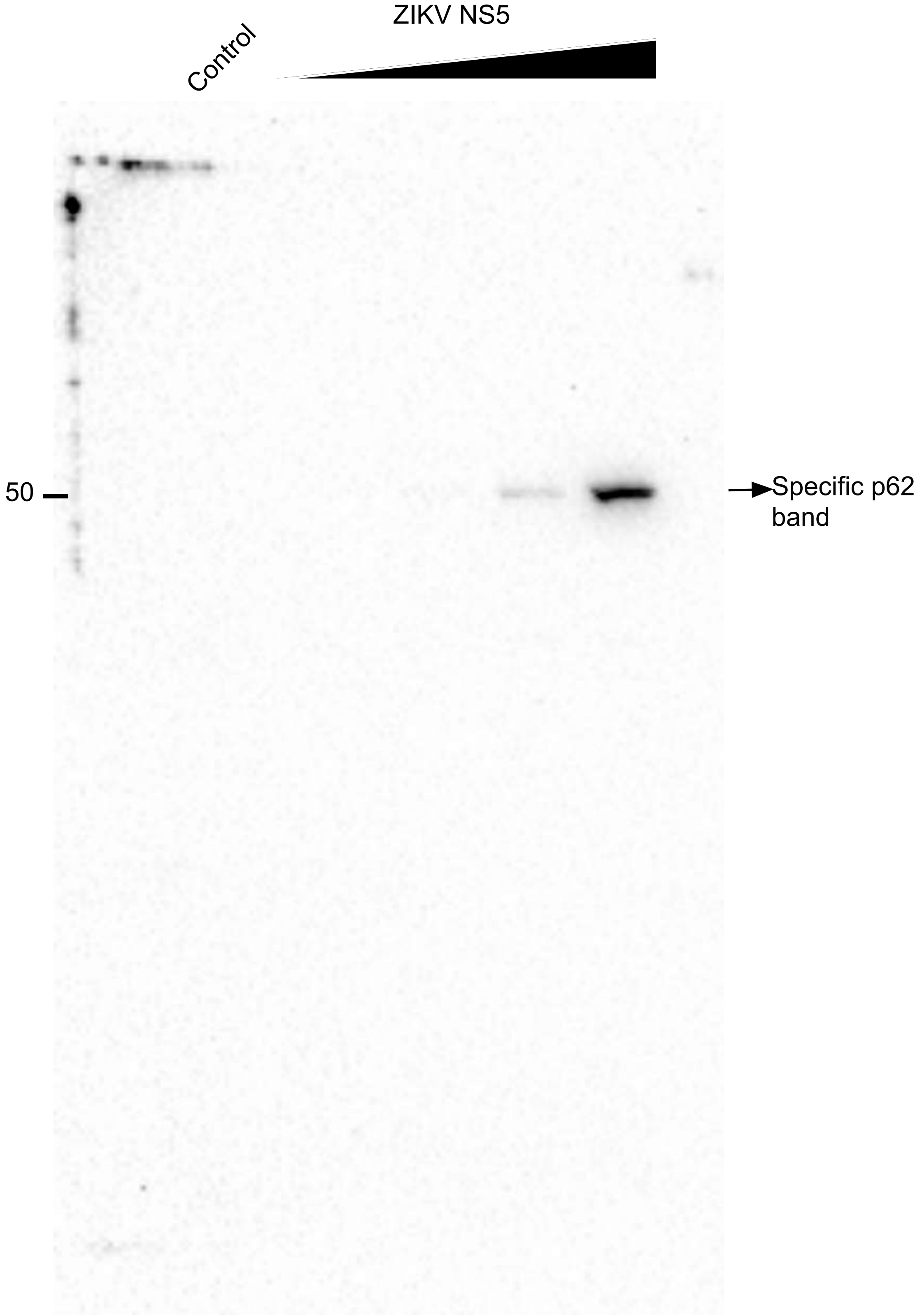

Figure S2E. *Replicate 2 Total α-Tubulin complete gel*

Western-blot associated with Figure S2E
Pérez-Yanes, S., et al.

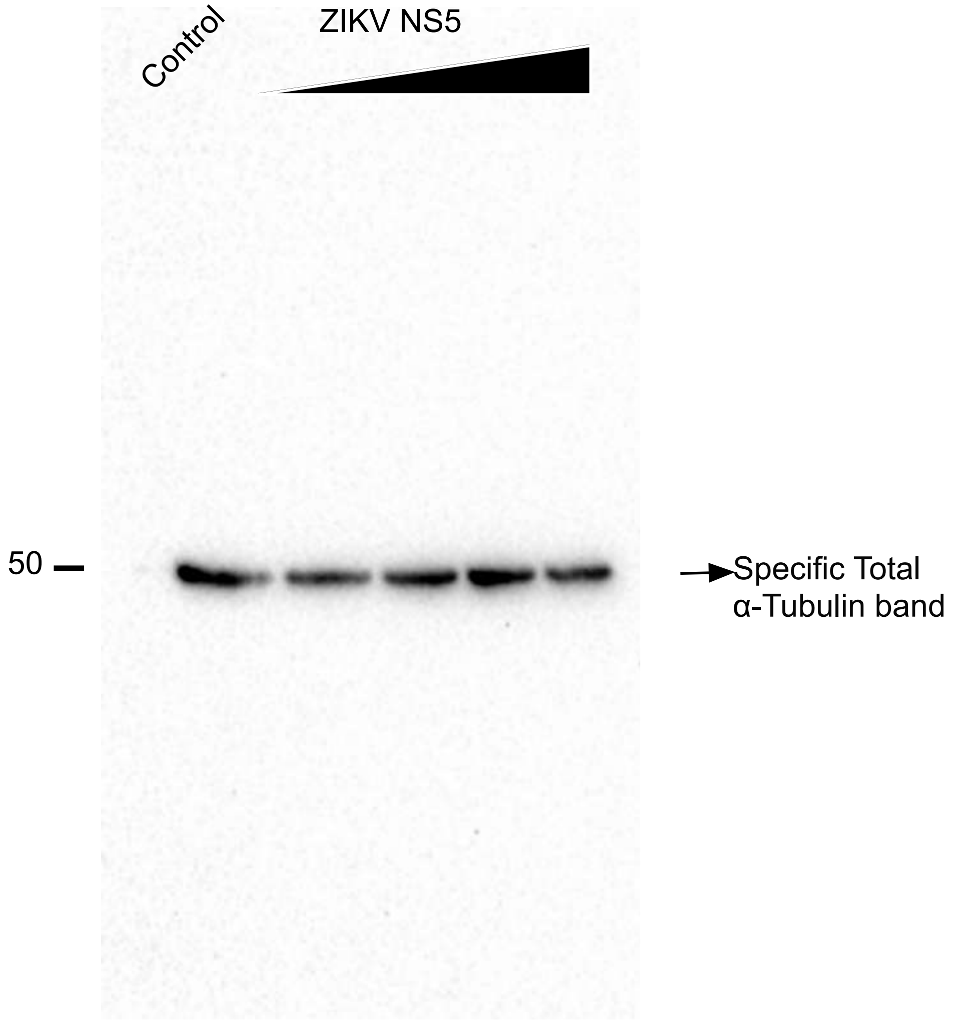

Figure S2E. *Replicate 3 myc complete gel*

Western-blot associated with Figure S2E
Pérez-Yanes, S., et al.

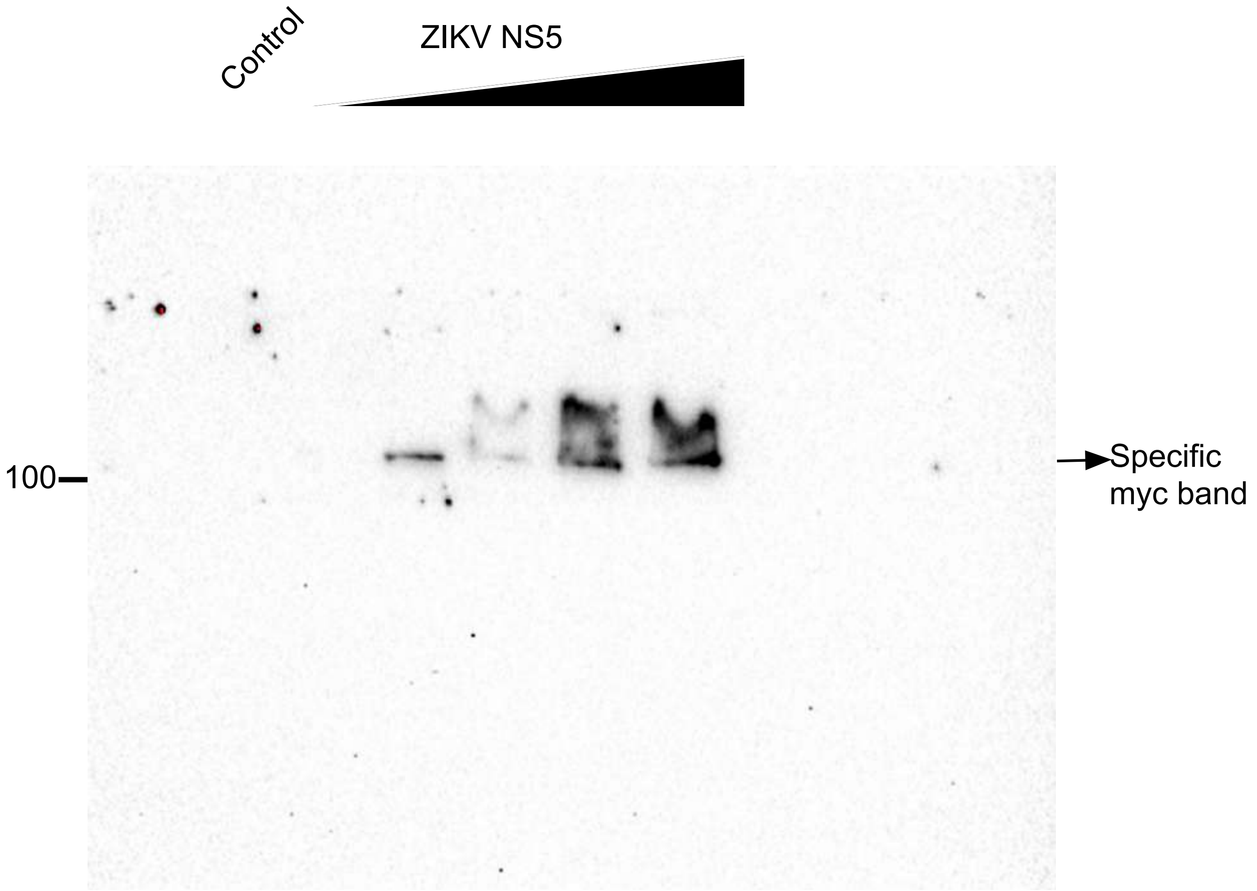

Figure S2E. *Replicate 3 p62 complete gel*

Western-blot associated with Figure S2E
Pérez-Yanes, S., et al.

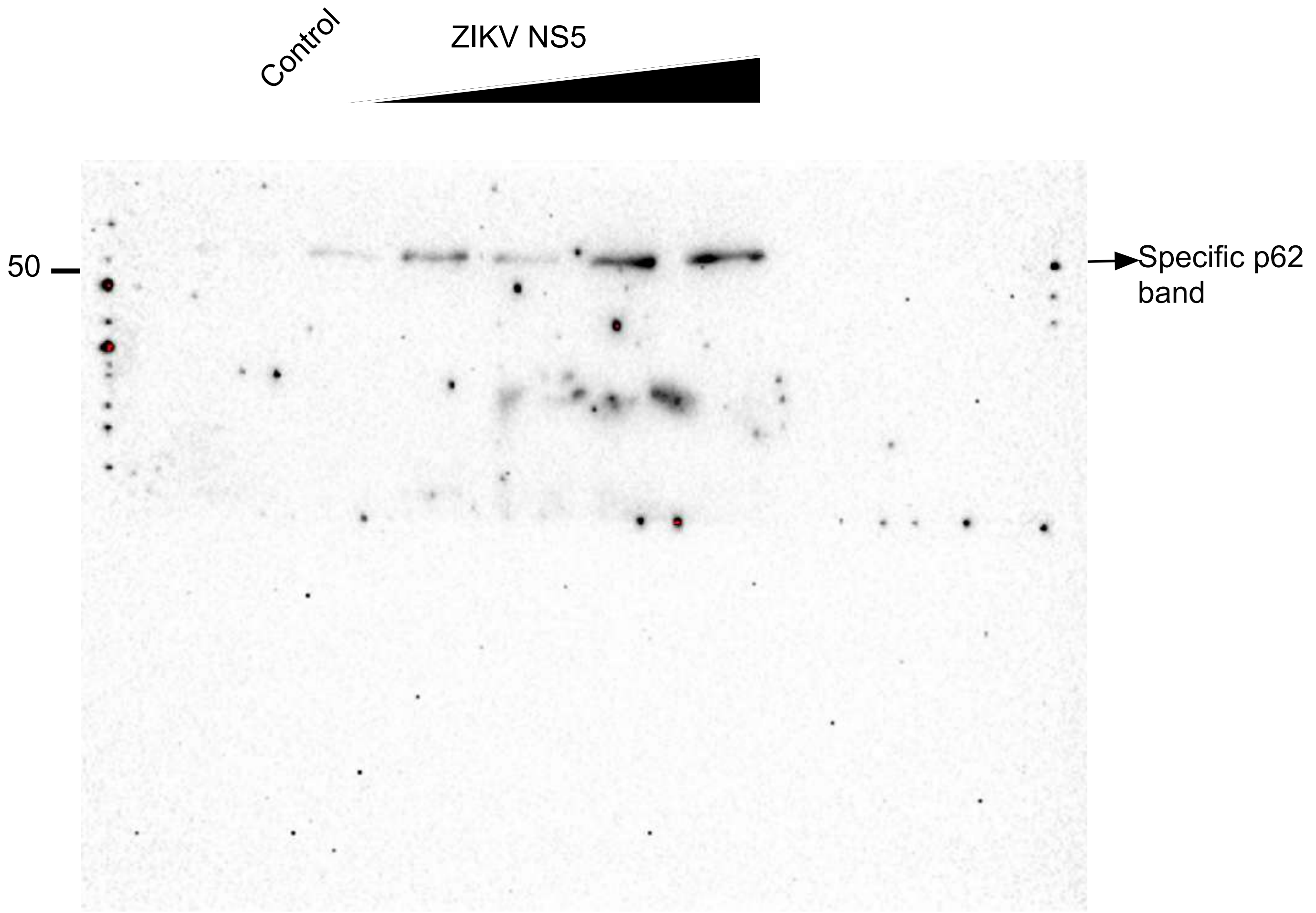

Figure S2E. *Replicate 3 Total α-Tubulin complete gel*

Western-blot associated with Figure S2E
Pérez-Yanes, S., et al.

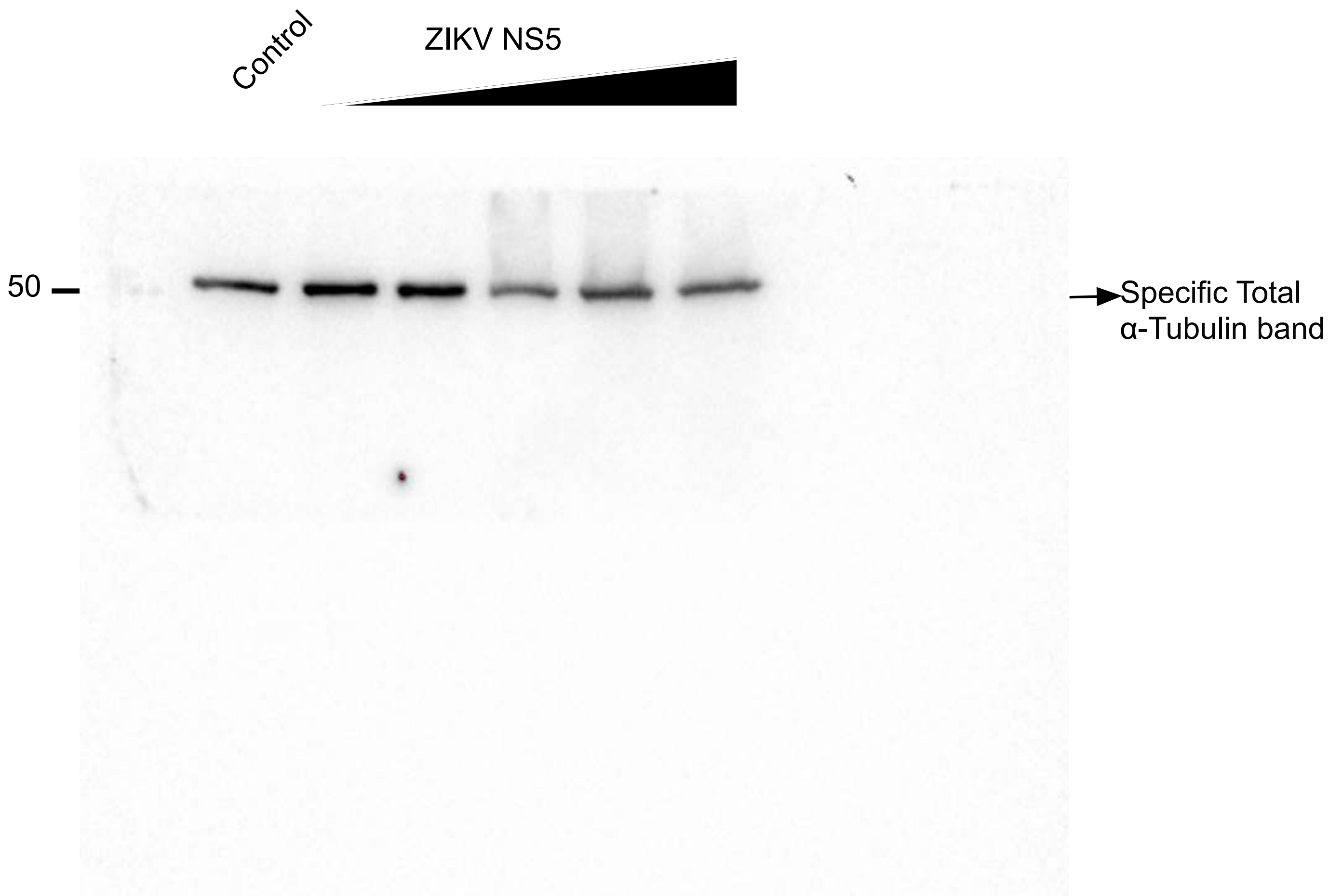

Figura S3A. Western-blot replicates associated with Figure 3A.

Replicate 1 as figure format

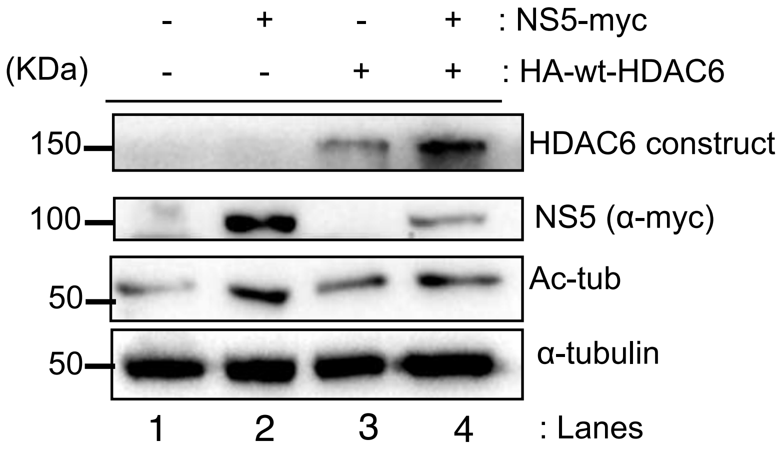

Replicate 2 as figure format

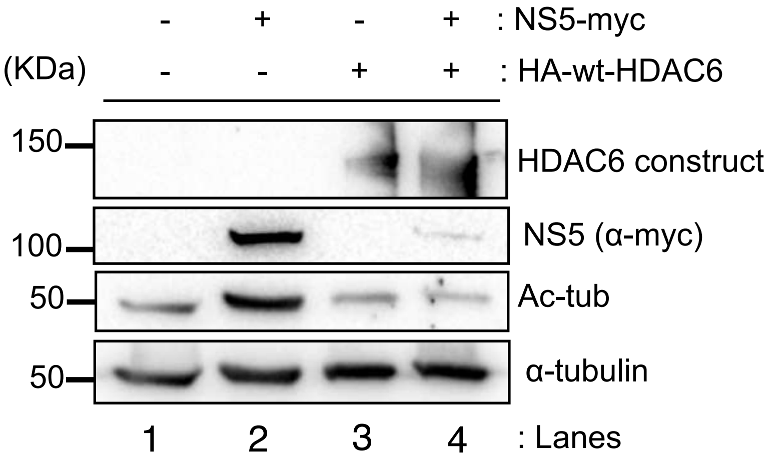

Replicate 3 as figure format

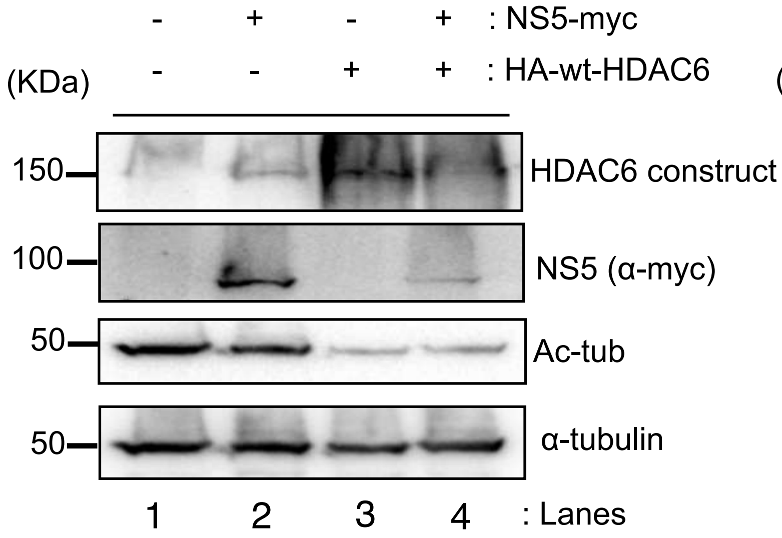

Replicate 4 as figure format

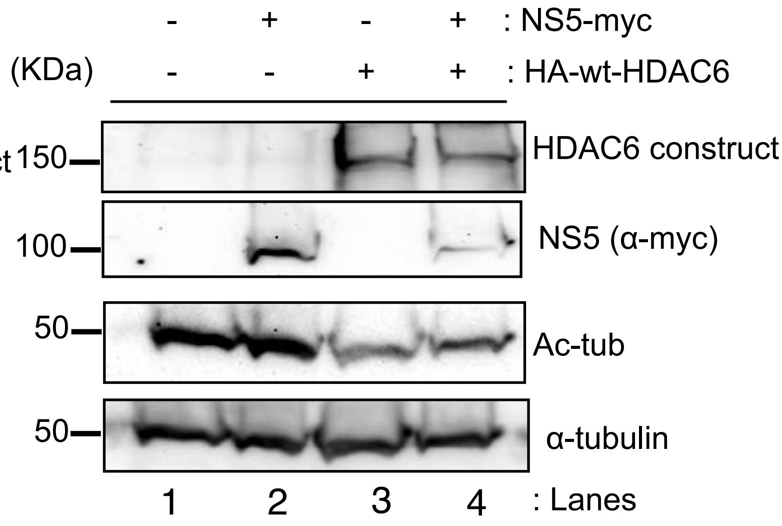

Figure S3A. *Replicate 1 HDAC6 complete gel*

Western-blot associated with Figure 3A
Pérez-Yanes, S., et al.

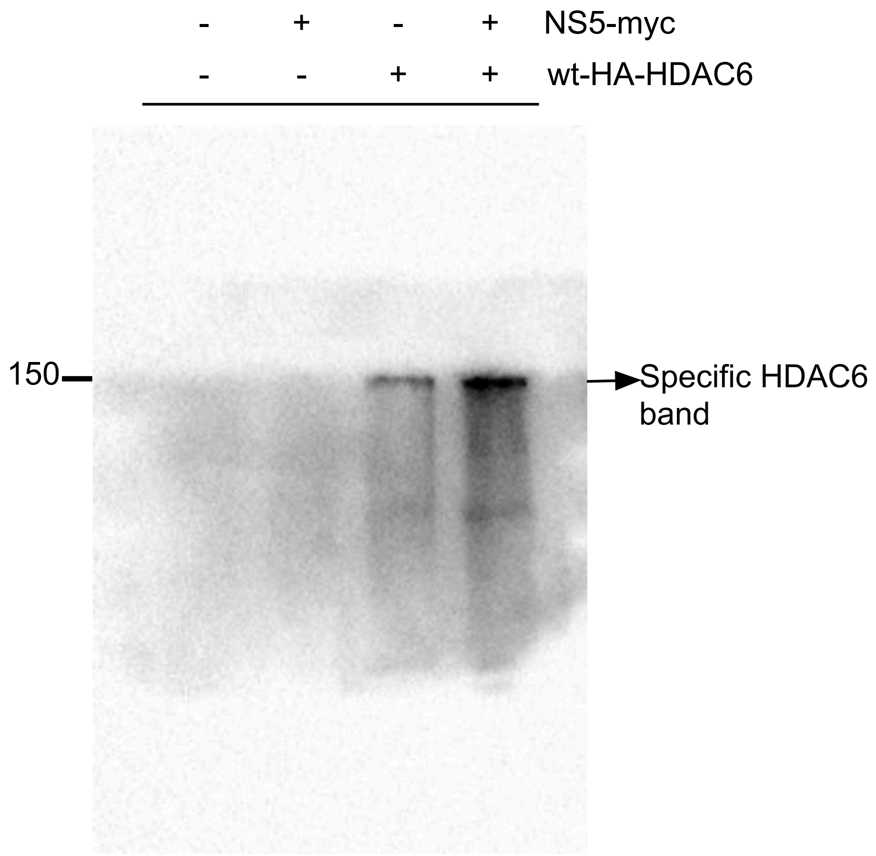

Figure S3A. *Replicate 1 myc complete gel*

Western-blot associated with Figure 3A
Pérez-Yanes, S., et al.

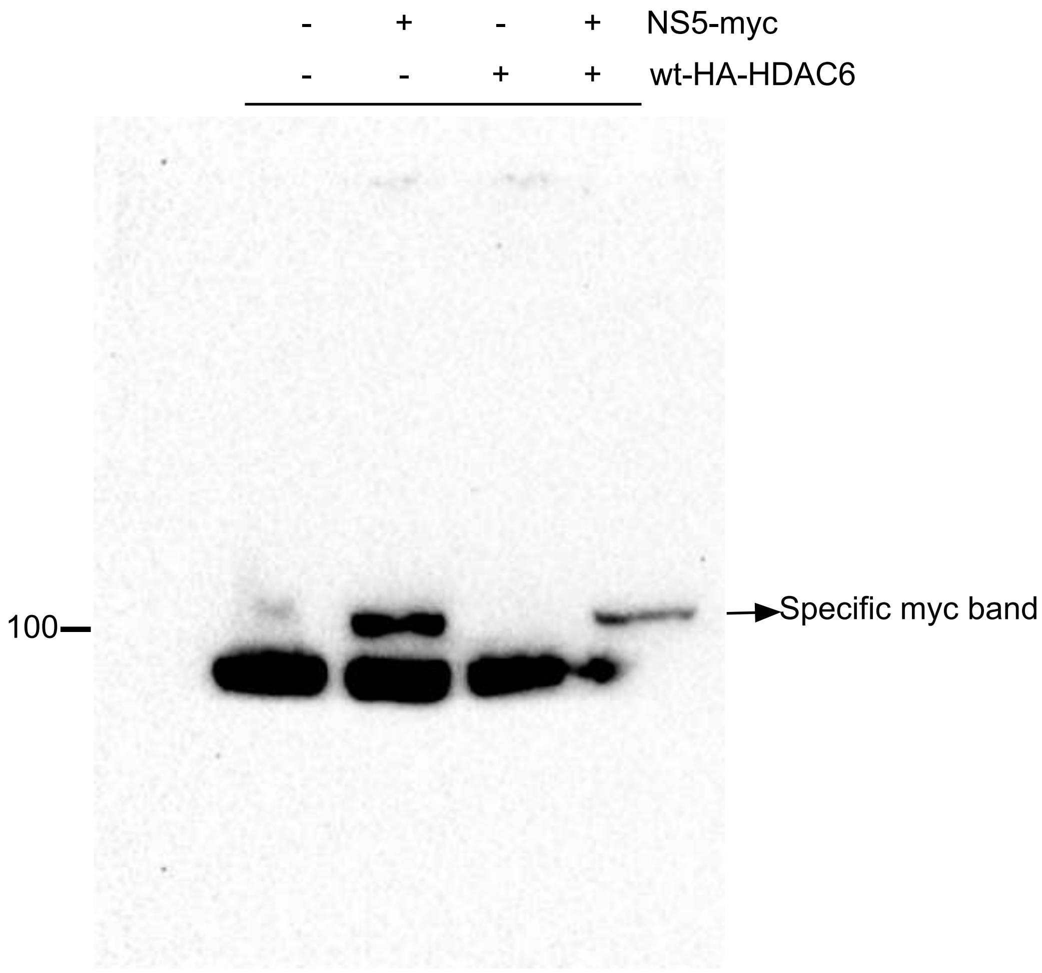

Figure S3A. *Replicate 1 Total α-Tubulin complete gel*

Western-blot associated with Figure 3A
Pérez-Yanes, S., et al.

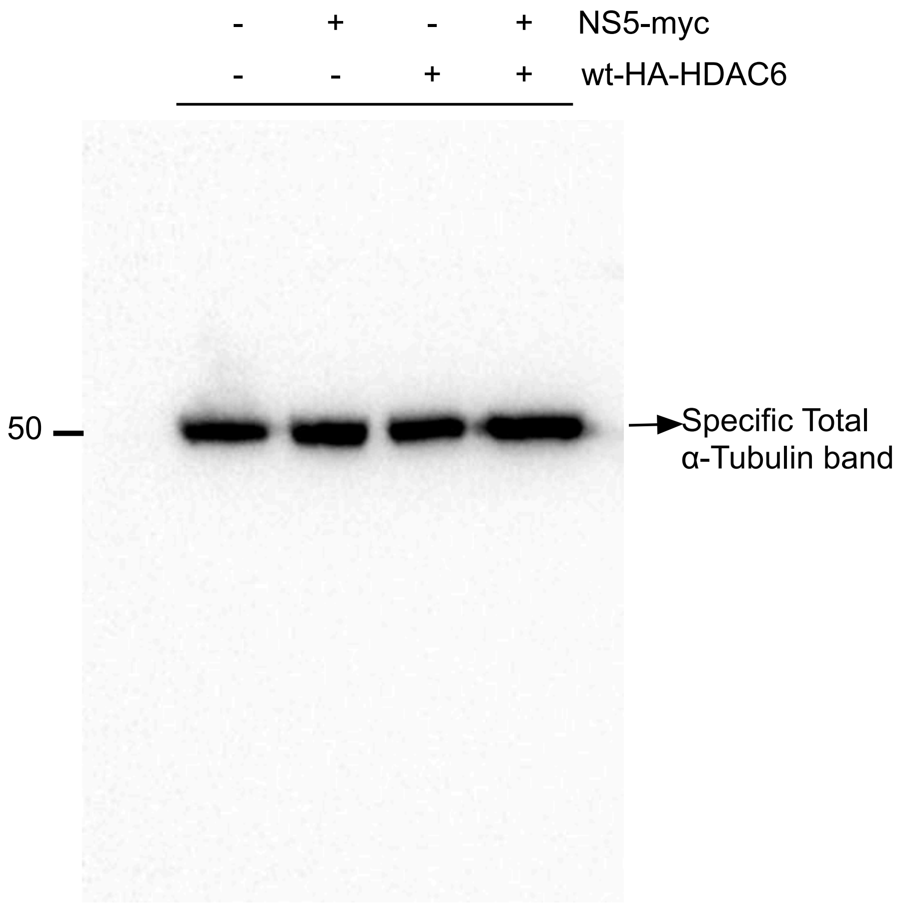

Figure S3A. *Replicate 1 Acetylated α-Tubulin complete gel*

Western-blot associated with Figure 3A
Pérez-Yanes, S., et al.

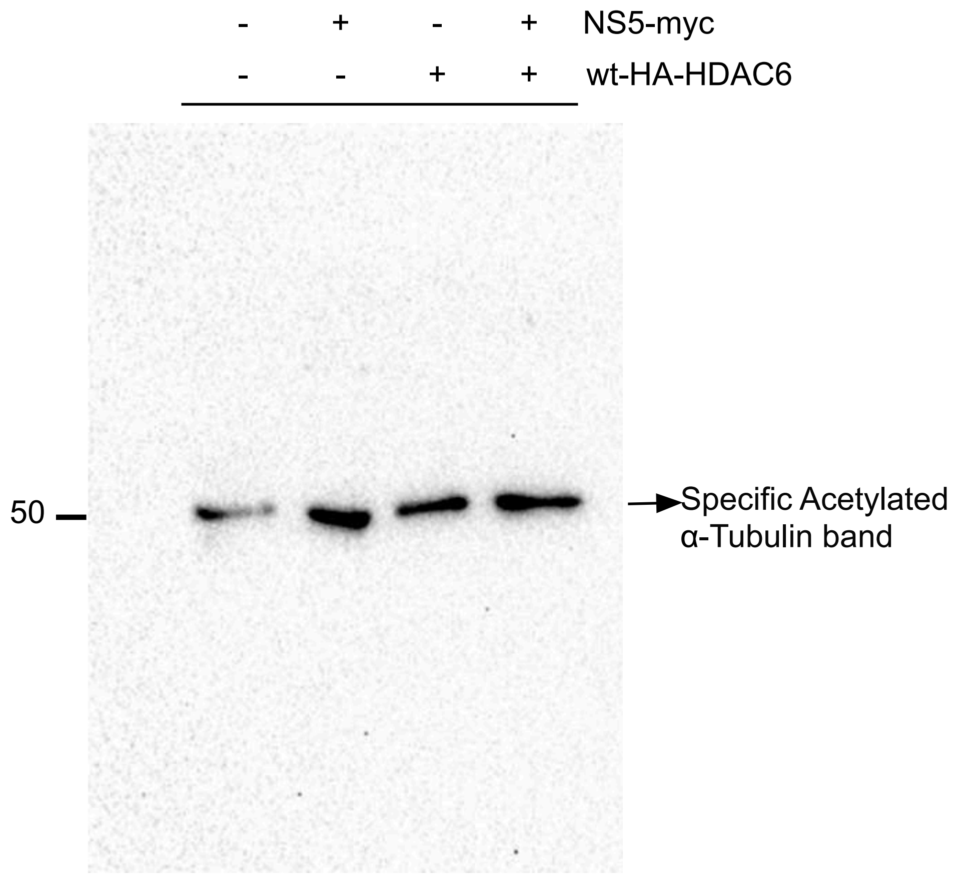

Figure S3A. *Replicate 2 HDAC6 complete gel*

Western-blot associated with Figure 3A
Pérez-Yanes, S., et al.

-	+	-	+	NS5-myc
-	-	+	+	wt-HA-HDAC6

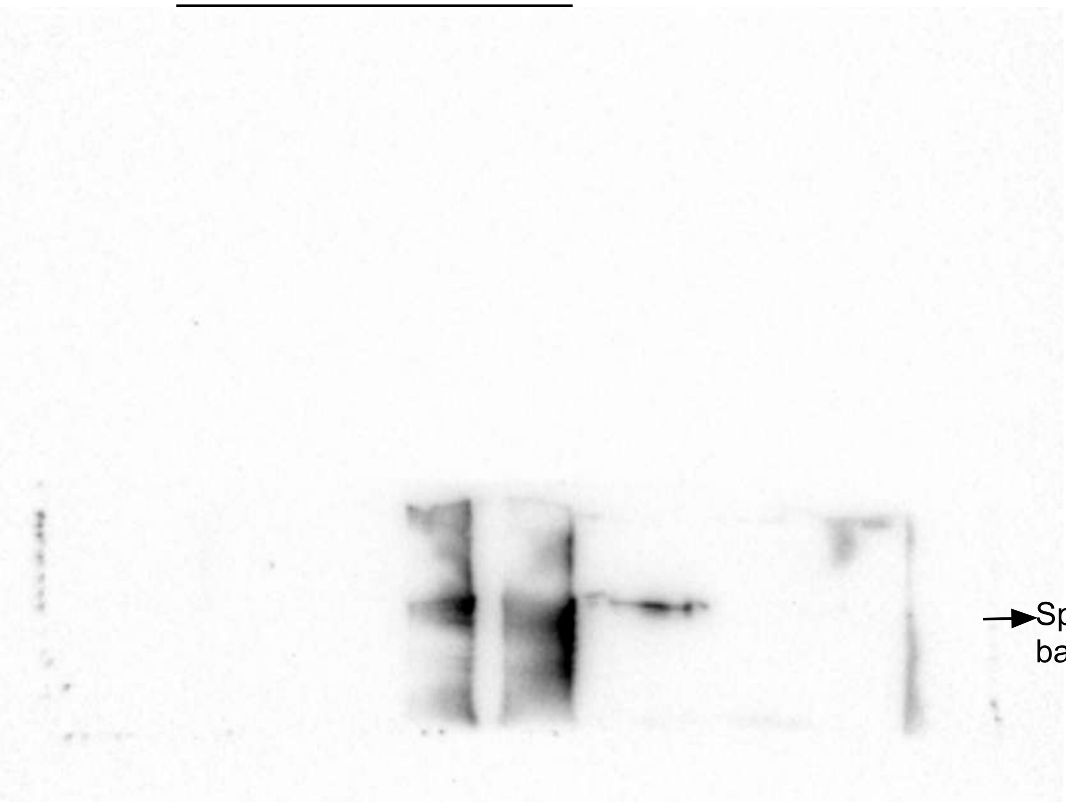

150

Specific HDAC6 band

Figure S3A. *Replicate 2 myc complete gel*

Western-blot associated with Figure 3A
Pérez-Yanes, S., et al.

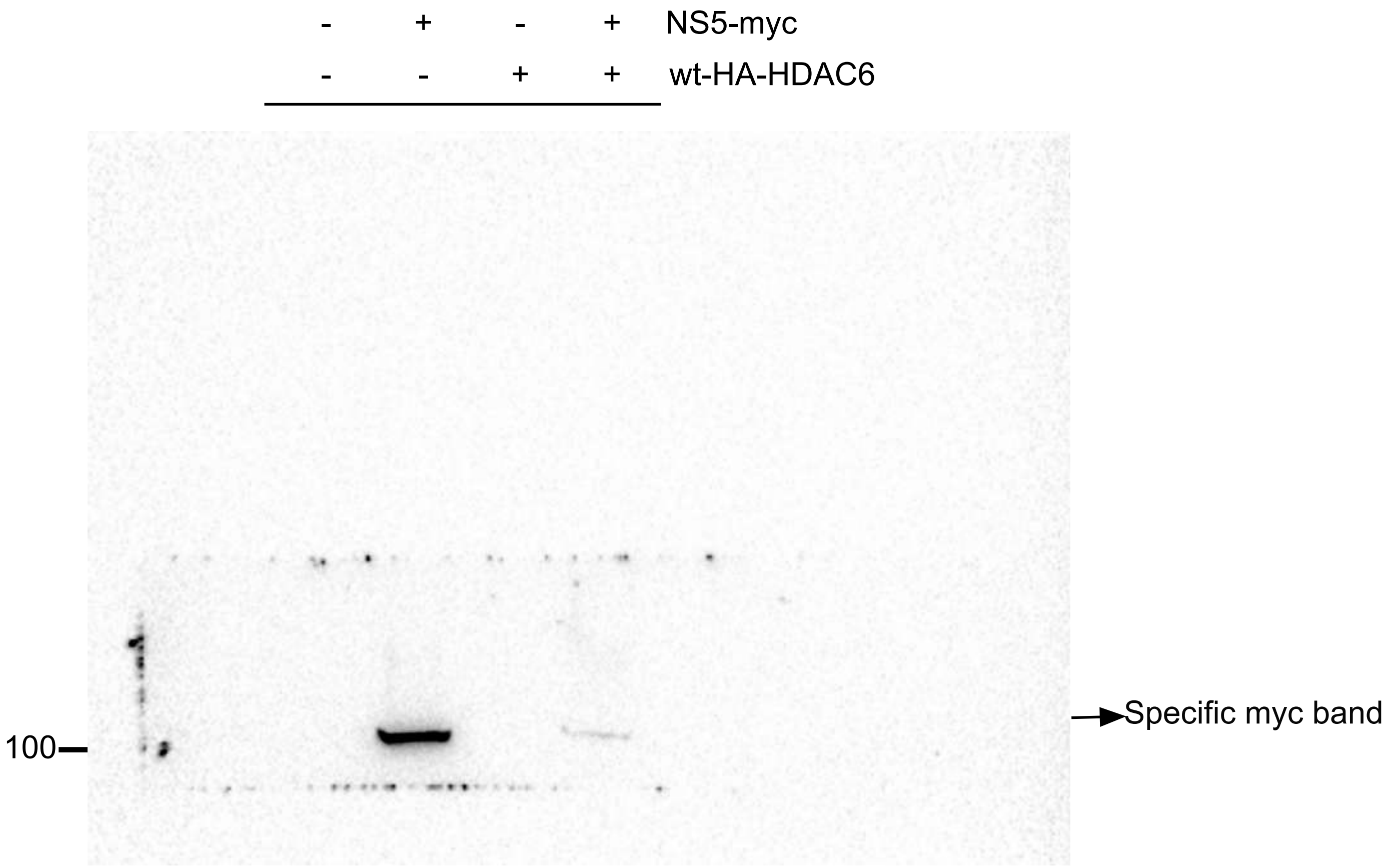

Figure S3A. *Replicate 2 Total α-Tubulin complete gel*

Western-blot associated with Figure 3A
Pérez-Yanes, S., et al.

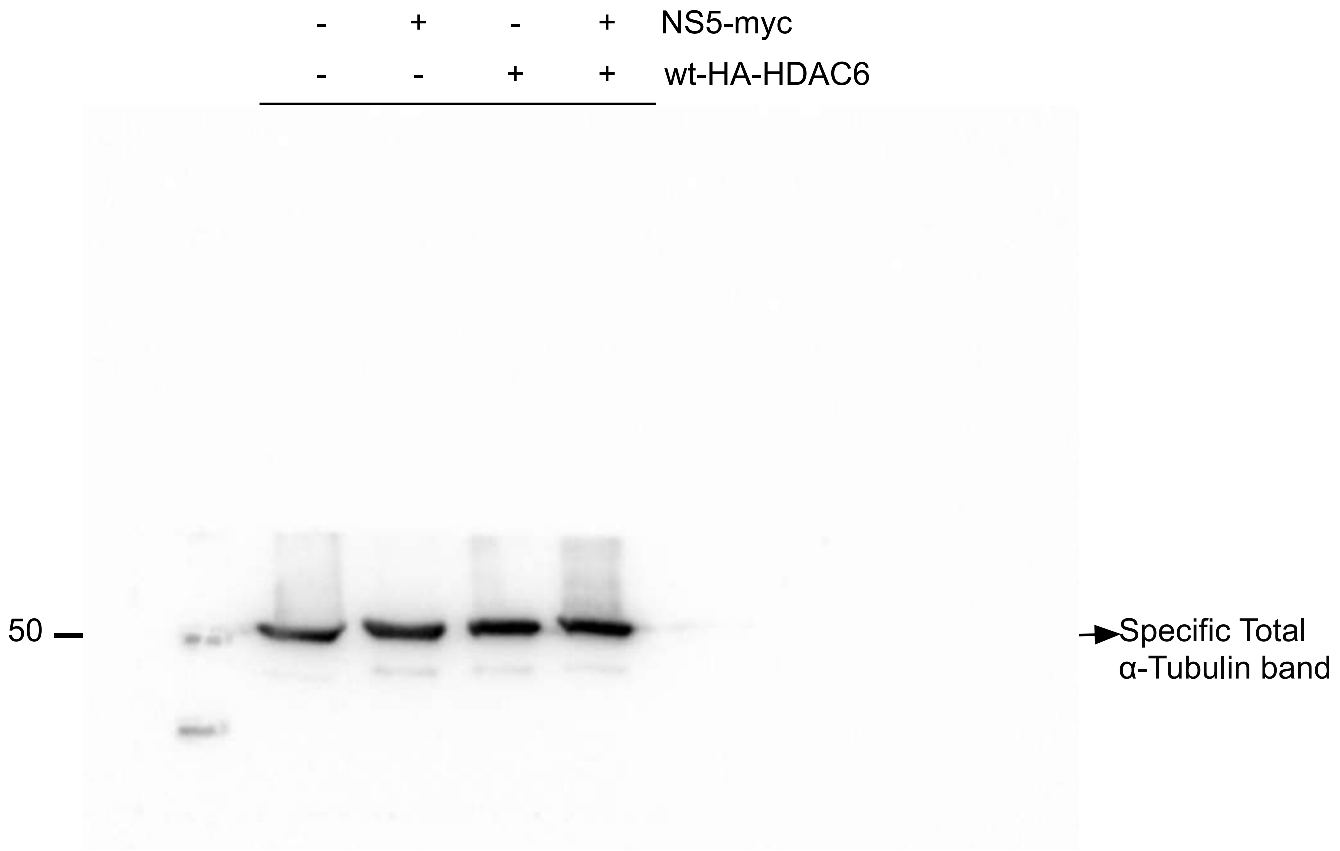

Figure S3A. *Replicate 2 Acetylated α-Tubulin complete gel*

Western-blot associated with Figure 3A
Pérez-Yanes, S., et al.

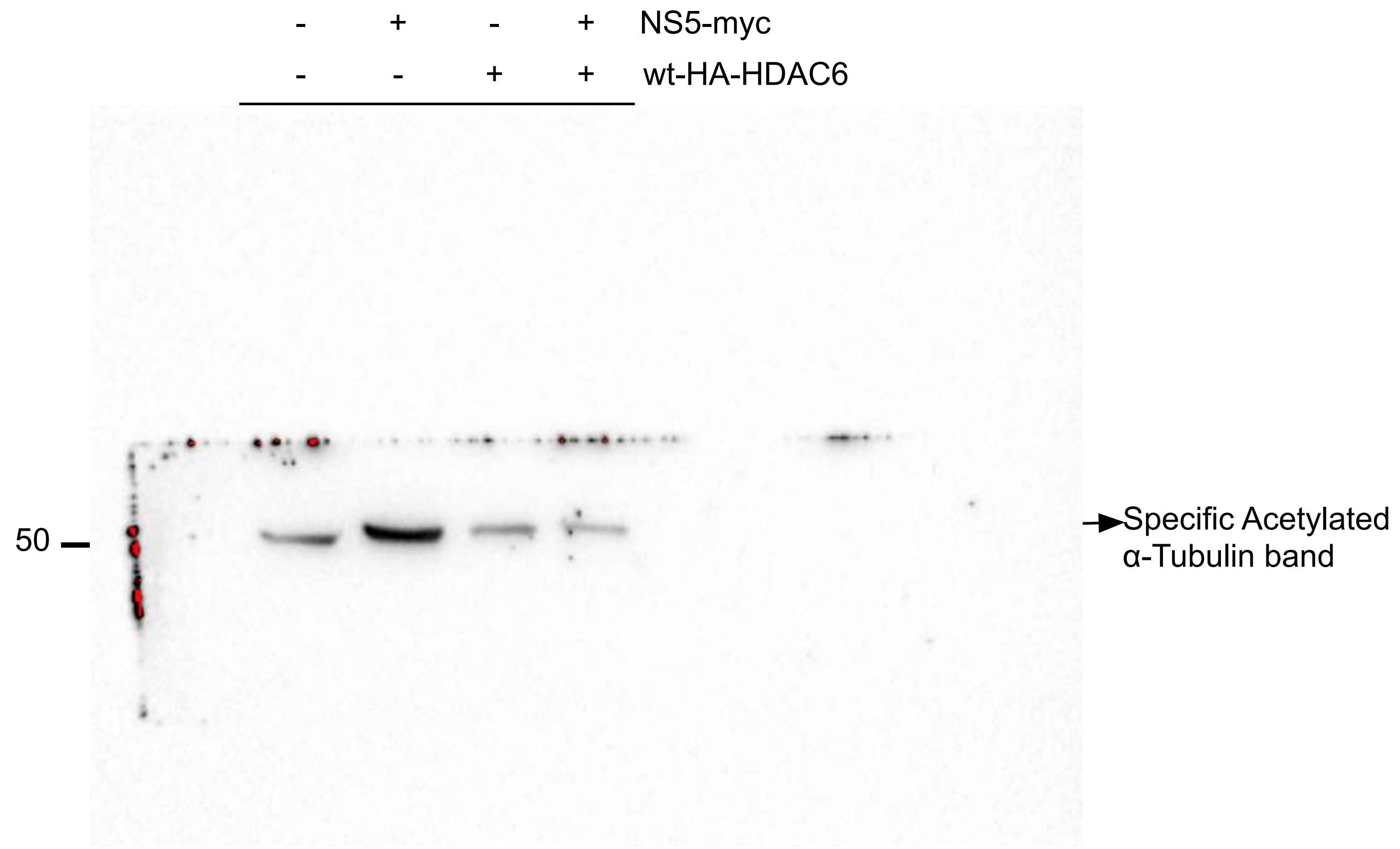

Figure S3A. *Replicate 3 HDAC6 complete gel*

Western-blot associated with Figure 3A
Pérez-Yanes, S., et al.

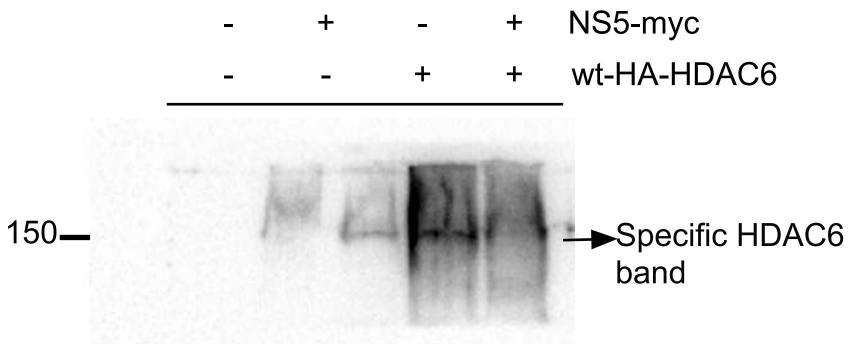

Figure S3A. *Replicate 3 myc complete gel*

Western-blot associated with Figure 3A
Pérez-Yanes, S., et al.

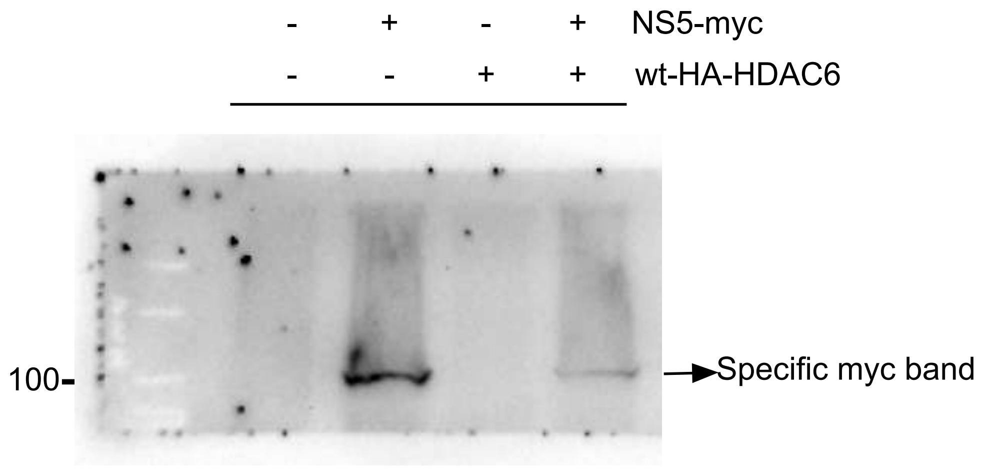

Figure S3A. *Replicate 3 Total* α*-Tubulin complete gel*

Western-blot associated with Figure 3A
Pérez-Yanes, S., et al.

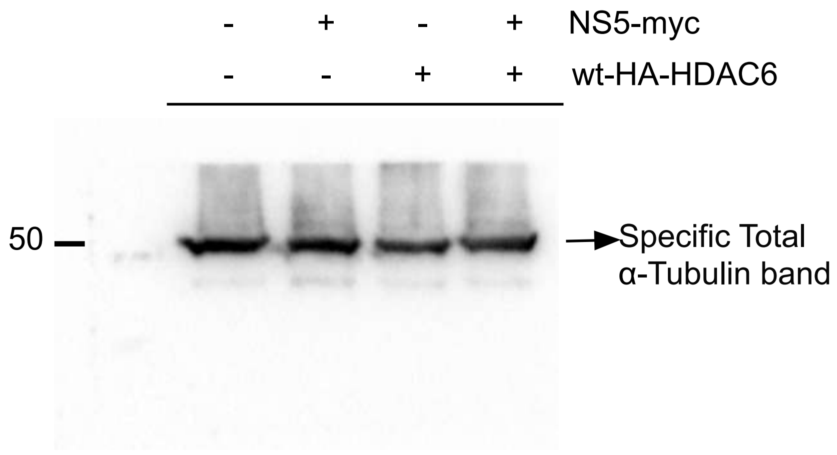

Figure S3A. *Replicate 3 Acetylated α-Tubulin complete gel*

Western-blot associated with Figure 3A
Pérez-Yanes, S., et al.

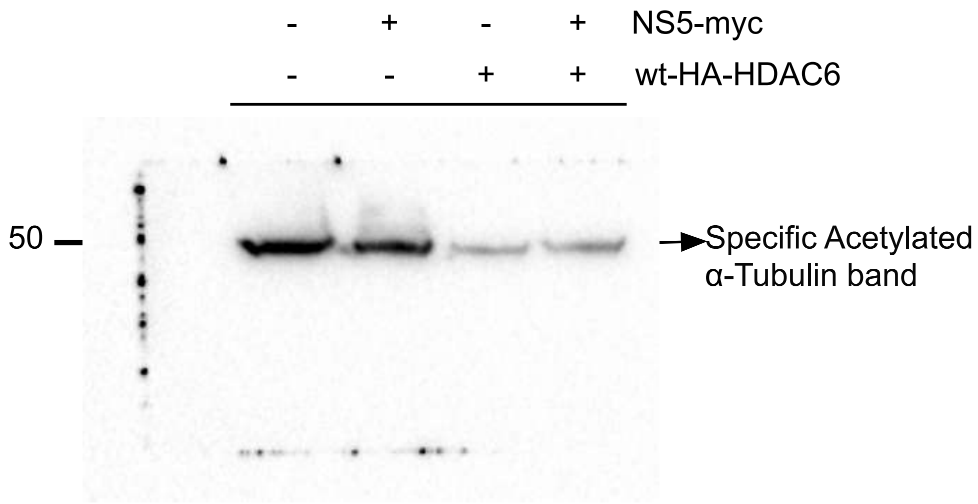

Figure S3A. *Replicate 4 HDAC6 complete gel*

Western-blot associated with Figure 3A
Pérez-Yanes, S., et al.

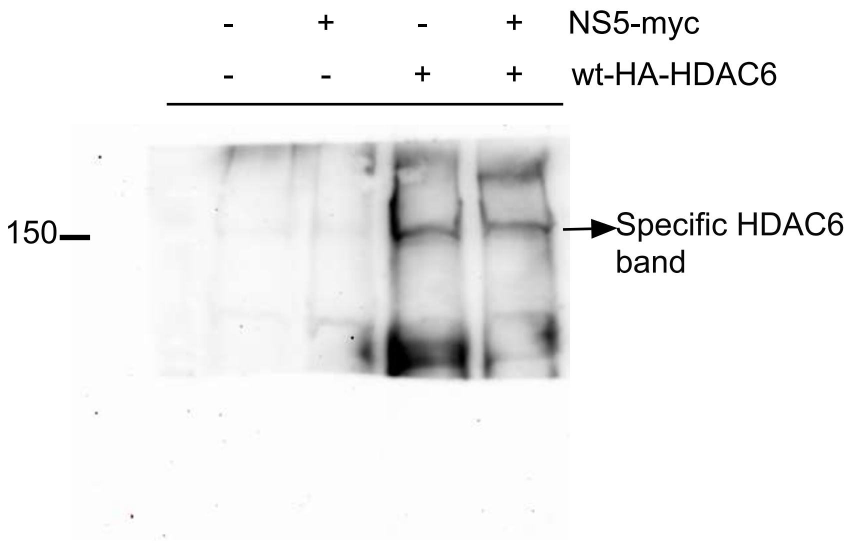

Figure S3A. *Replicate 4 myc complete gel*

Western-blot associated with Figure 3A
Pérez-Yanes, S., et al.

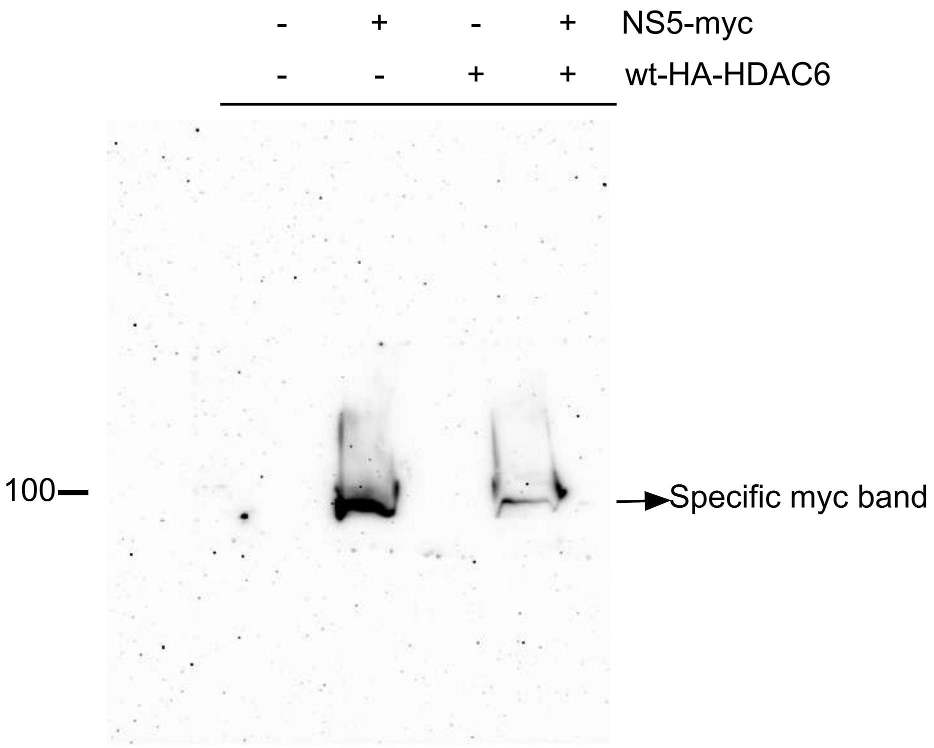

Figure S3A. *Replicate 4 Total α-Tubulin complete gel*

Western-blot associated with Figure 3A
Pérez-Yanes, S., et al.

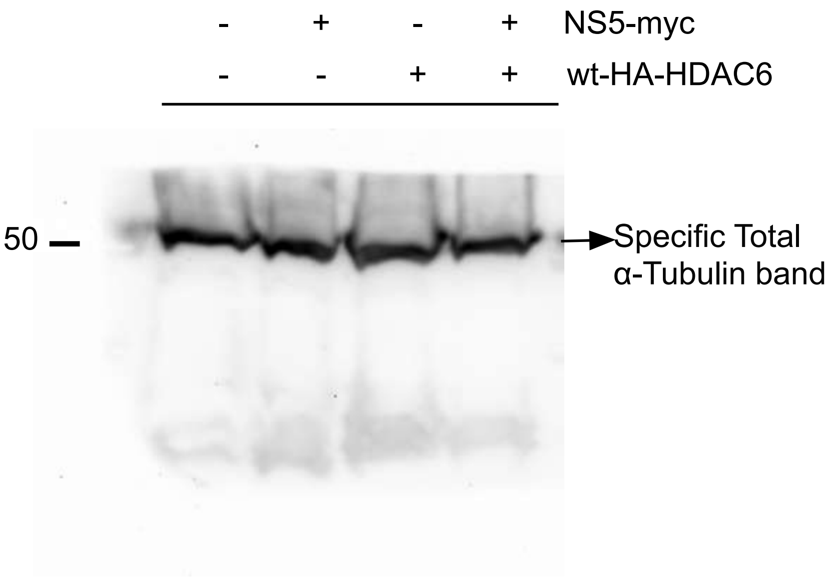

Figure S3A. *Replicate 4 Acetylated α-Tubulin complete gel*

Western-blot associated with Figure 3A
Pérez-Yanes, S., et al.

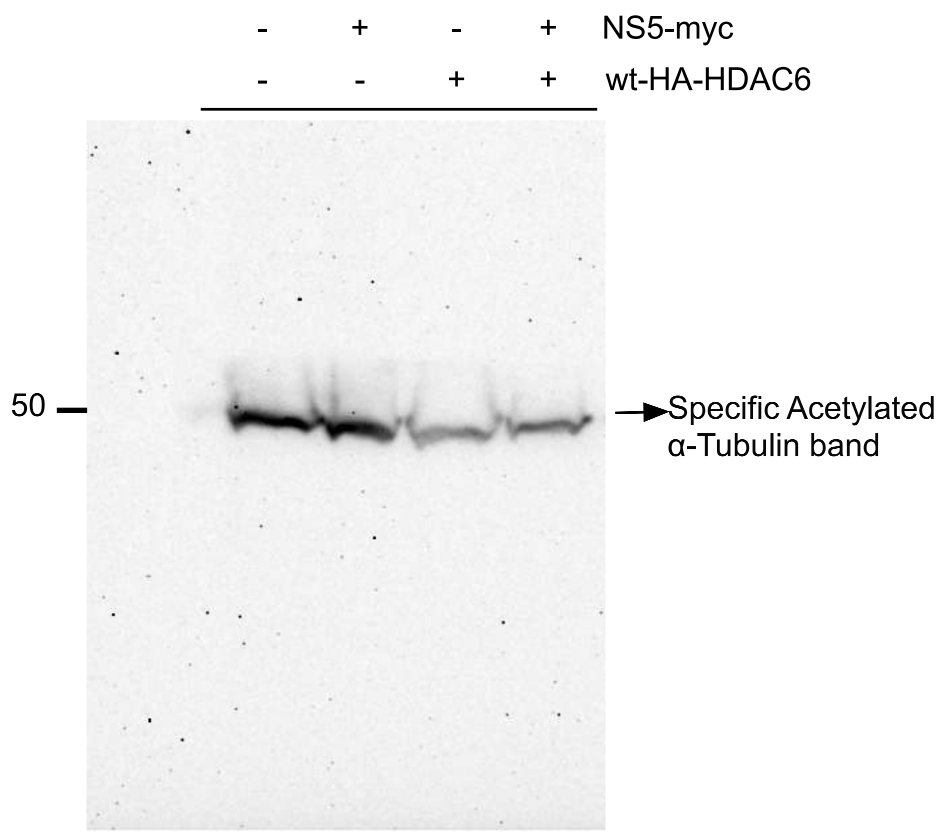

Figura S3D. Western-blot replicates associated with Figure 3D.

Replicate 1 as figure format

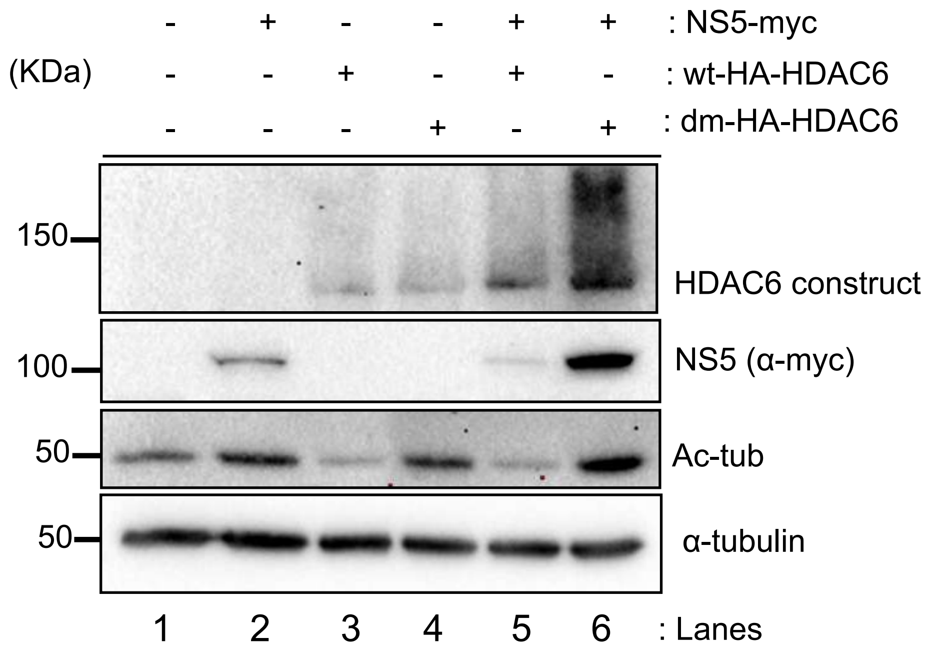

Replicate 2 as figure format

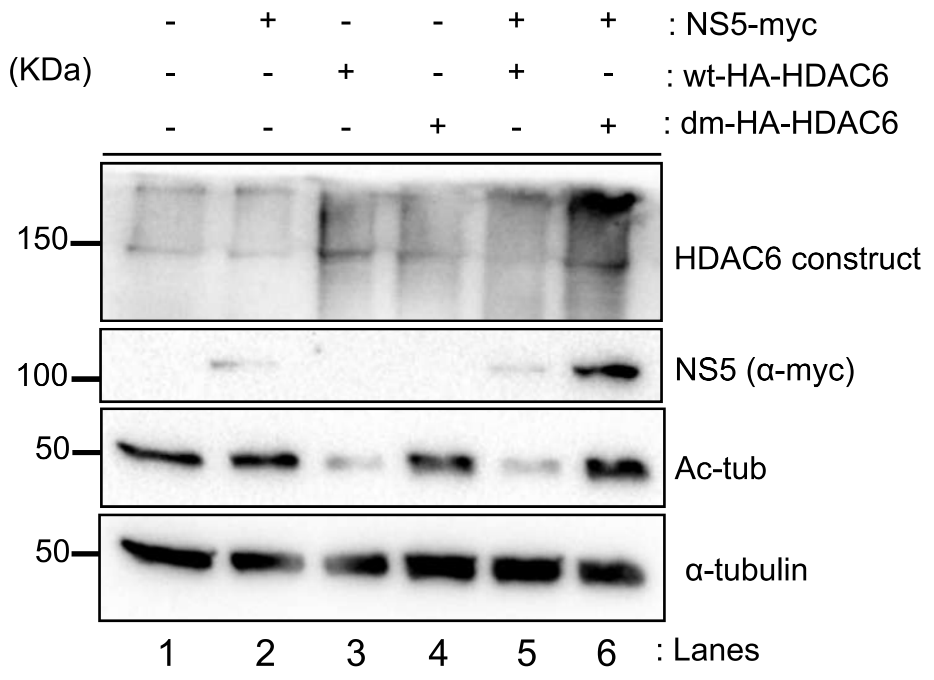

Figure S3D. *Replicate 1 HDAC6 complete gel*

Western-blot associated with Figure 3D
Pérez-Yanes, S., et al.

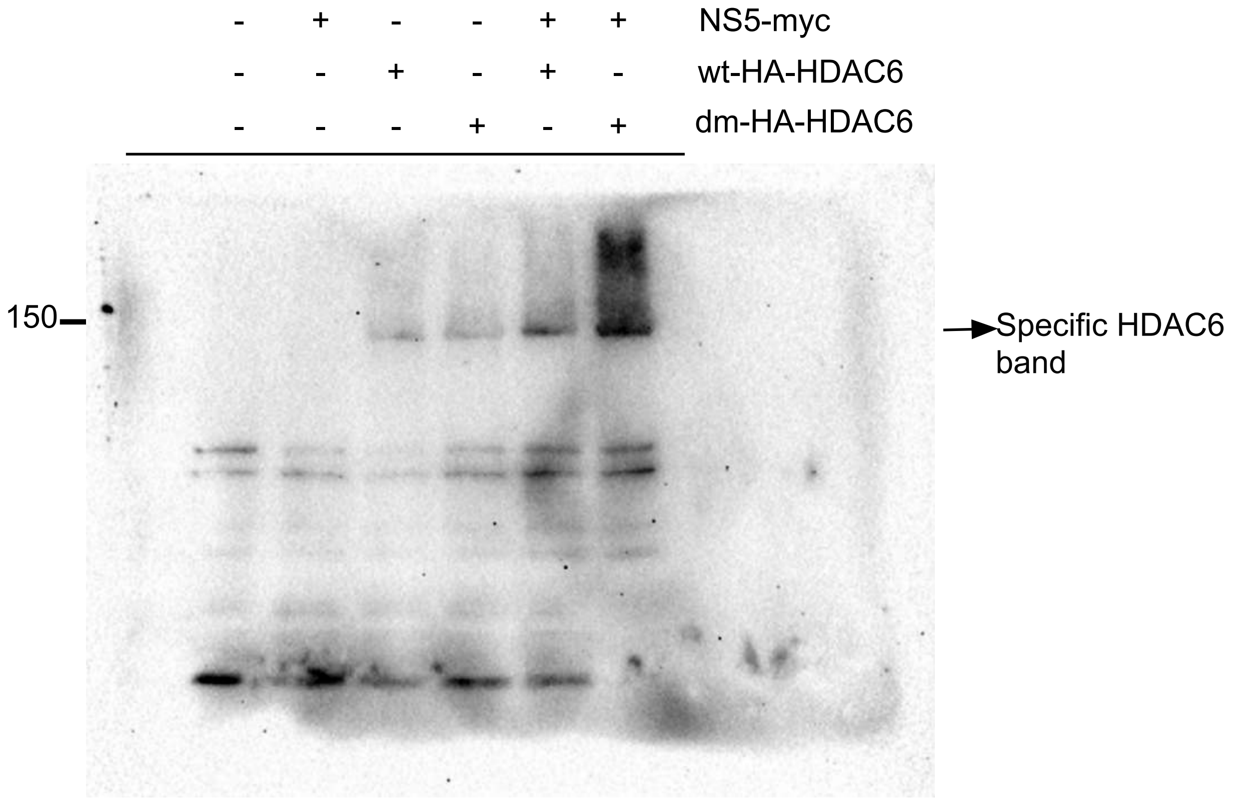

Figure S3D. *Replicate 1 myc complete gel*

Western-blot associated with Figure 3D
Pérez-Yanes, S., et al.

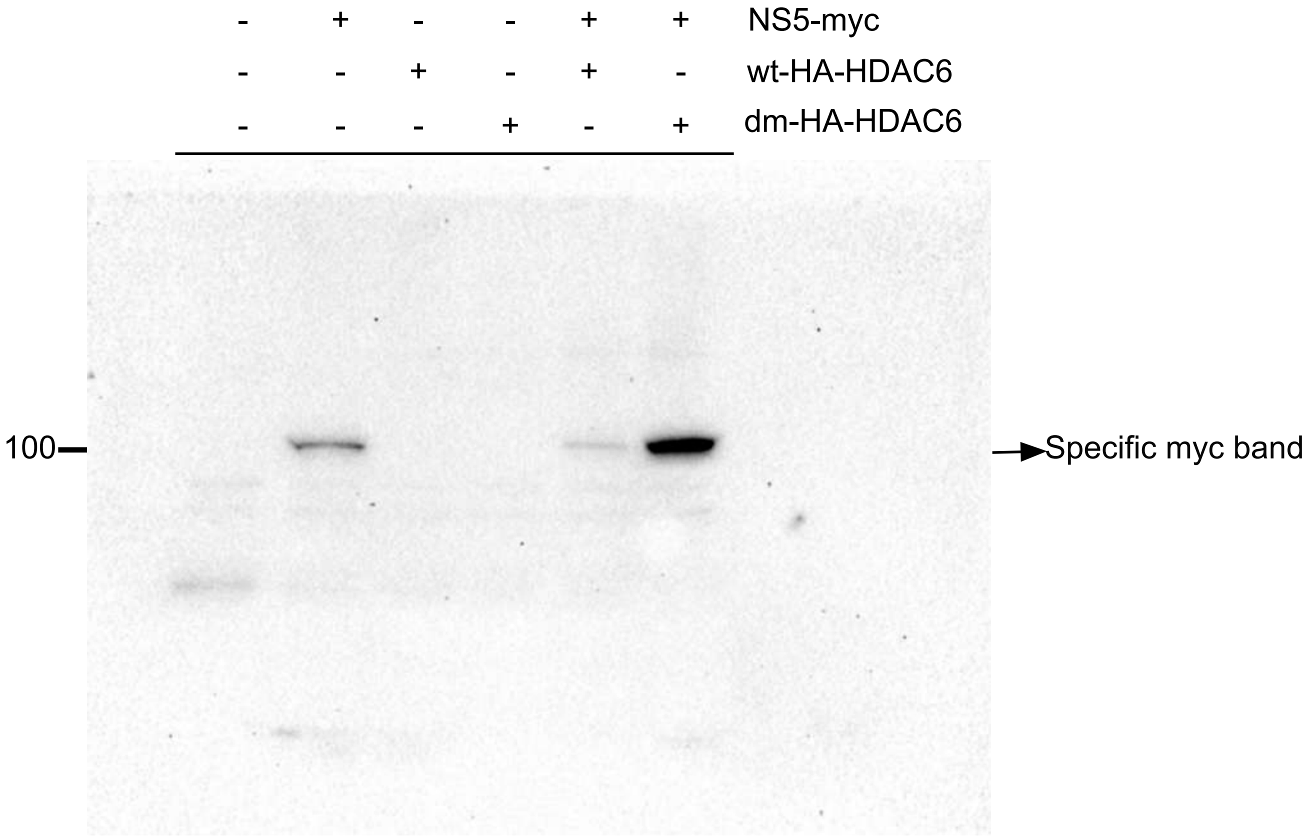

Figure S3D. *Replicate 1 Total α-Tubulin complete gel*

Western-blot associated with Figure 3D
Pérez-Yanes, S., et al.

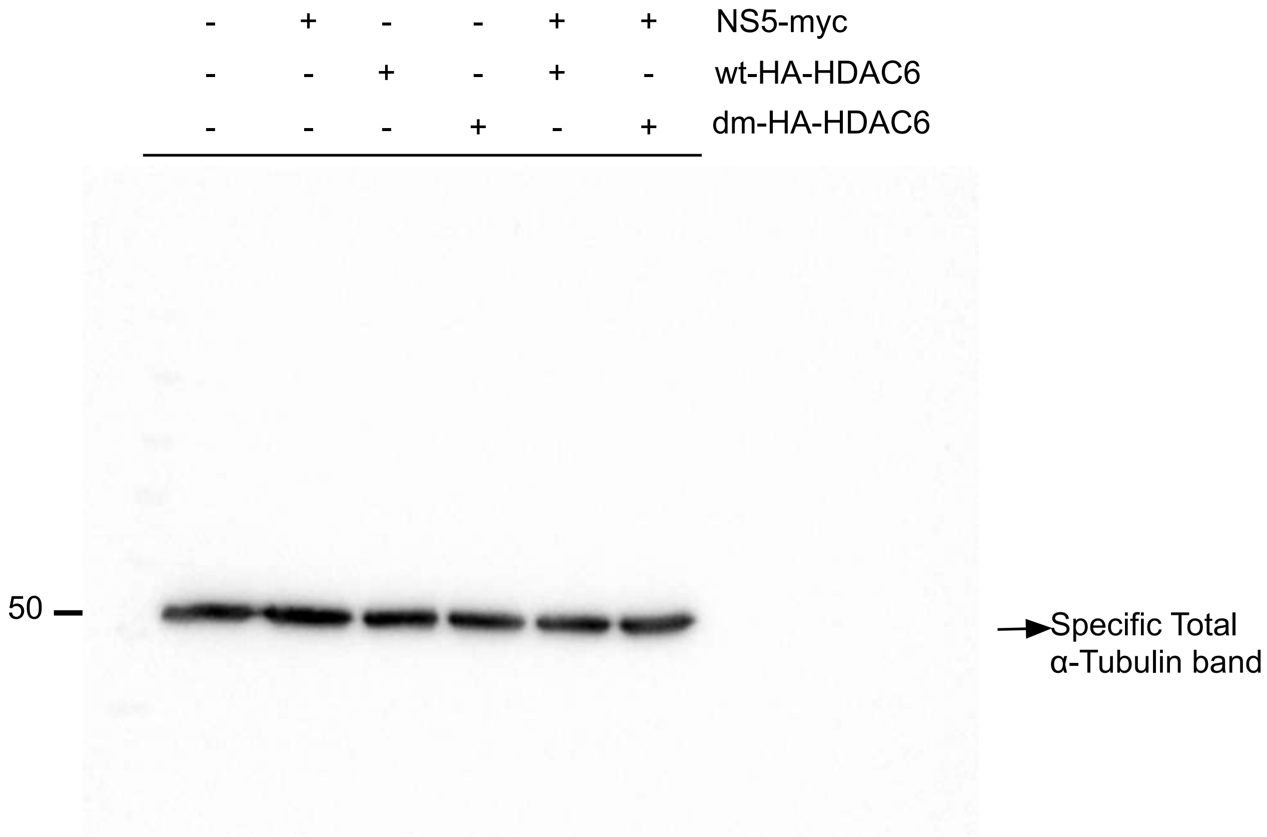

Figure S3D. *Replicate 1 Acetylated* α*-Tubulin complete gel*

Western-blot associated with Figure 3D
Pérez-Yanes, S., et al.

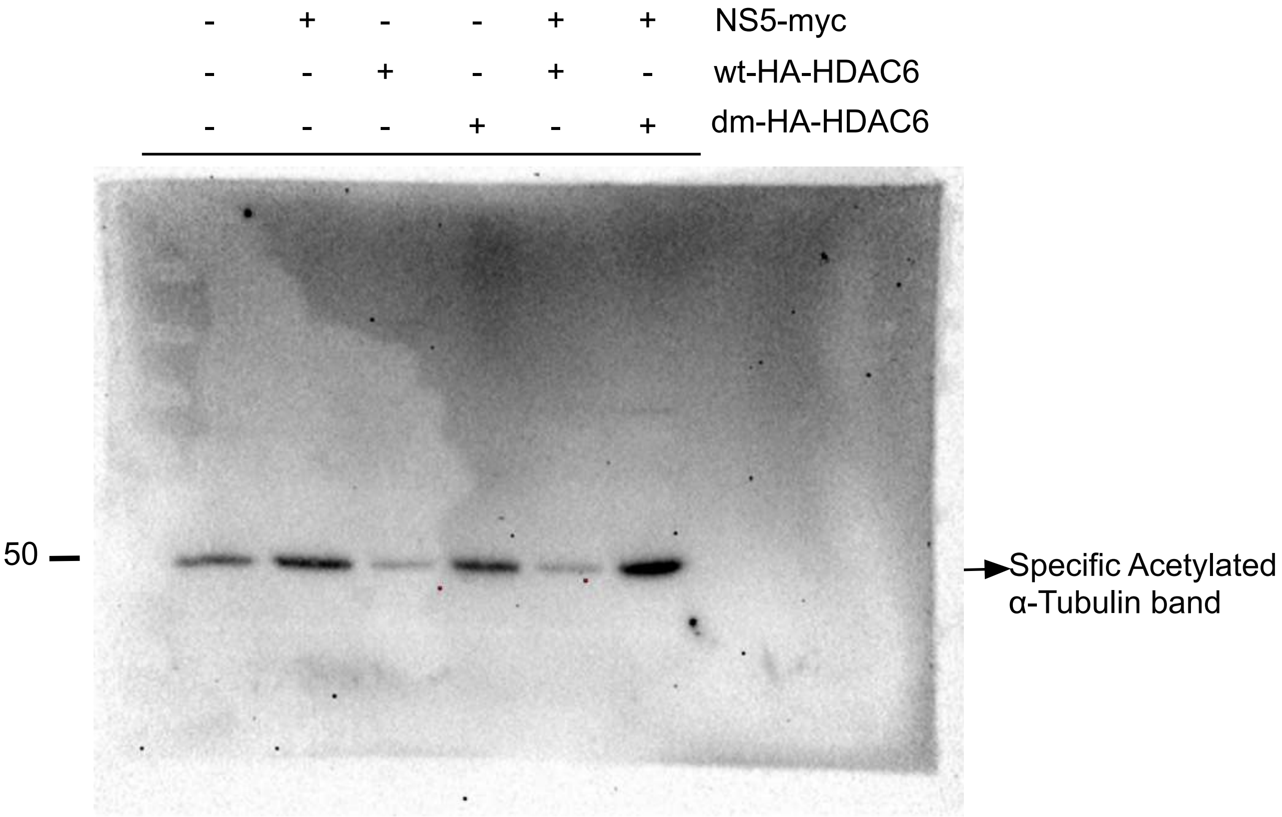

Figure S3D. *Replicate 2 HDAC6 complete gel*

Western-blot associated with Figure 3D
Pérez-Yanes, S., et al.

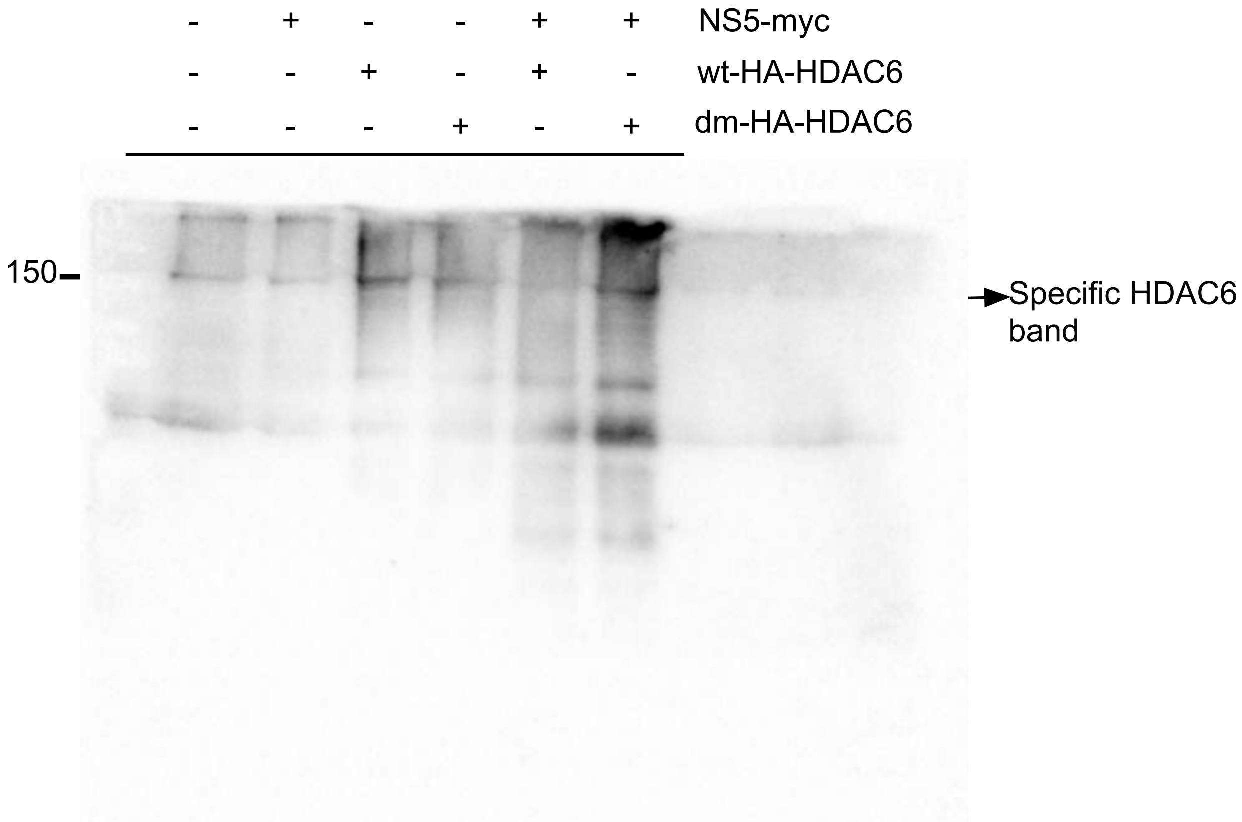

Figure S3D. *Replicate 2 myc complete gel*

Western-blot associated with Figure 3D
Pérez-Yanes, S., et al.

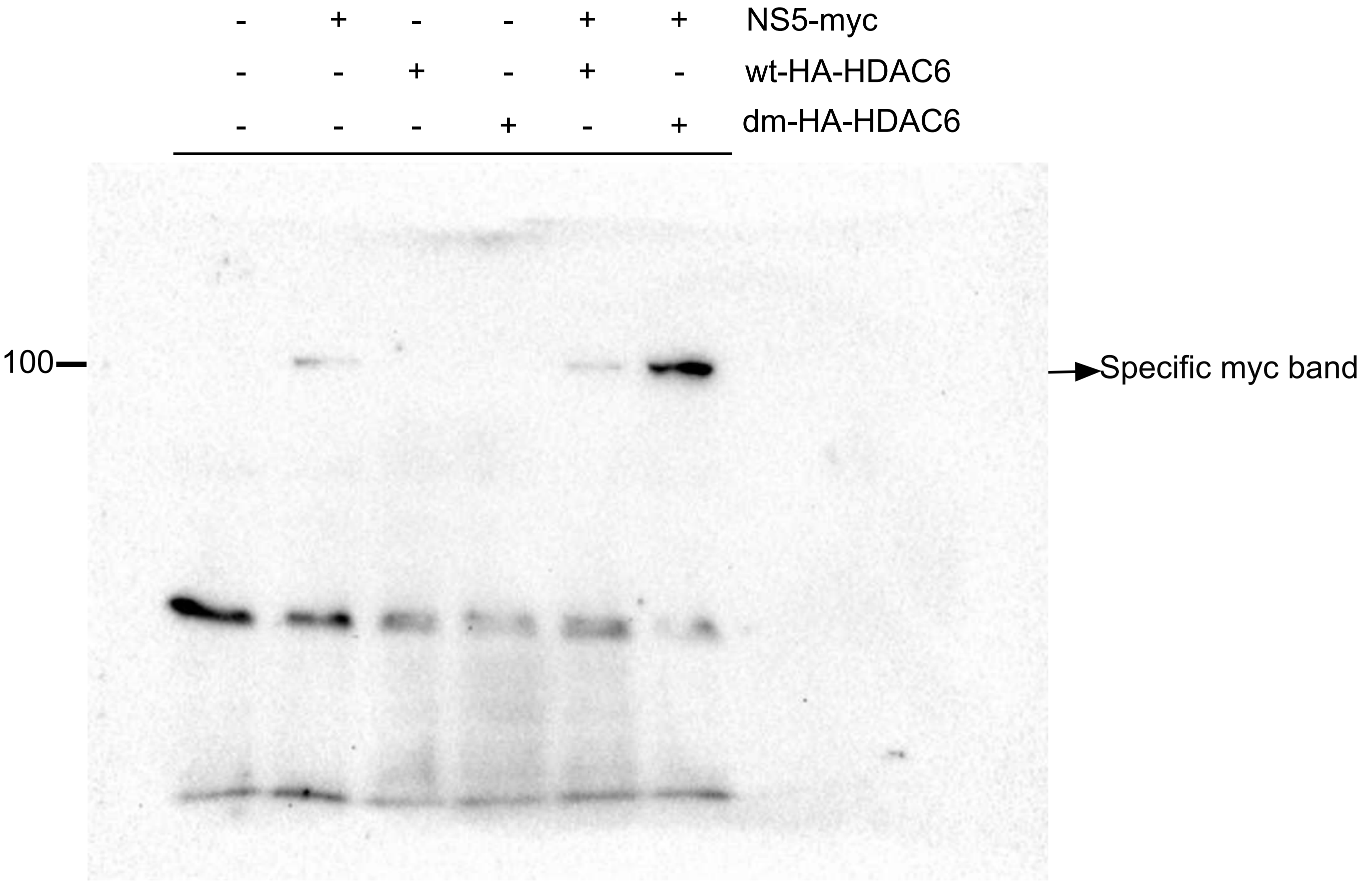

Figure S3D. *Replicate 2 Total α-Tubulin complete gel*

Western-blot associated with Figure 3D
Pérez-Yanes, S., et al.

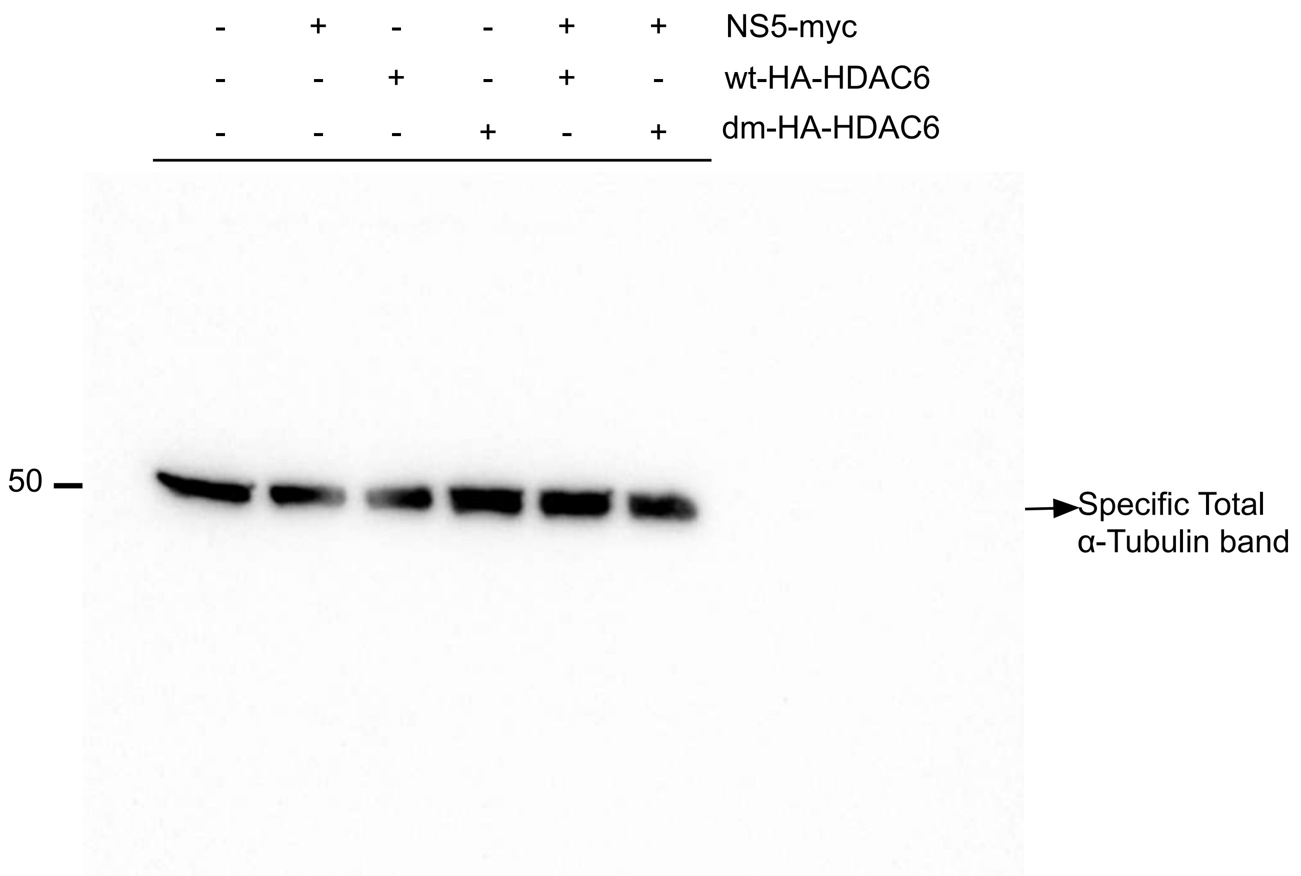

Figure S3D. *Replicate 2 Acetylated* α*-Tubulin complete gel*

Western-blot associated with Figure 3D
Pérez-Yanes, S., et al.

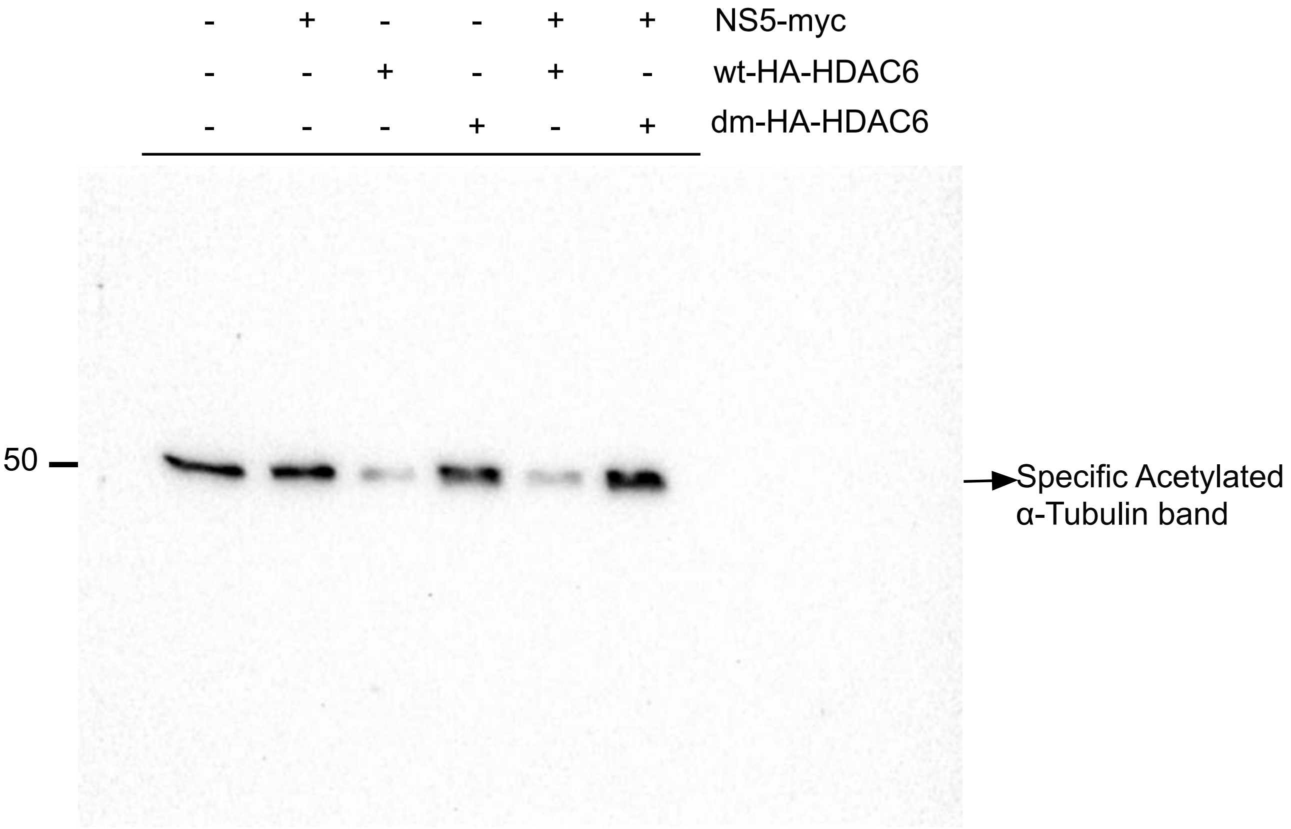

Figura S4A. Western-blot replicates associated with Figure 4A.

Replicate 1 as figure format

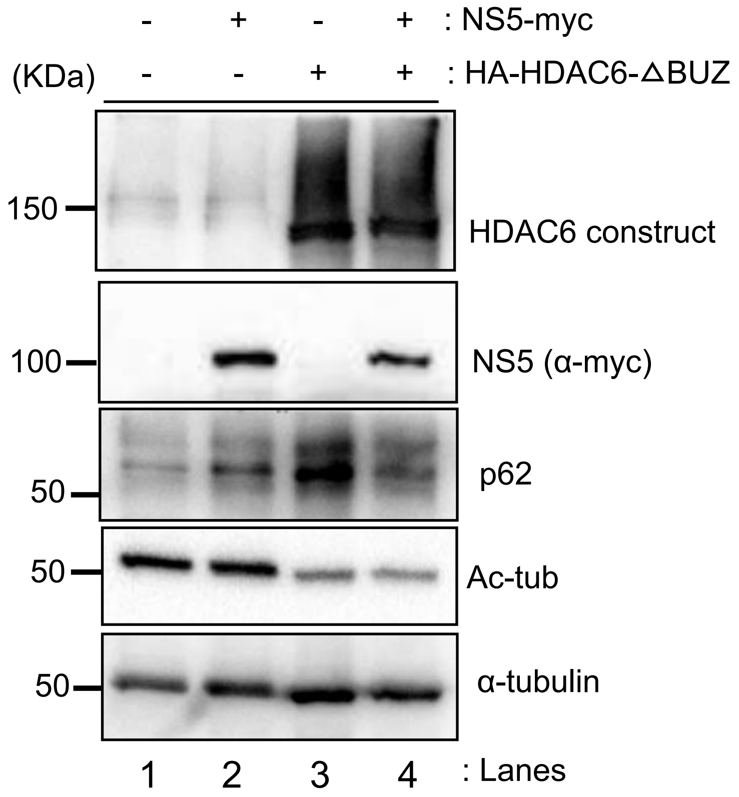

Replicate 2 as figure format

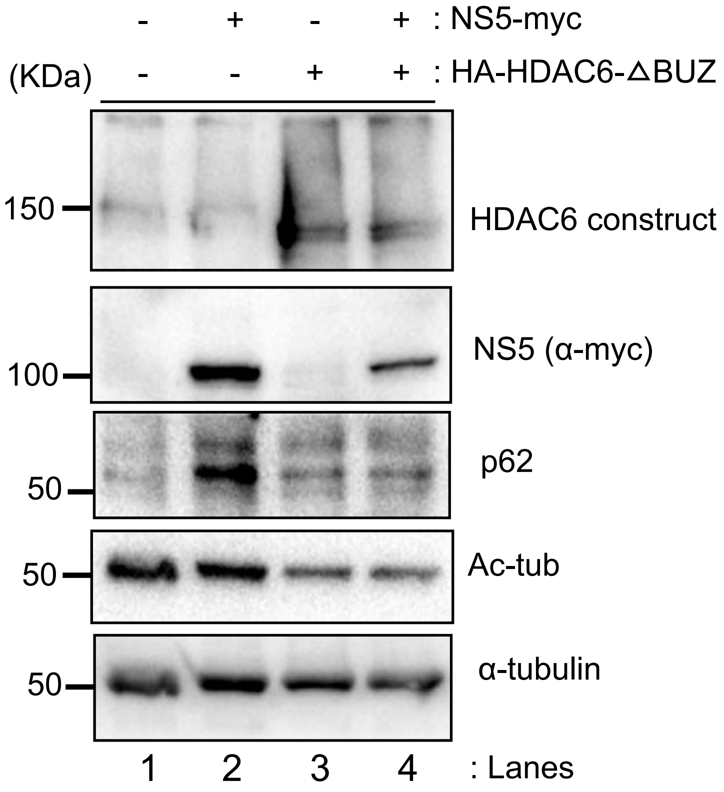

Replicate 3 as figure format

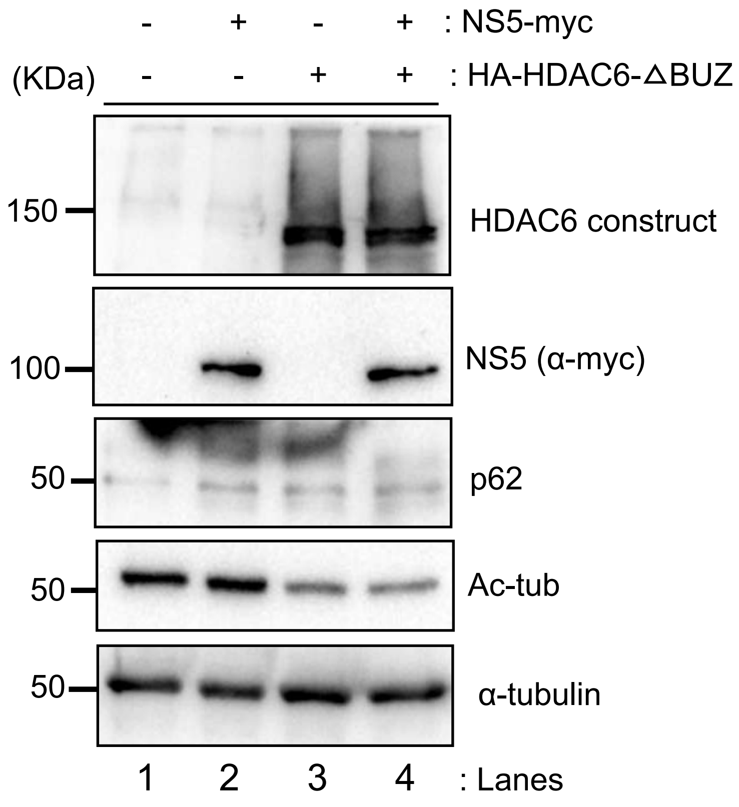

Figure S4A. *Replicate 1 HDAC6 complete gel*

Western-blot associated with Figure 4A
Pérez-Yanes, S., et al.

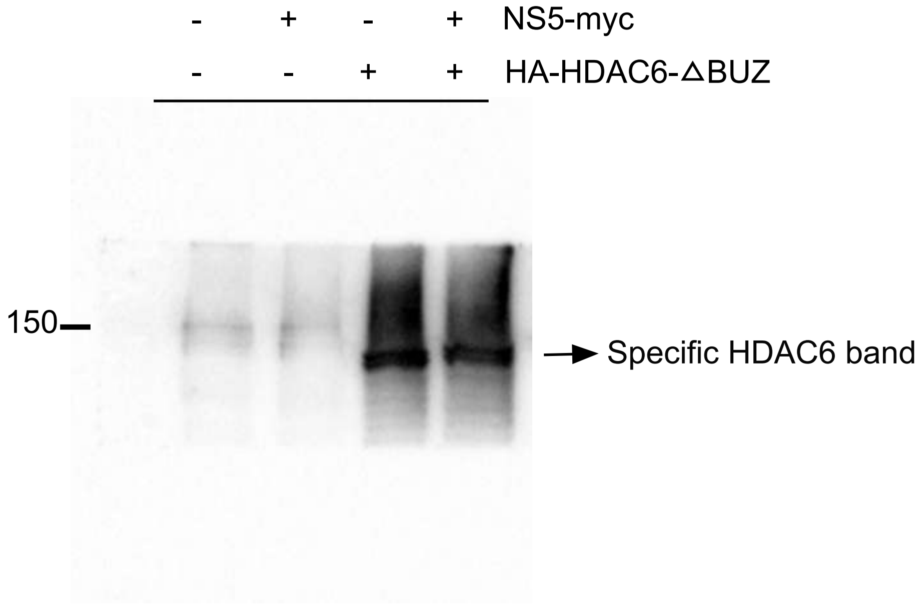

Figure S4A. *Replicate 1 myc complete gel*

Western-blot associated with Figure 4A
Pérez-Yanes, S., et al.

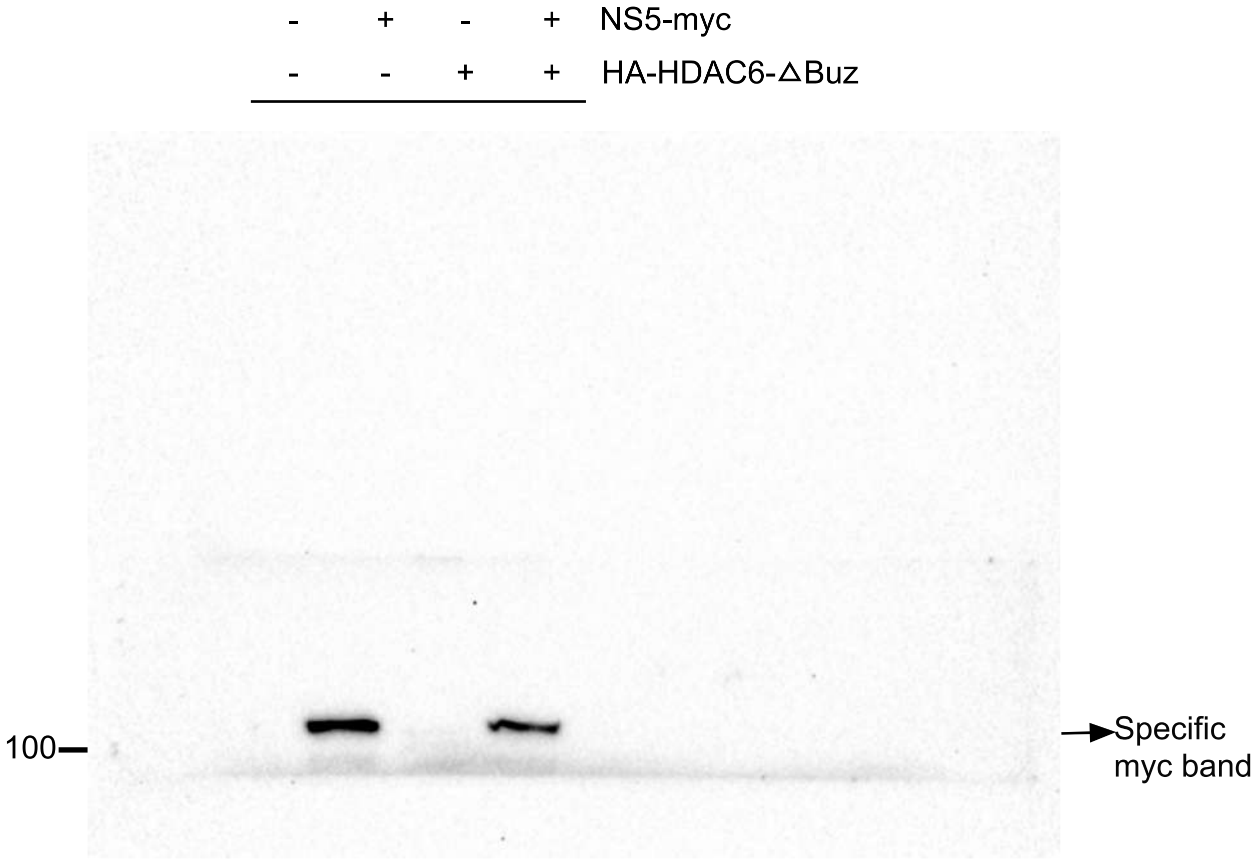

Figure S4A. *Replicate 1 p62 complete gel*

Western-blot associated with Figure 4A
Pérez-Yanes, S., et al.

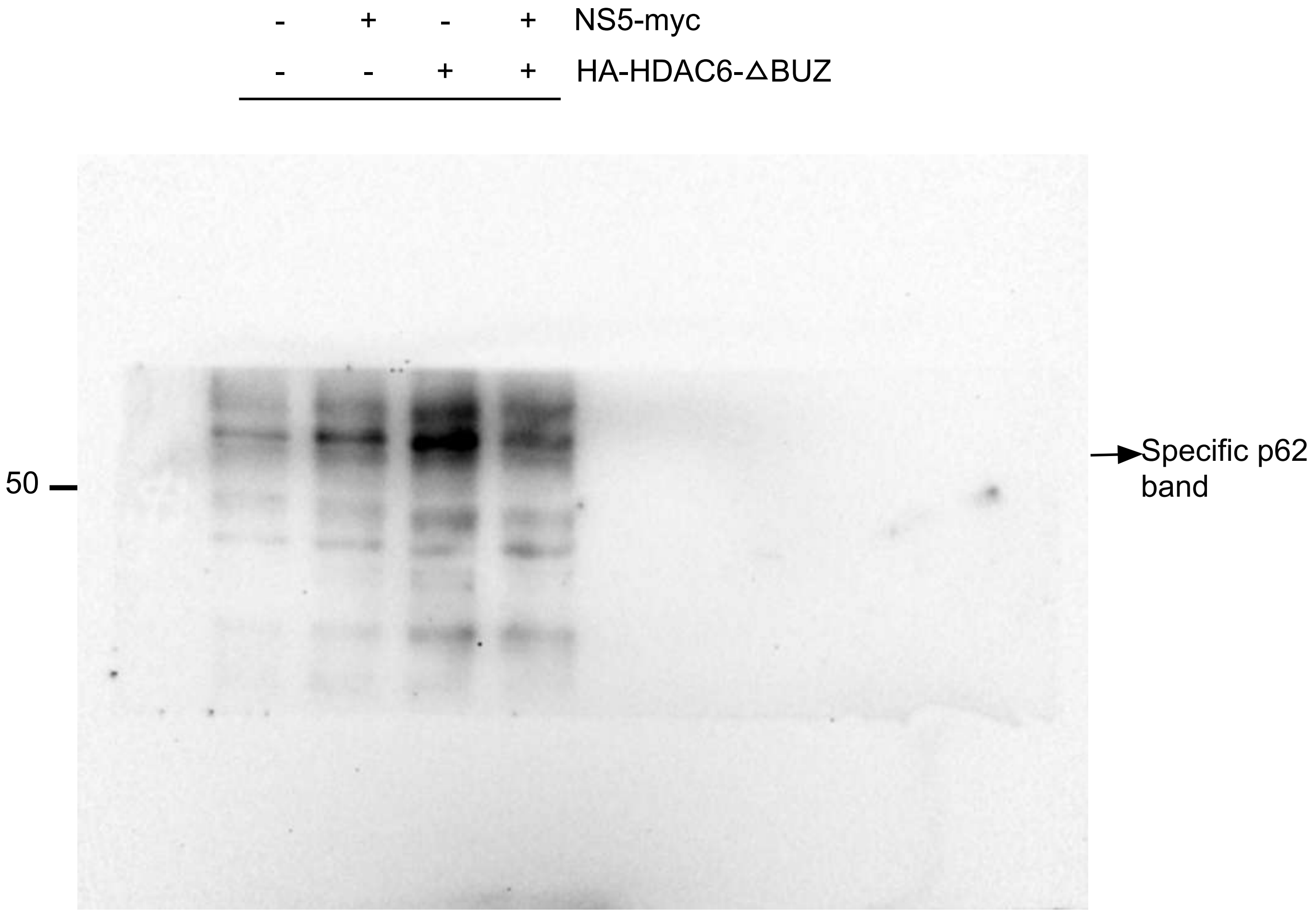

Figure S4A. *Replicate 1 Total α-Tubulin complete gel*

Western-blot associated with Figure 4A
Pérez-Yanes, S., et al.

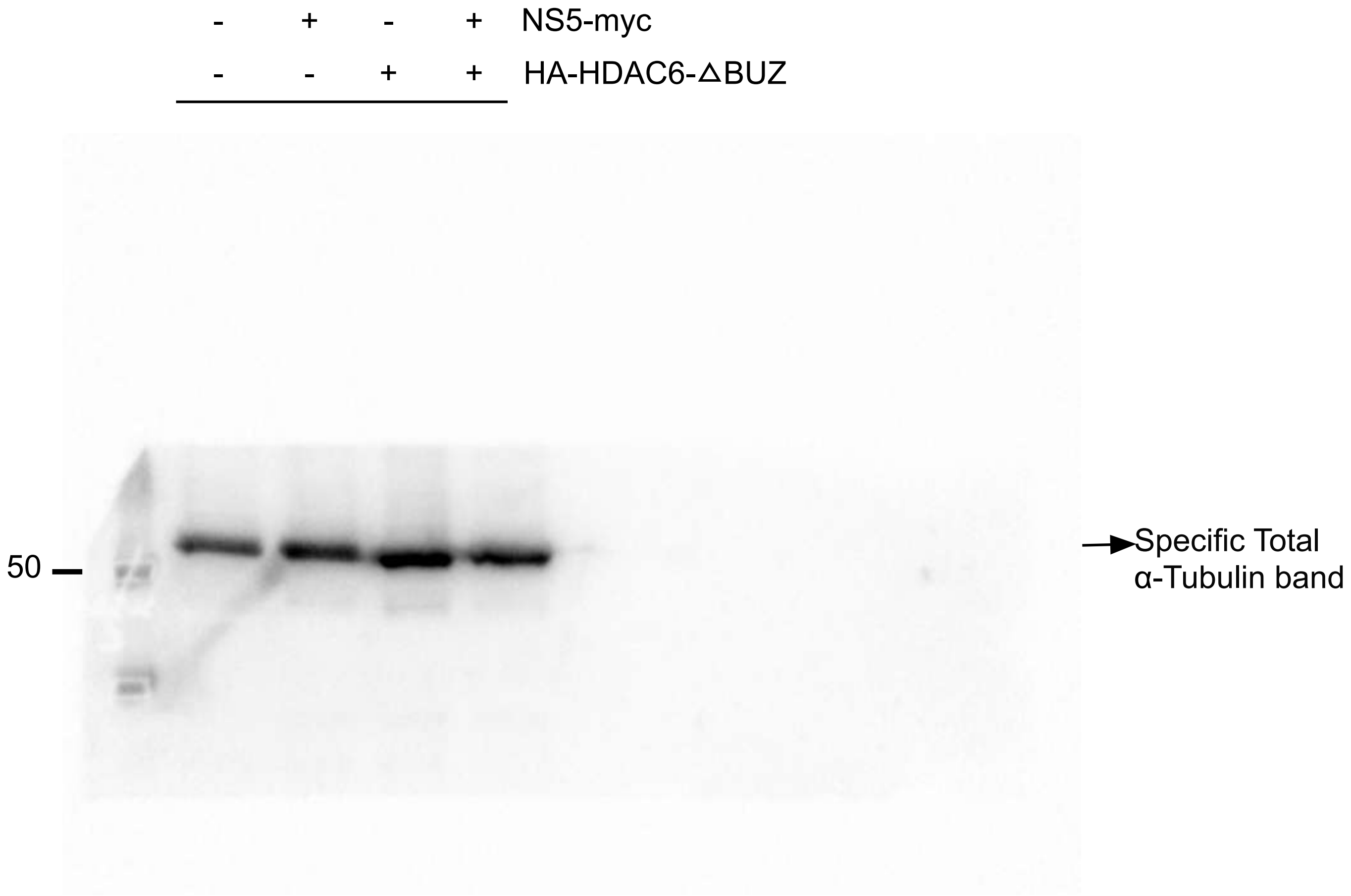

Figure S4A. *Replicate 1 Acetylated α-Tubulin complete gel*

Western-blot associated with Figure 4A
Pérez-Yanes, S., et al.

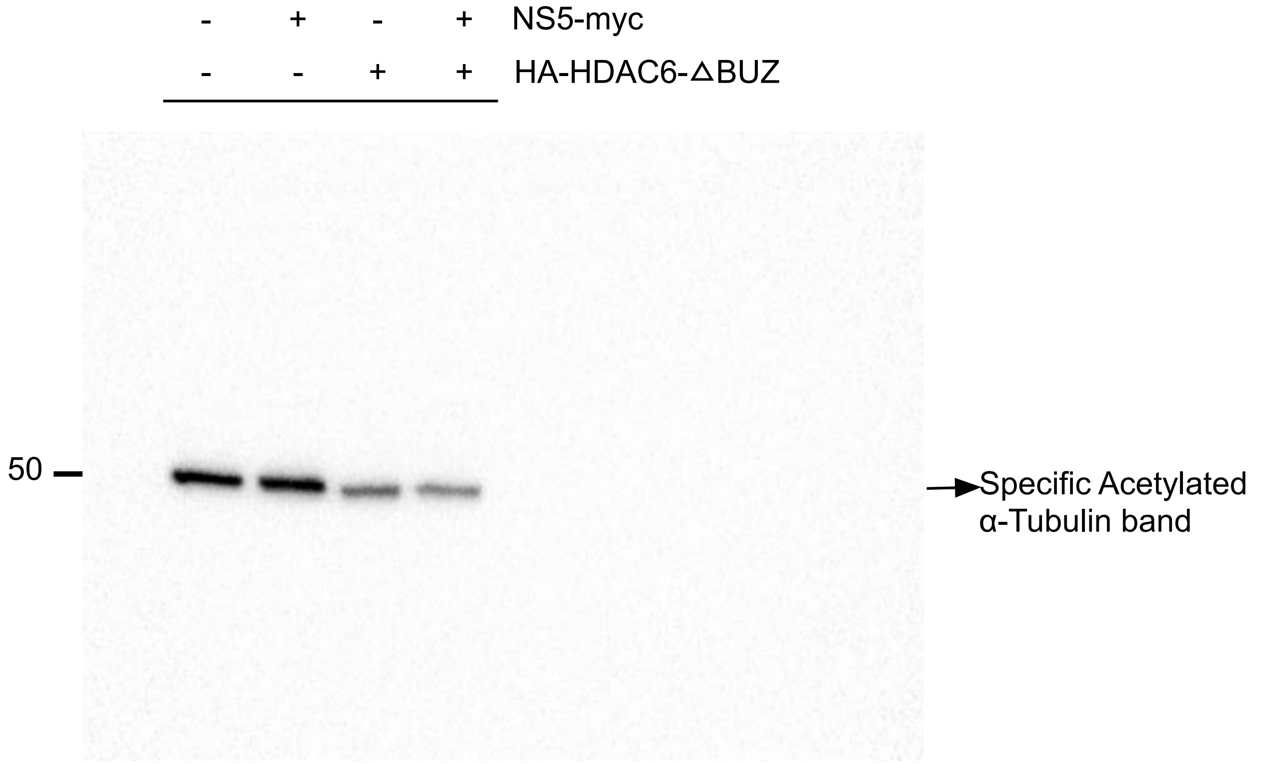

Figure S4A. *Replicate 2 HDAC6 complete gel*

Western-blot associated with Figure 4A
Pérez-Yanes, S., et al.

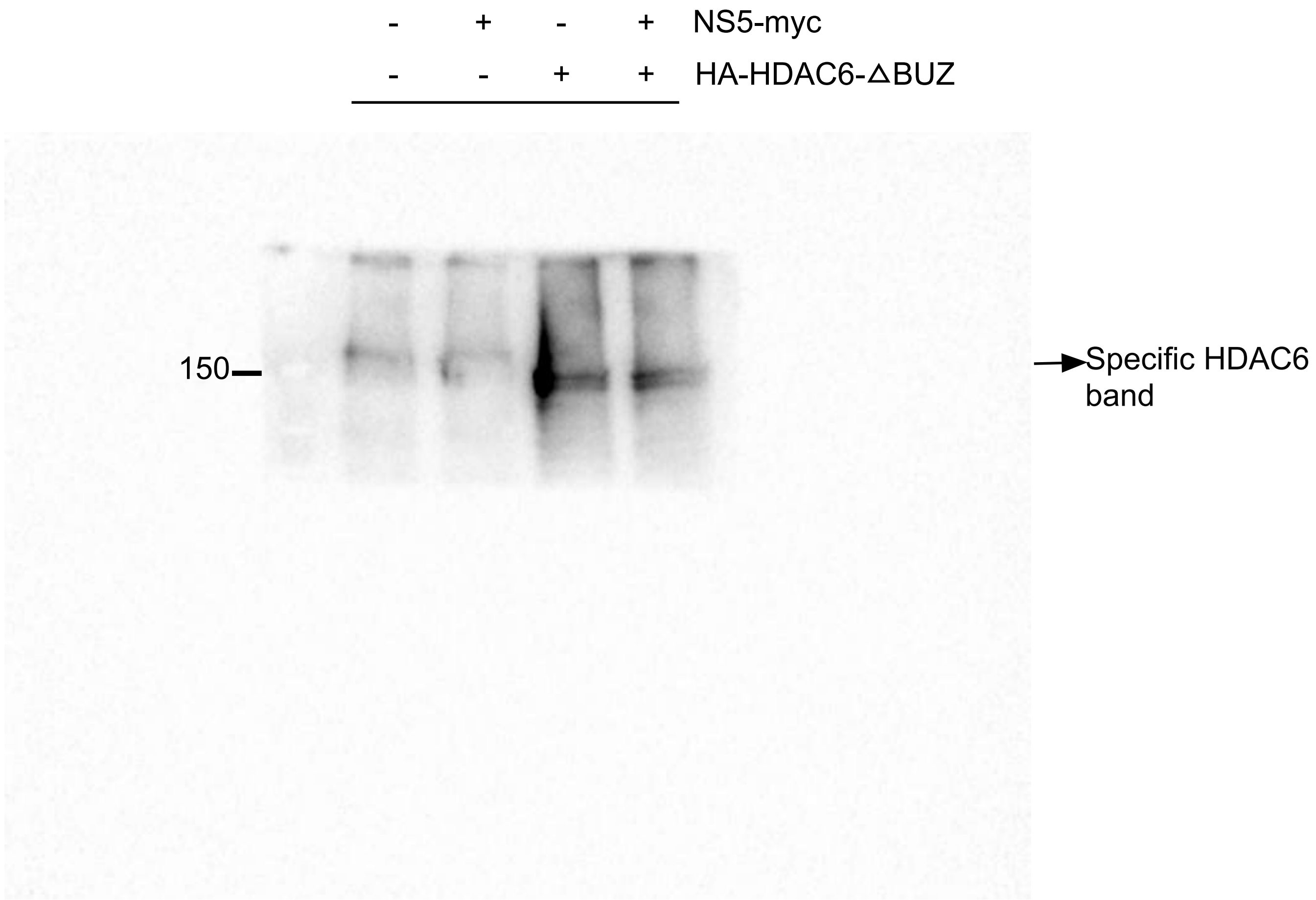

Figure S4A. *Replicate 2 myc complete gel*

Western-blot associated with Figure 4A
Pérez-Yanes, S., et al.

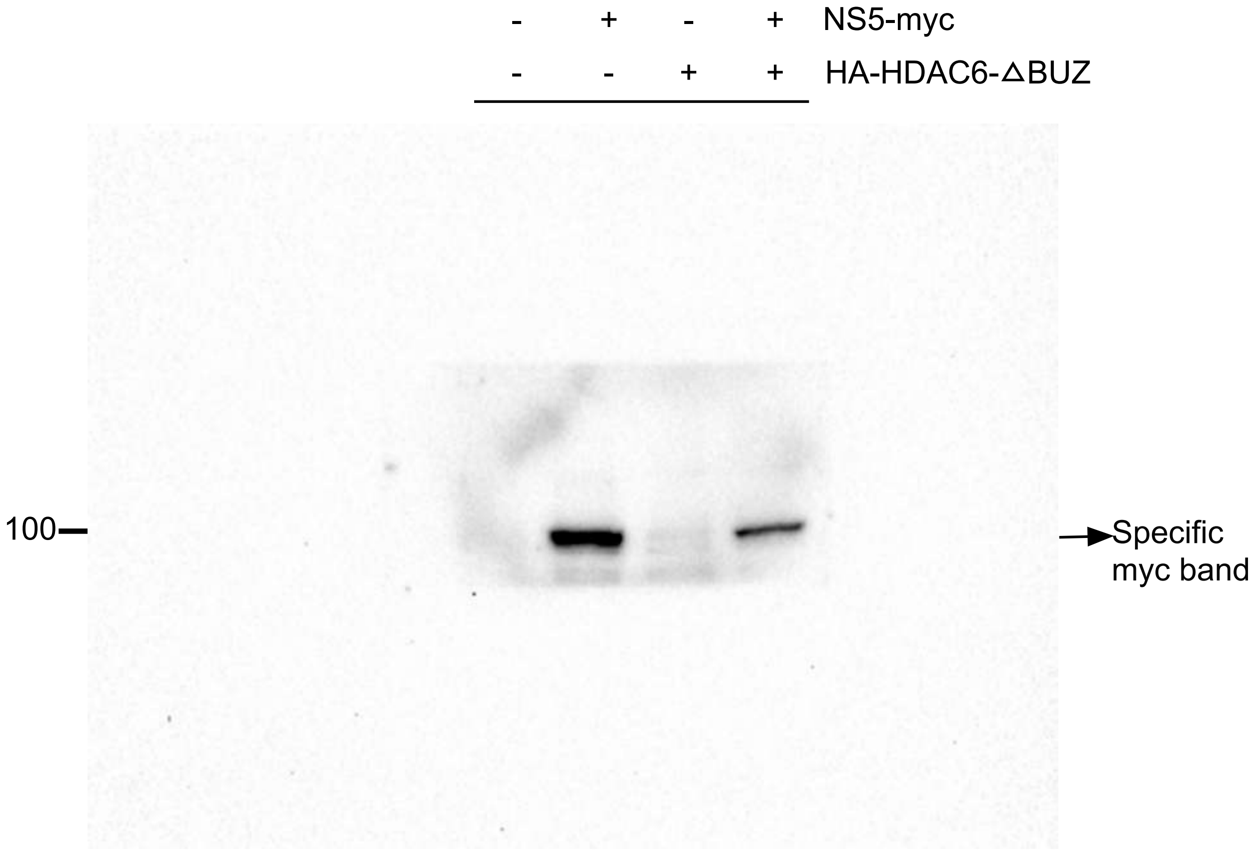

Figure S4A. *Replicate 2 p62 complete gel*

Western-blot associated with Figure 4A
Pérez-Yanes, S., et al.

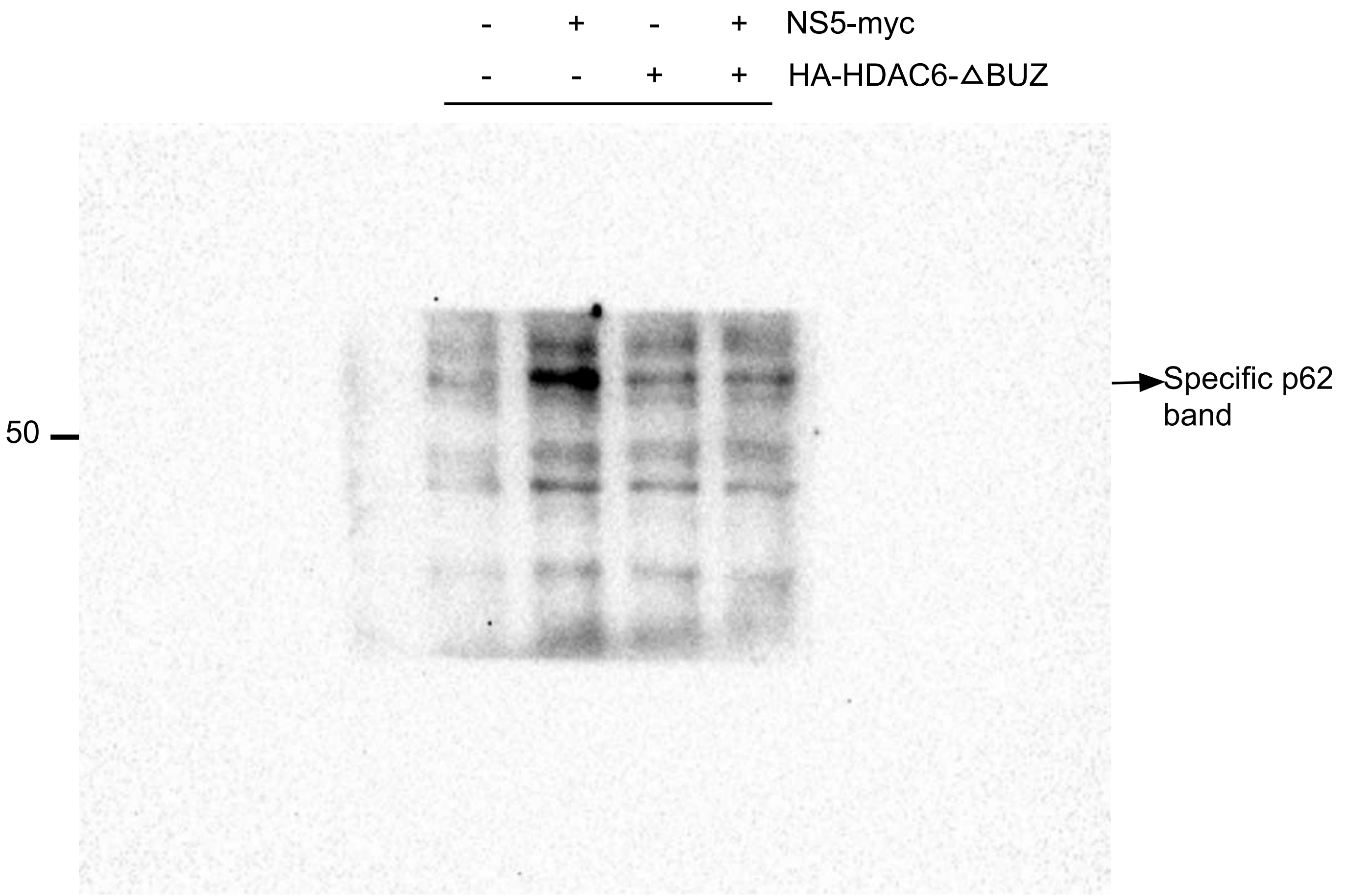

Figure S4A. *Replicate 2 Total α-Tubulin complete gel*

Western-blot associated with Figure 4A
Pérez-Yanes, S., et al.

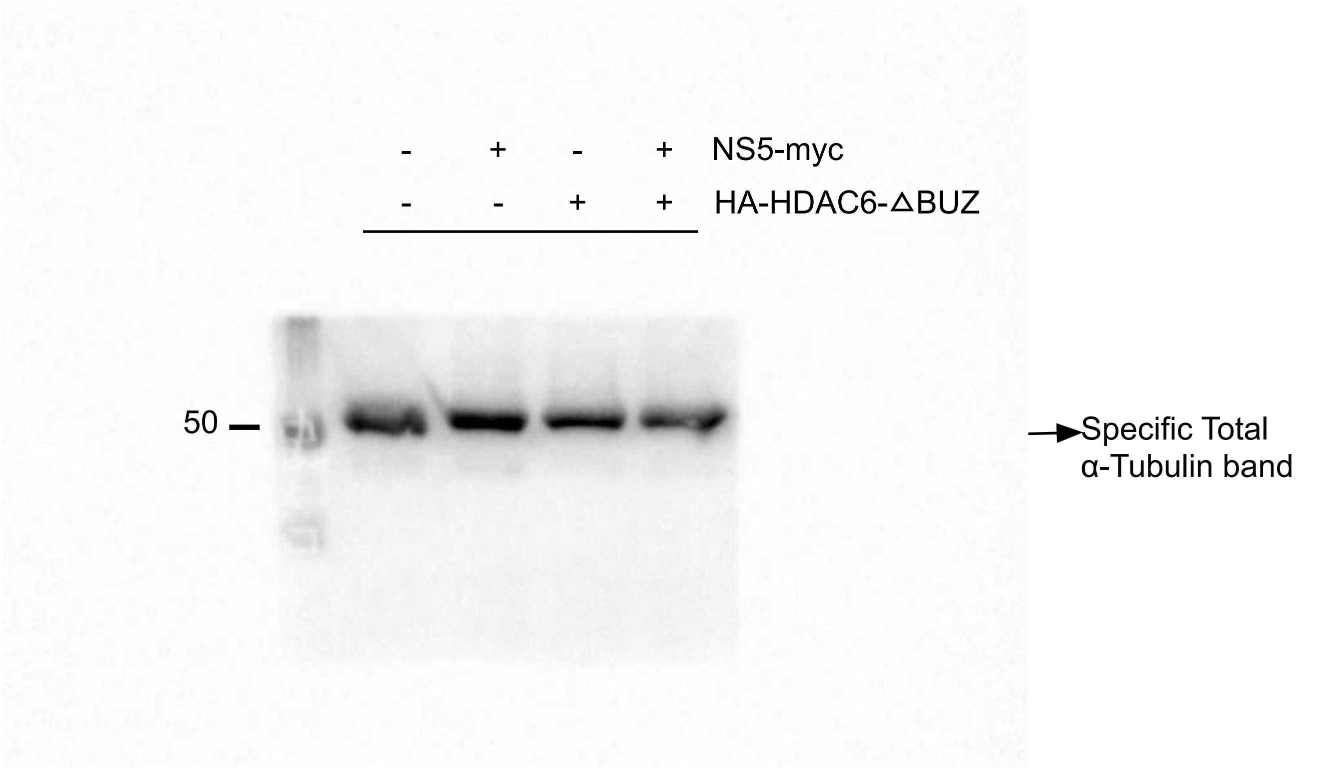

Figure S4A. *Replicate 2 Acetylated α-Tubulin complete gel*

Western-blot associated with Figure 4A
Pérez-Yanes, S., et al.

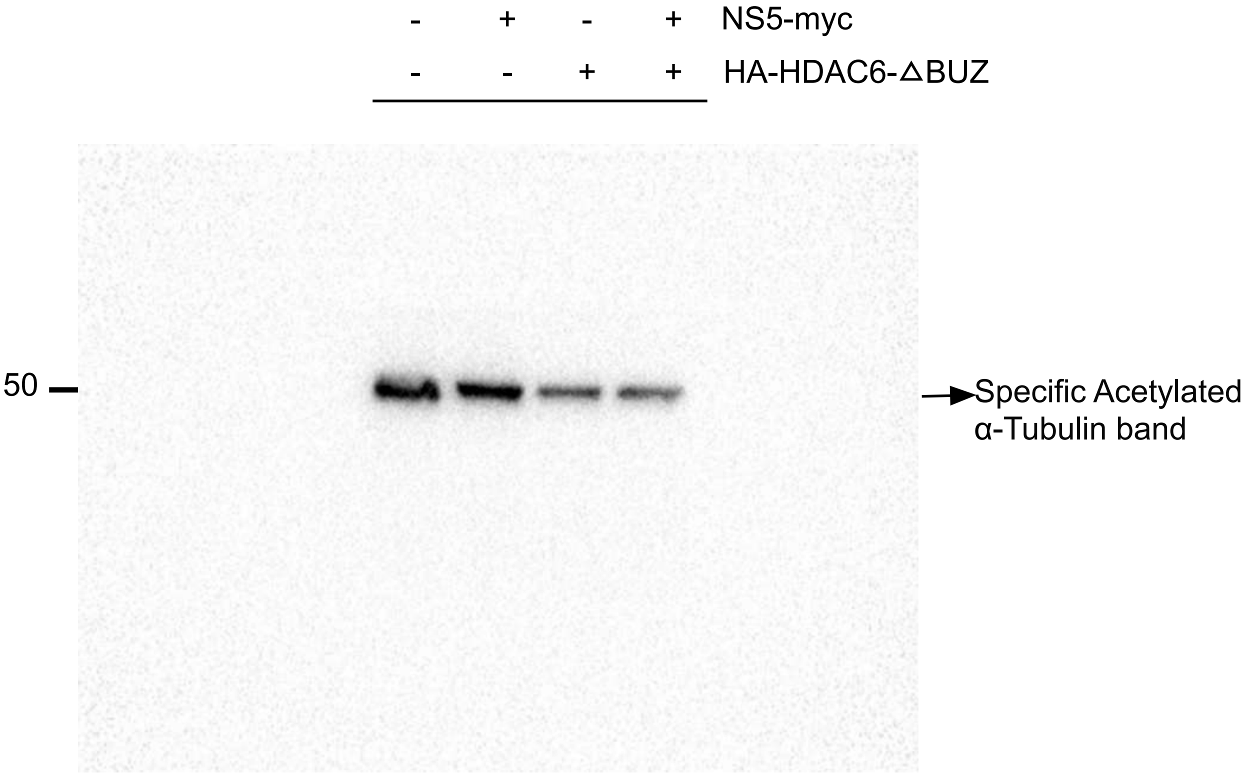

Figure S4A. *Replicate 3 HDAC6 complete gel*

Western-blot associated with Figure 4A
Pérez-Yanes, S., et al.

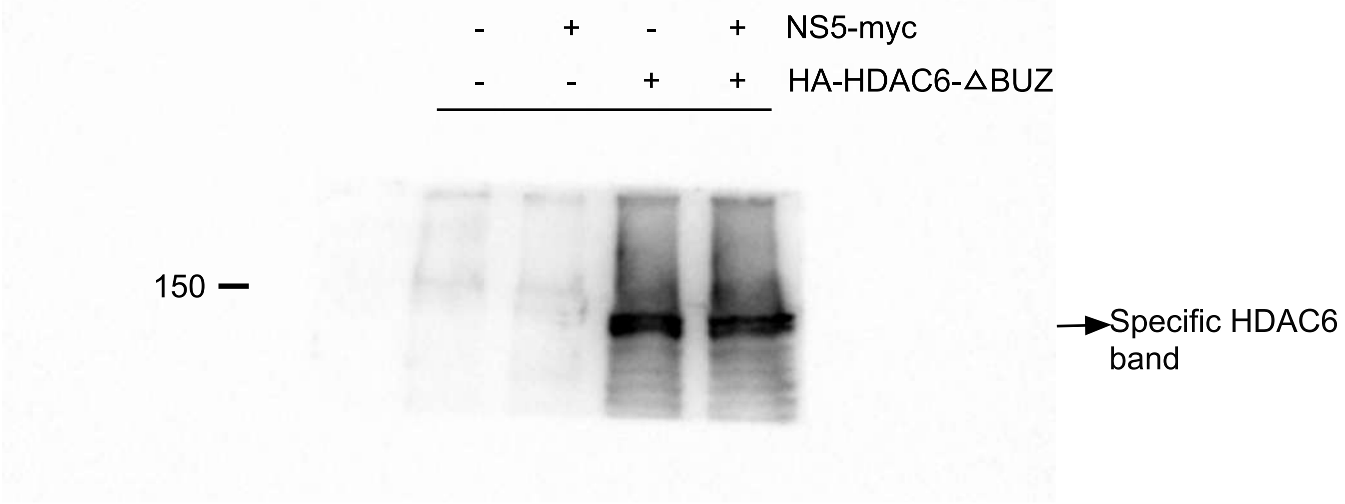

Figure S4A. *Replicate 3 myc complete gel*

Western-blot associated with Figure 4A
Pérez-Yanes, S., et al.

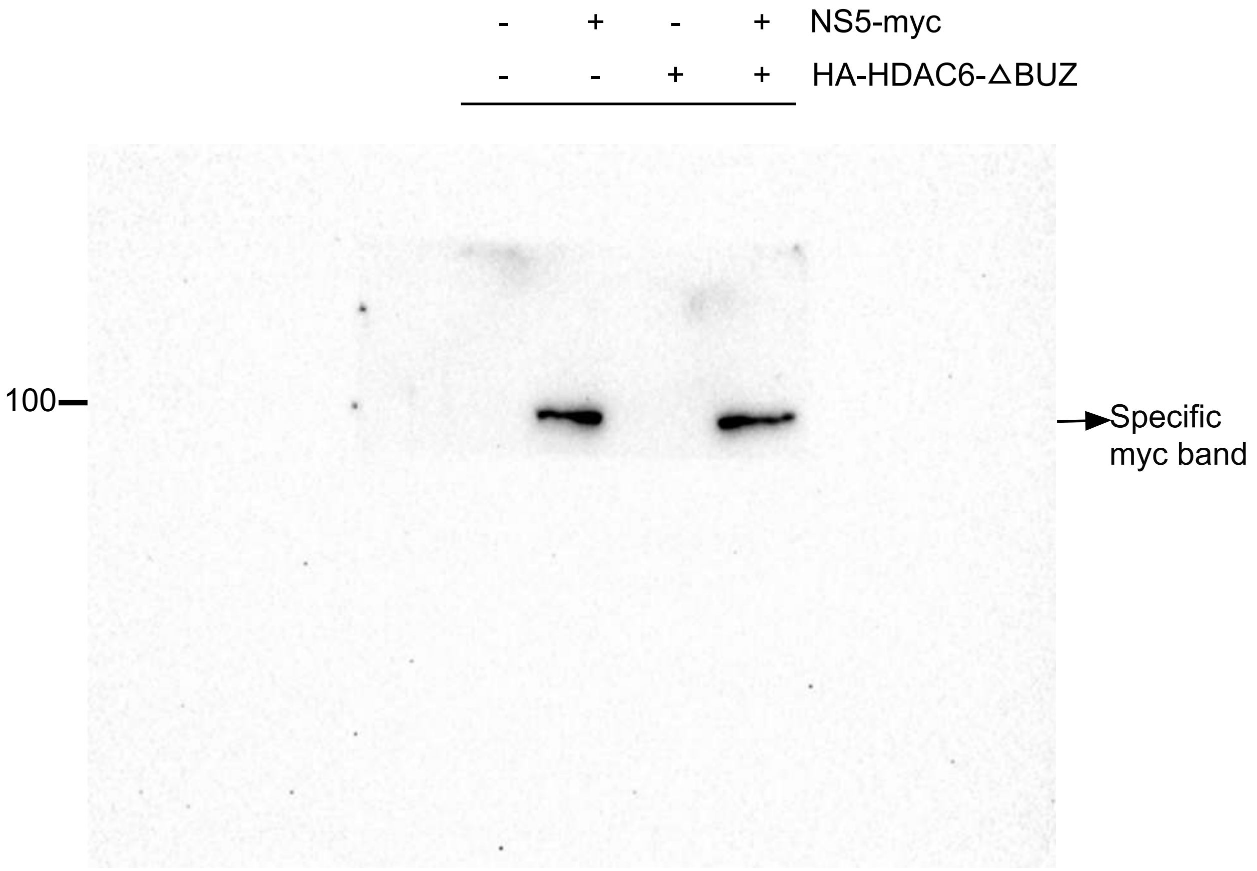

Figure S4A. *Replicate 3 p62 complete gel*

Western-blot associated with Figure 4A
Pérez-Yanes, S., et al.

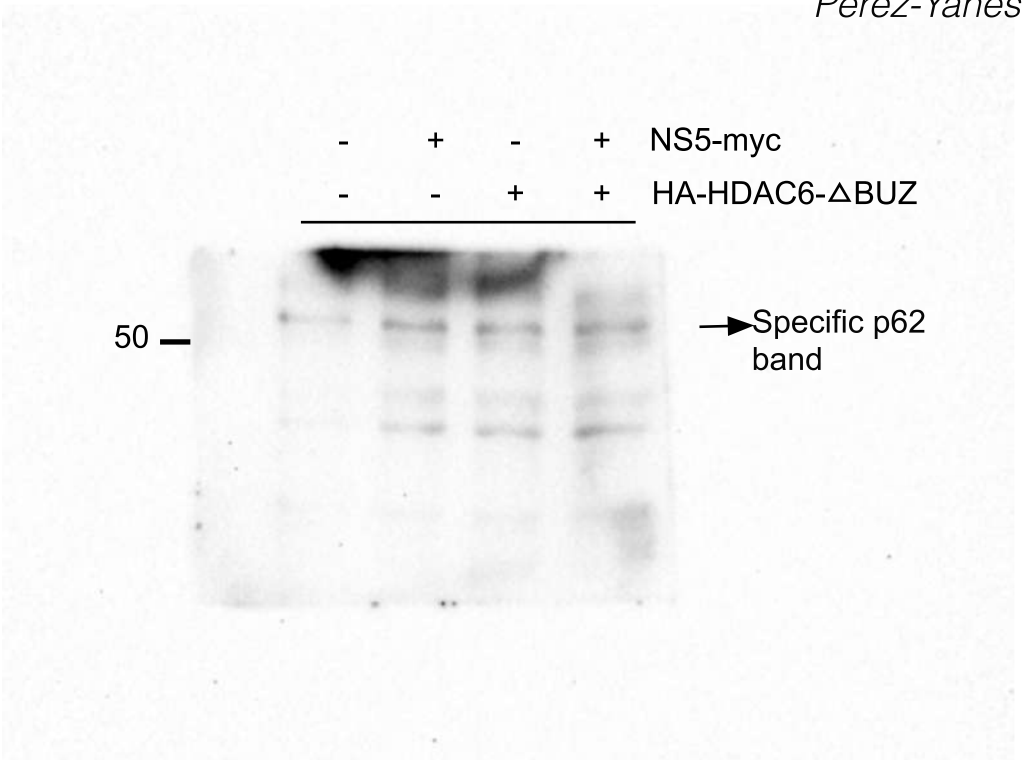

Figure S4A. *Replicate 3 Acetylated α-Tubulin complete gel*

Western-blot associated with Figure 4A
Pérez-Yanes, S., et al.

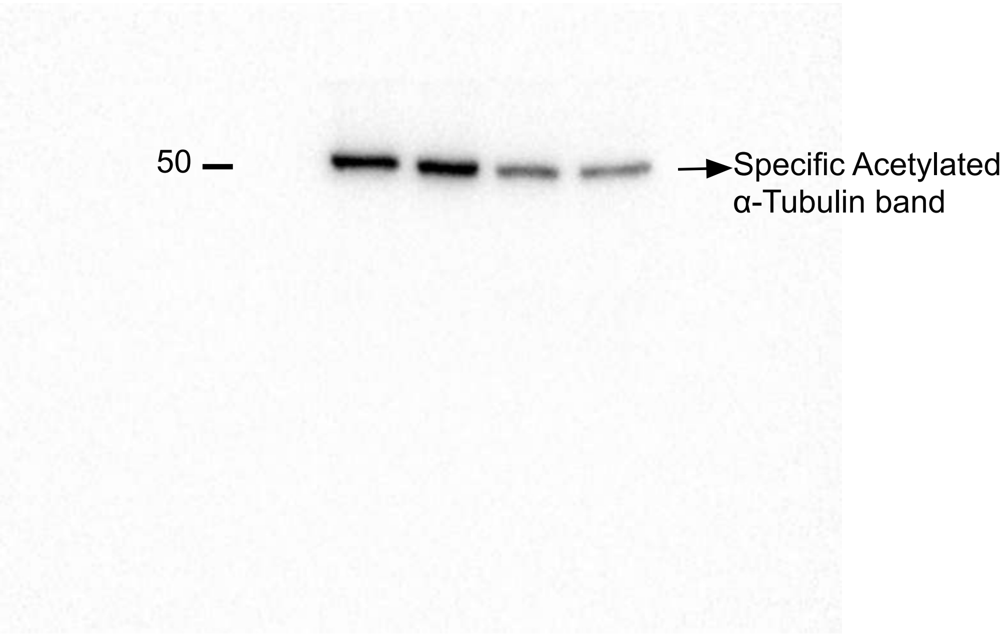

Figure S4A. *Replicate 3 Total α-Tubulin complete gel*

Western-blot associated with Figure 4A
Pérez-Yanes, S., et al.

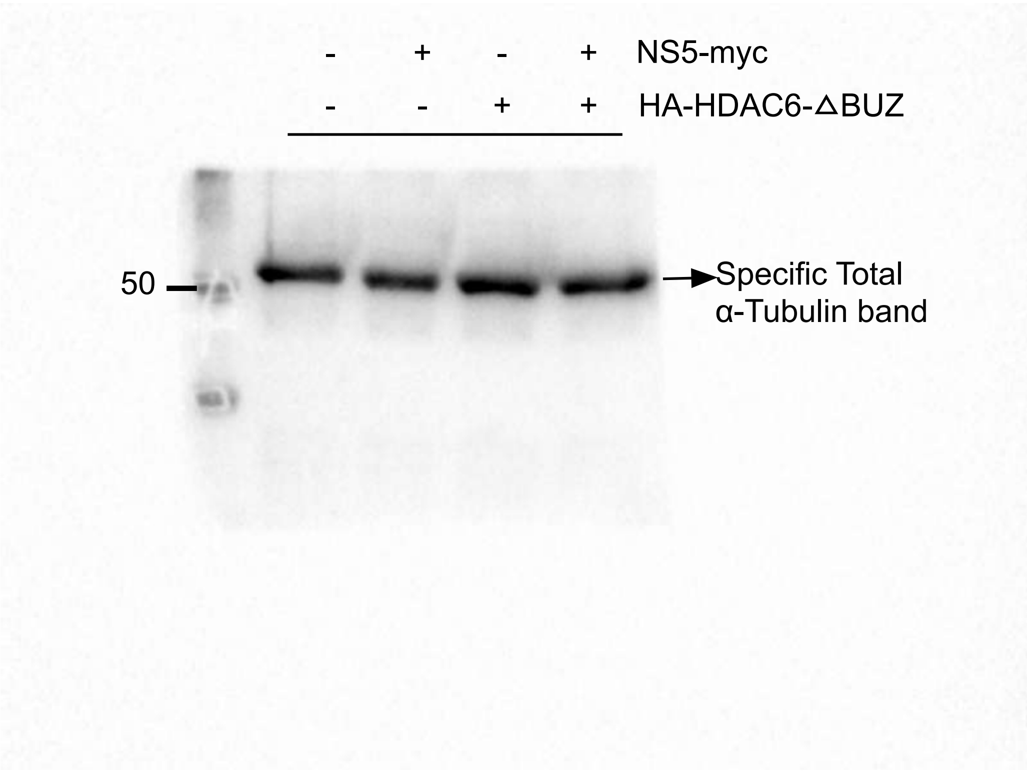

Figure S4C. Western-blot replicates associated with Figure 4C.

Replicate 1 as figure format

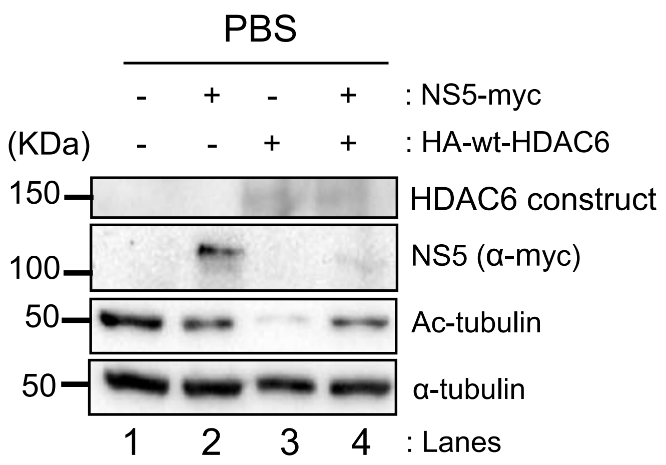

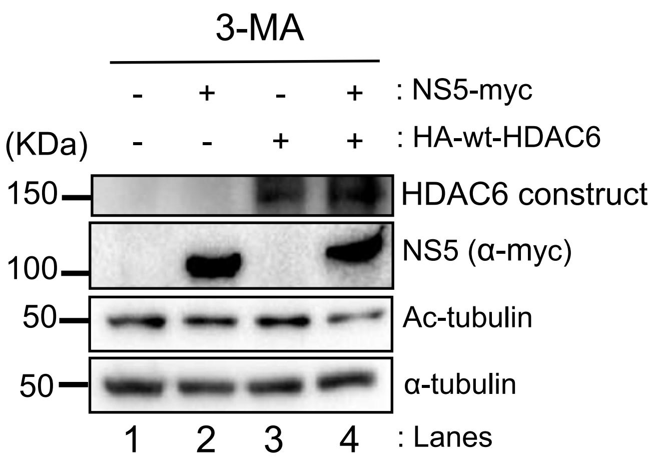

Replicate 2 as figure format

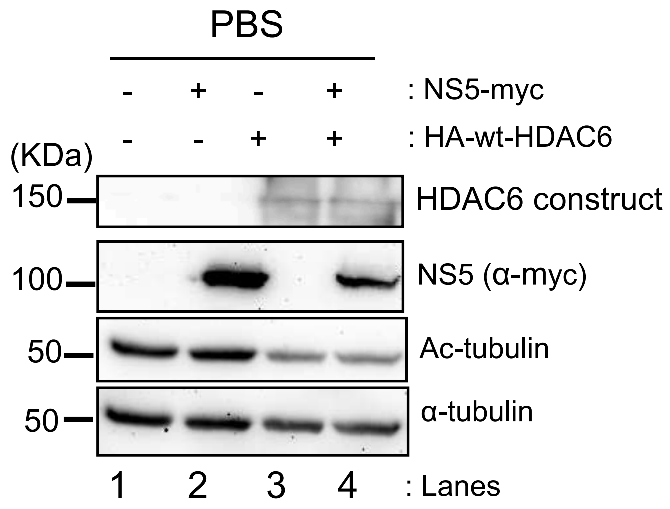

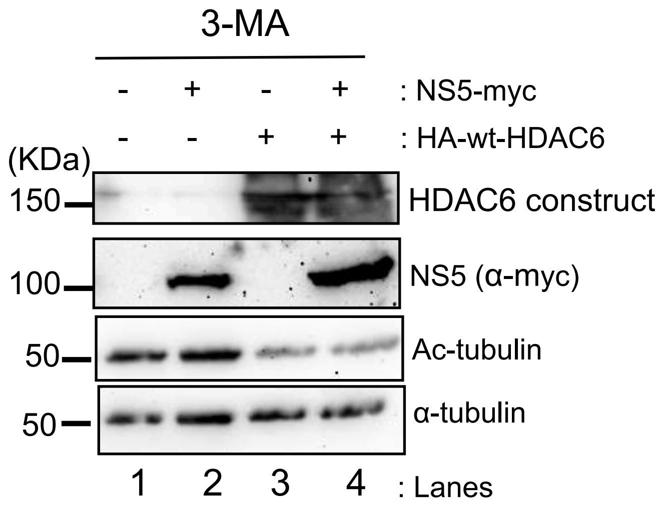

Figure S4C. *Replicate 1 HDAC6 complete gel*

Western-blot associated with Figure 4C
Pérez-Yanes, S., et al.

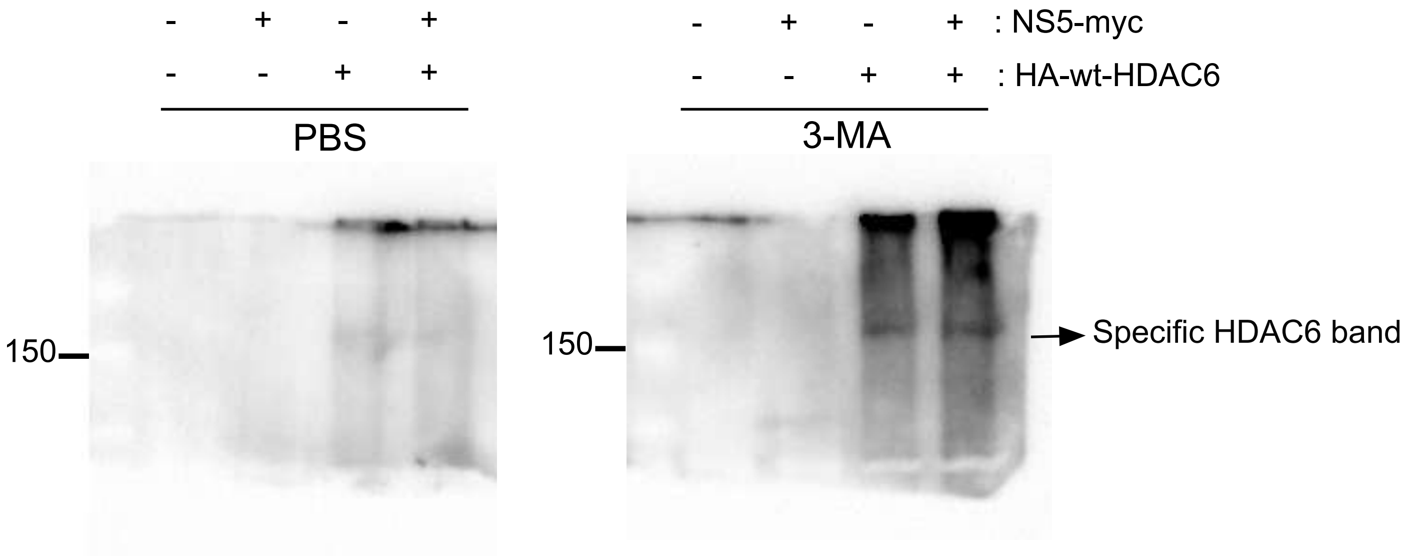

Figure S4C. *Replicate 1 myc complete gel*

Western-blot associated with Figure 4C
Pérez-Yanes, S., et al.

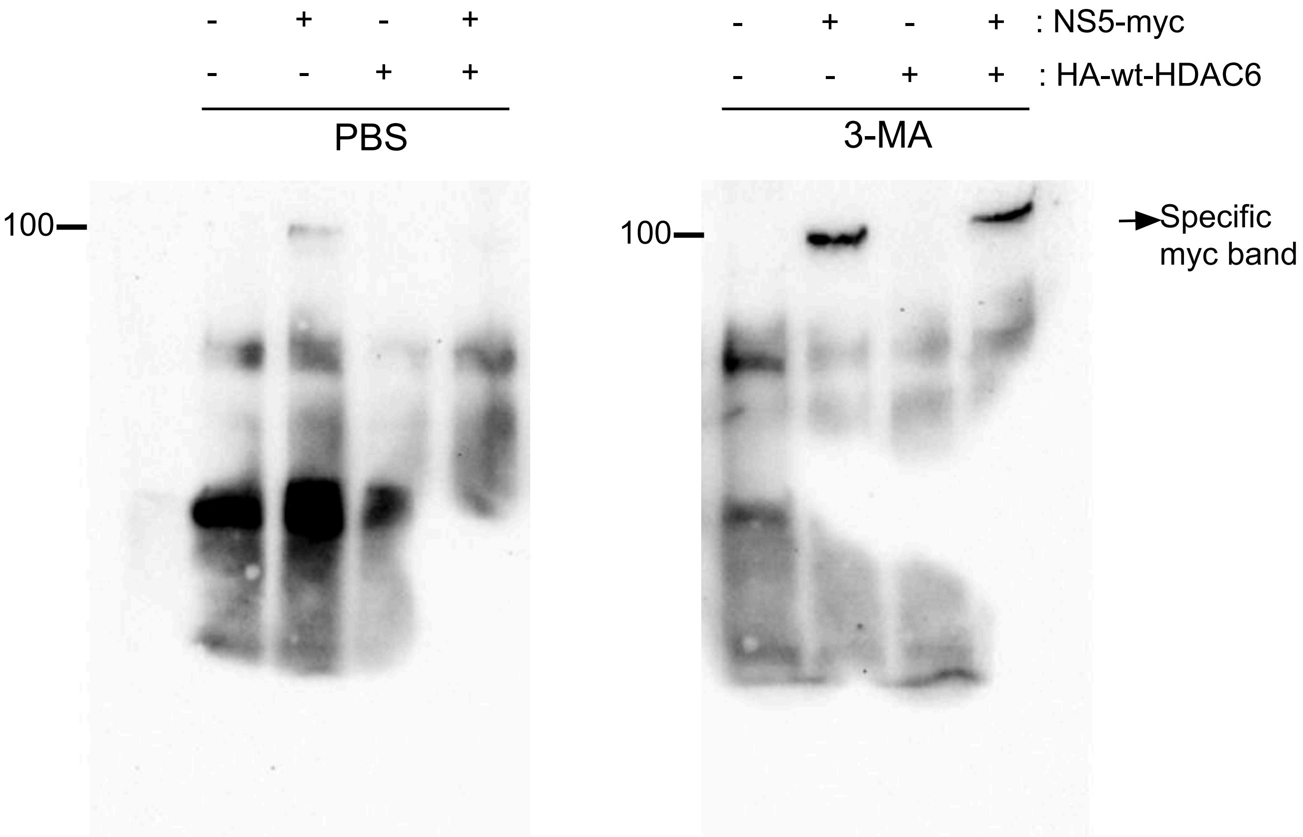

Figure S4C. *Replicate 1 Acetylated α-Tubulin complete gel*

Western-blot associated with Figure 4C
Pérez-Yanes, S., et al.

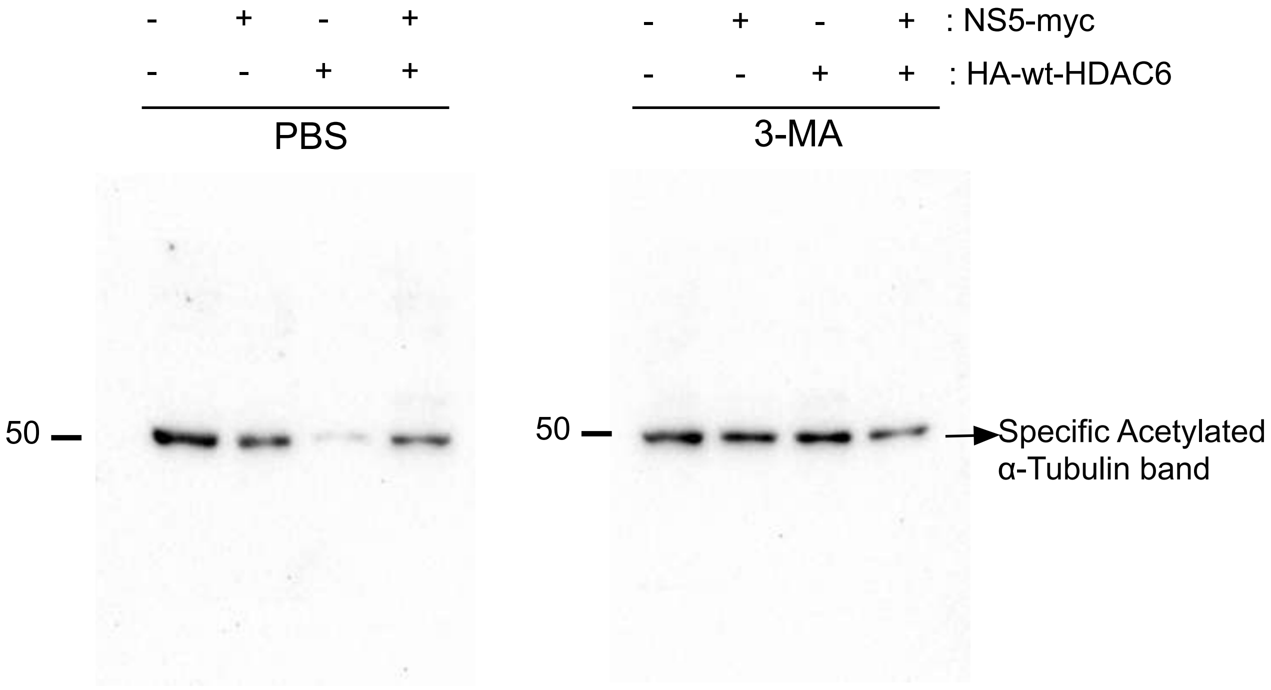

Figure S4C. *Replicate 2 HDAC6 complete gel*

Western-blot associated with Figure 4C
Pérez-Yanes, S., et al.

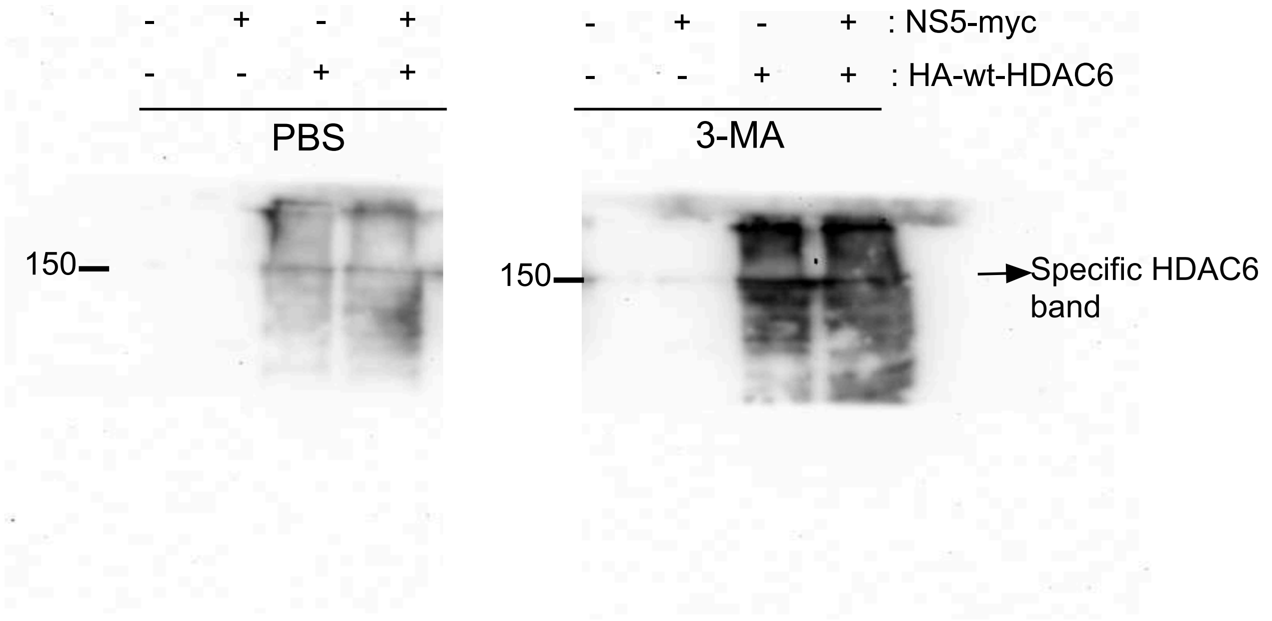

Figure S4C. *Replicate 2 myc complete gel*

Western-blot associated with Figure 4C
Pérez-Yanes, S., et al.

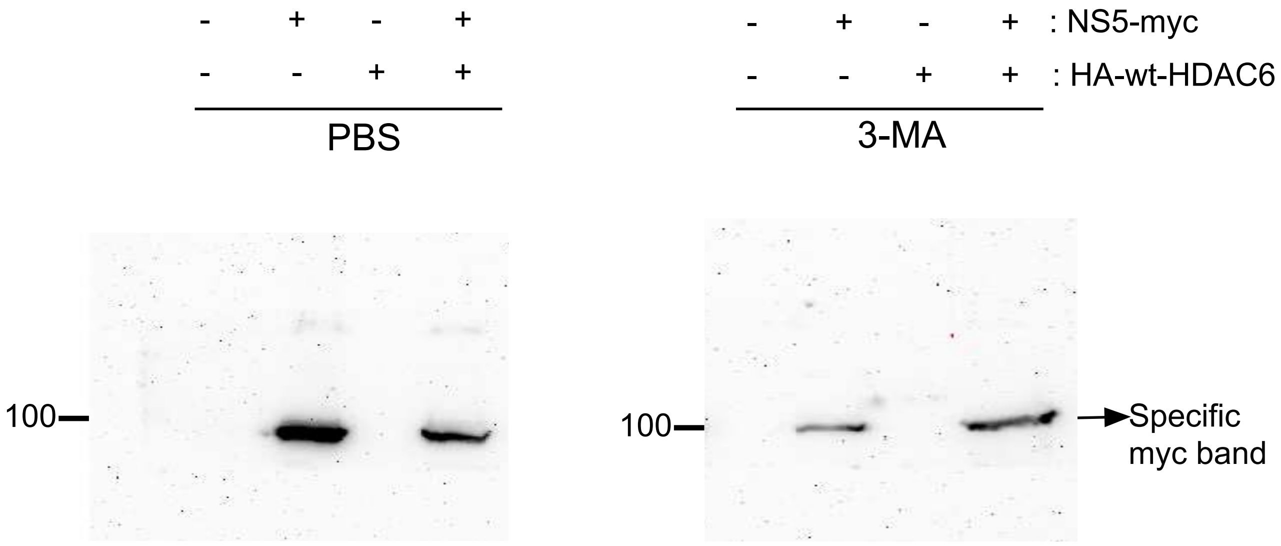

Figure S4C. *Replicate 2 Total α-Tubulin complete gel*

Western-blot associated with Figure 4C
Pérez-Yanes, S., et al.

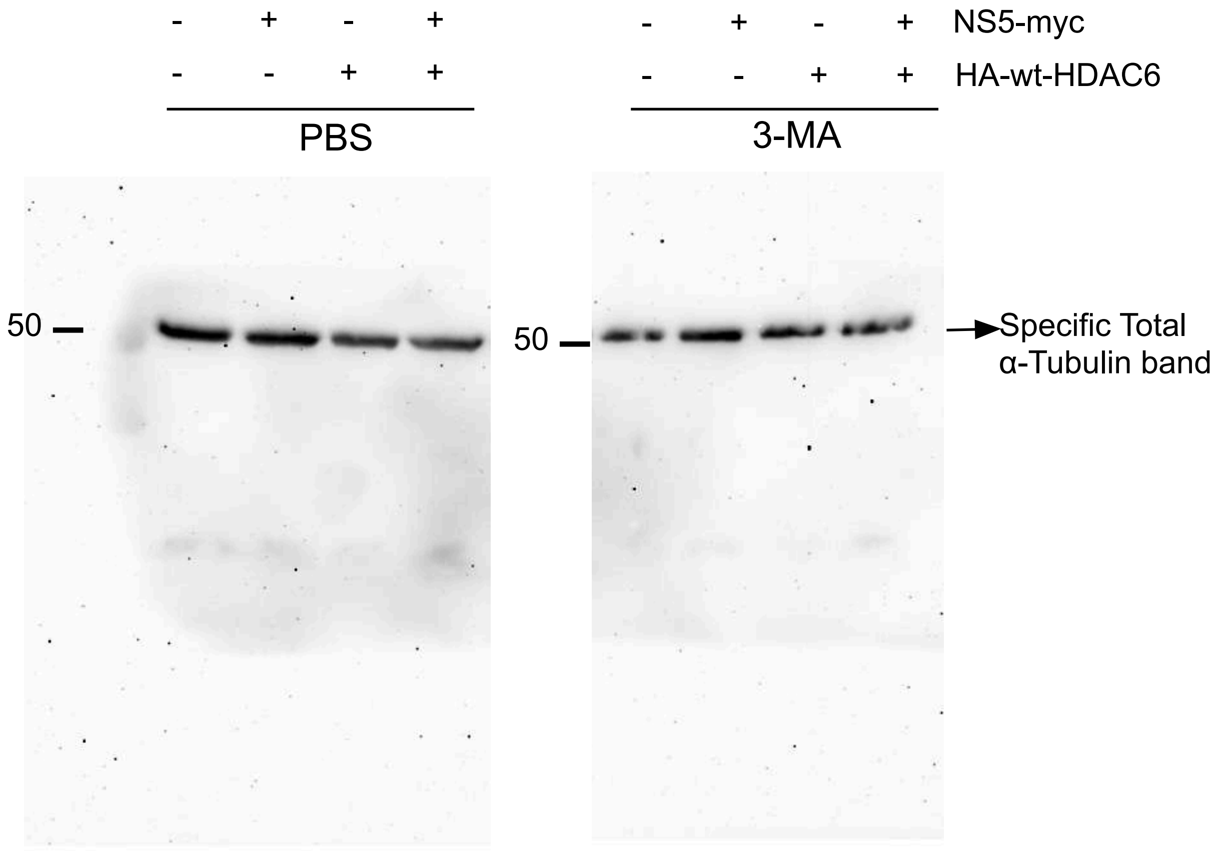

Figure S4C. *Replicate 2 Acetylated α-Tubulin complete gel*

Western-blot associated with Figure 4C
Pérez-Yanes, S., et al.

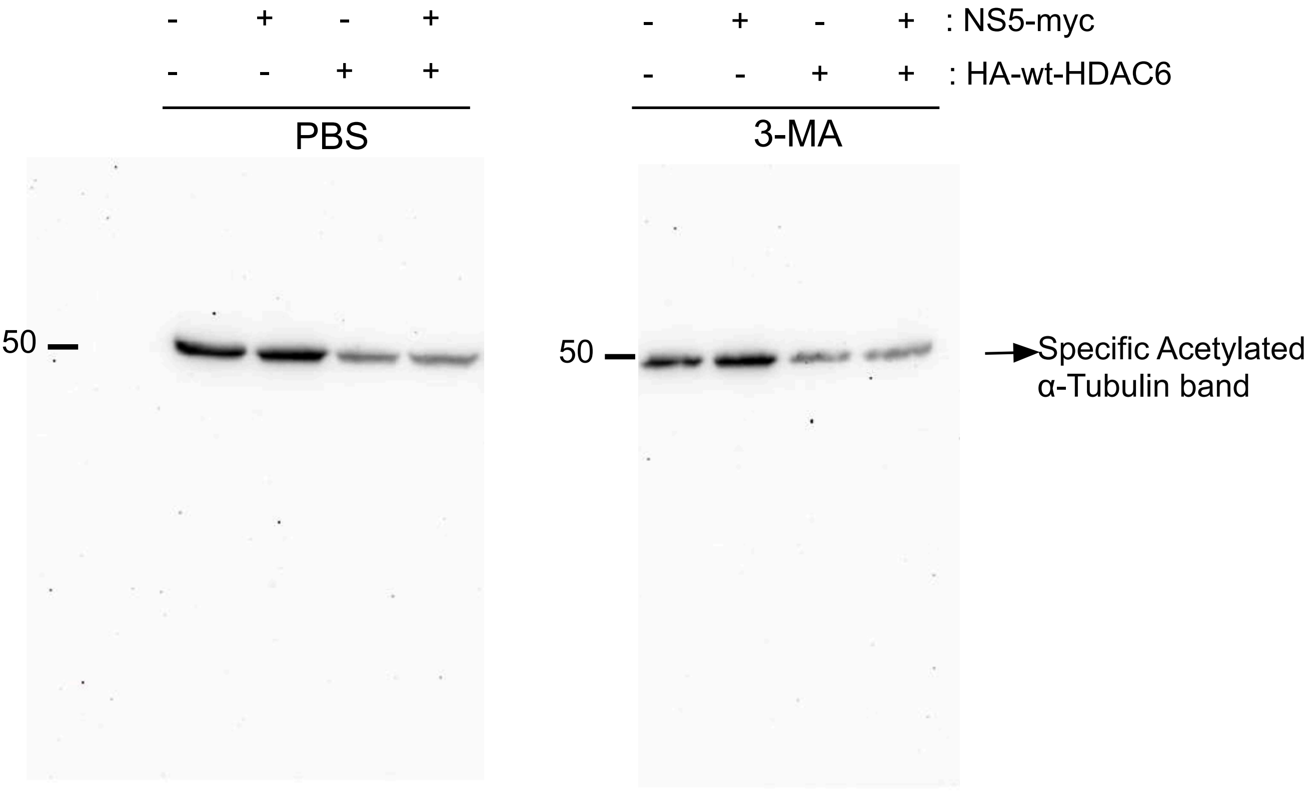

Figura S4D. Western-blot replicates associated with Figure 4D.

Replicate 1 as figure format

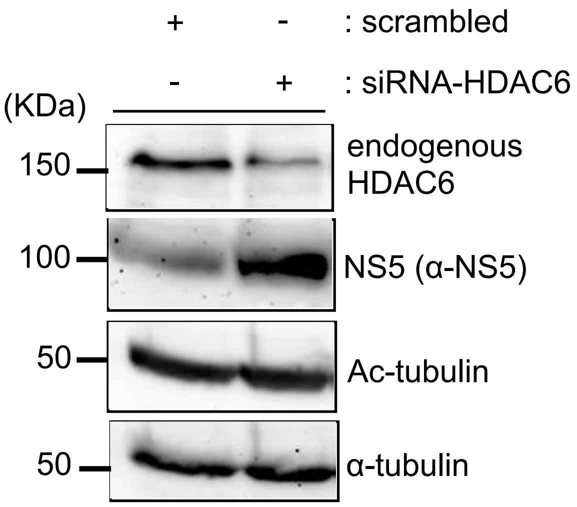

Replicate 2 as figure format

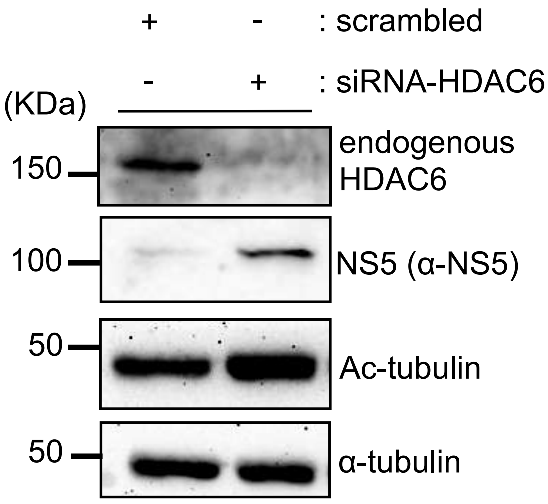

Replicate 3 as figure format

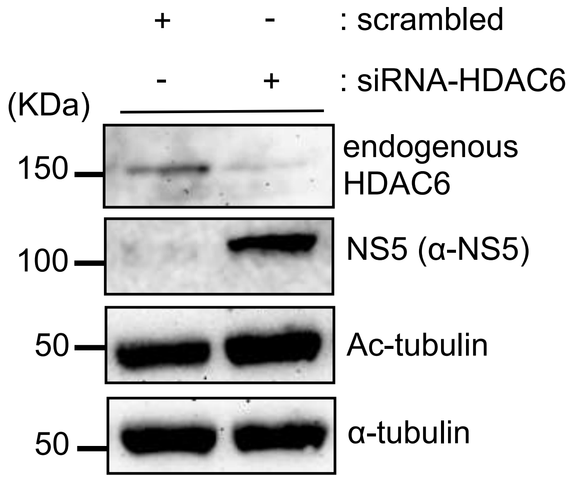

Replicate 4 as figure format

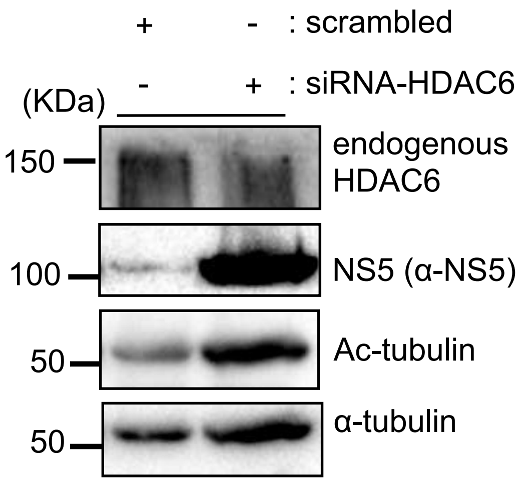

Figure S4D. *Replicate 1 HDAC6 complete gel*

Western-blot associated with Figure 4D
Pérez-Yanes, S., et al.

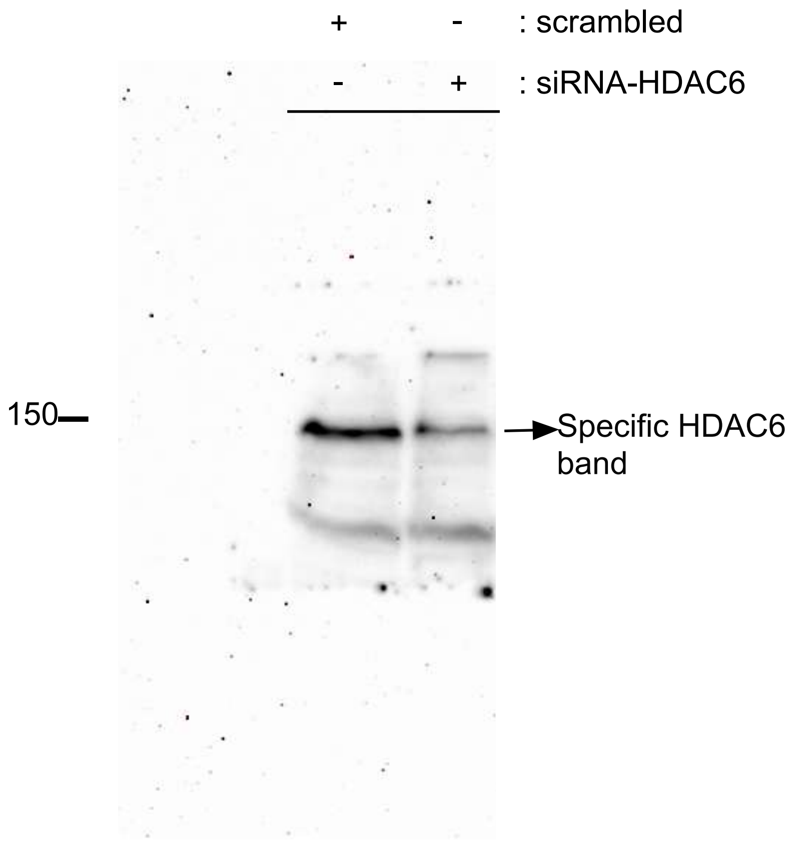

Figure S4D. *Replicate 1 myc complete gel*

Western-blot associated with Figure 4D
Pérez-Yanes, S., et al.

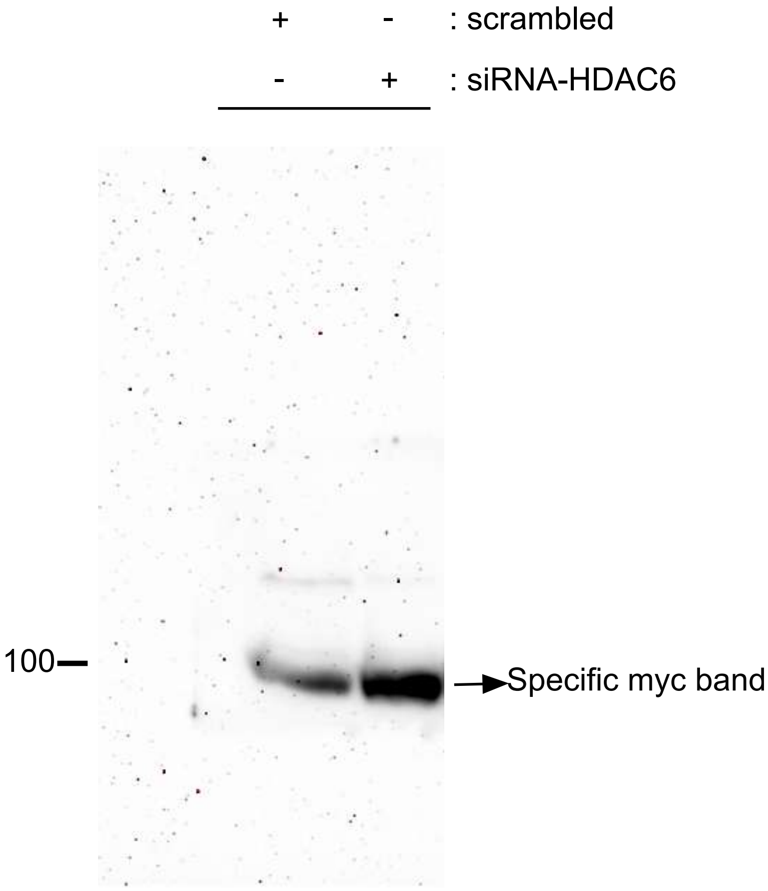

Figure S4D. *Replicate 1 Total α-Tubulin complete gel*

Western-blot associated with Figure 4D
Pérez-Yanes, S., et al.

+	-	: scrambled
-	+	: siRNA-HDAC6

50 —

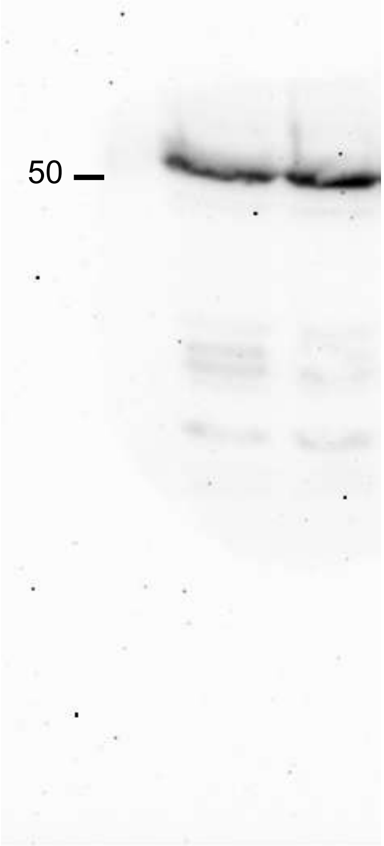

→ Specific Total α-Tubulin band

Figure S4D. *Replicate 1 Acetylated α-Tubulin complete gel*

Western-blot associated with Figure 4D
Pérez-Yanes, S., et al.

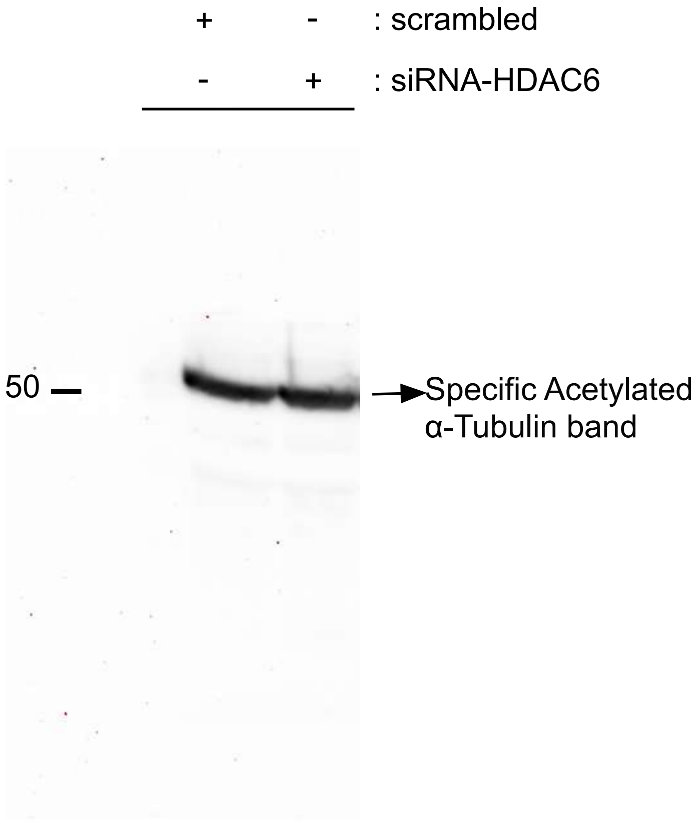

Figure S4D. *Replicate 2 HDAC6 complete gel*

Western-blot associated with Figure 4D
Pérez-Yanes, S., et al.

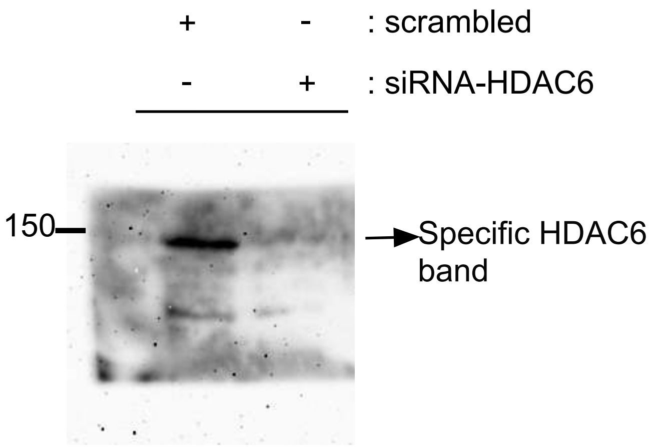

Figure S4D. *Replicate 2 myc complete gel*

Western-blot associated with Figure 4D
Pérez-Yanes, S., et al.

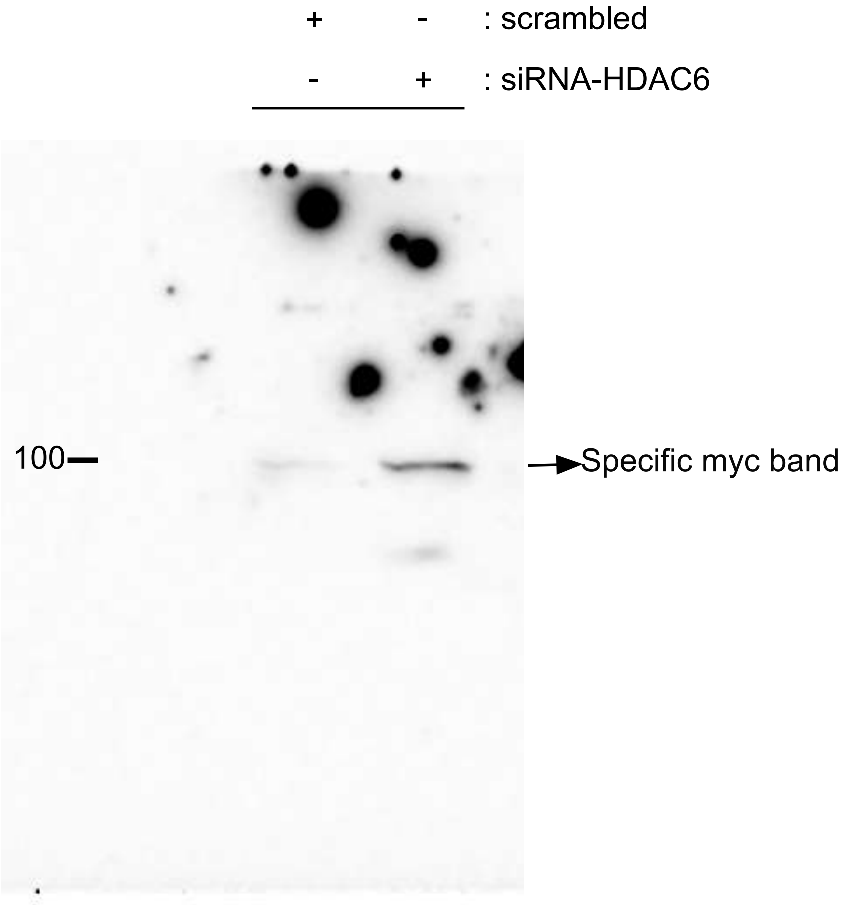

Figure S4D. *Replicate 2 Total α-Tubulin complete gel*

Western-blot associated with Figure 4D
Pérez-Yanes, S., et al.

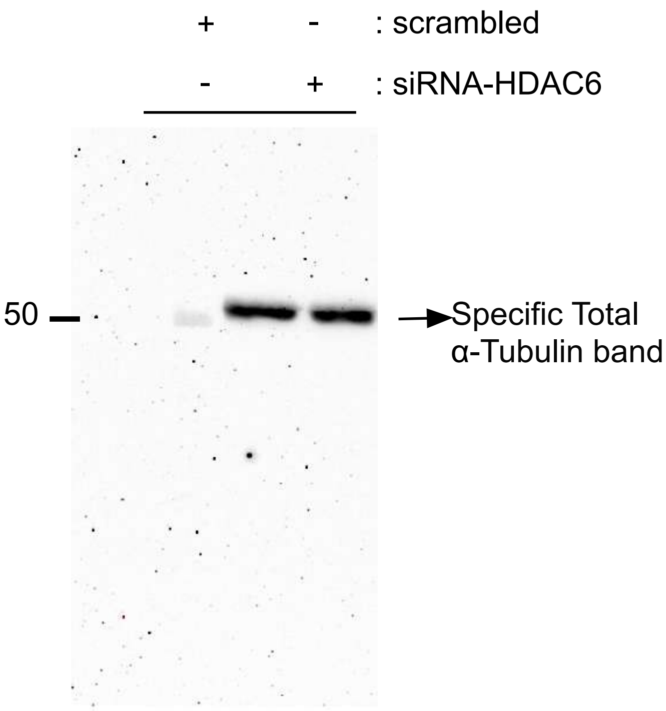

Figure S4D. *Replicate 2 Acetylated α-Tubulin complete gel*

Western-blot associated with Figure 4D
Pérez-Yanes, S., et al.

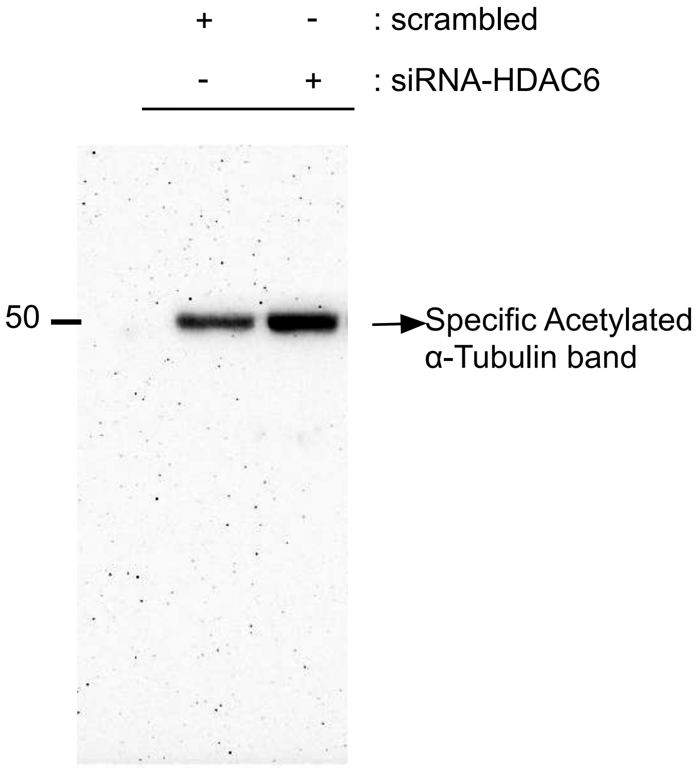

Figure S4D. *Replicate 3 HDAC6 complete gel*

Western-blot associated with Figure 4D
Pérez-Yanes, S., et al.

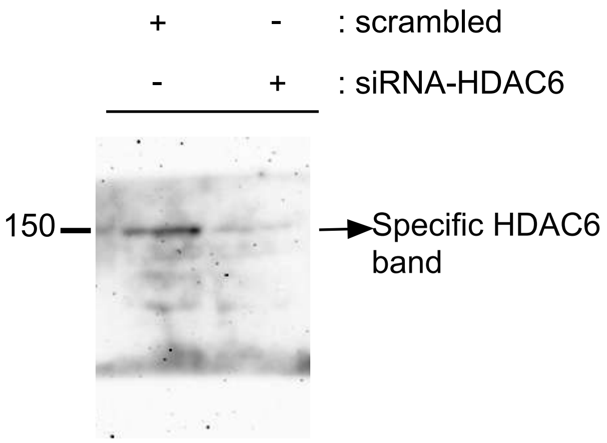

Figure S4D. *Replicate 3 myc complete gel*

Western-blot associated with Figure 4D
Pérez-Yanes, S., et al.

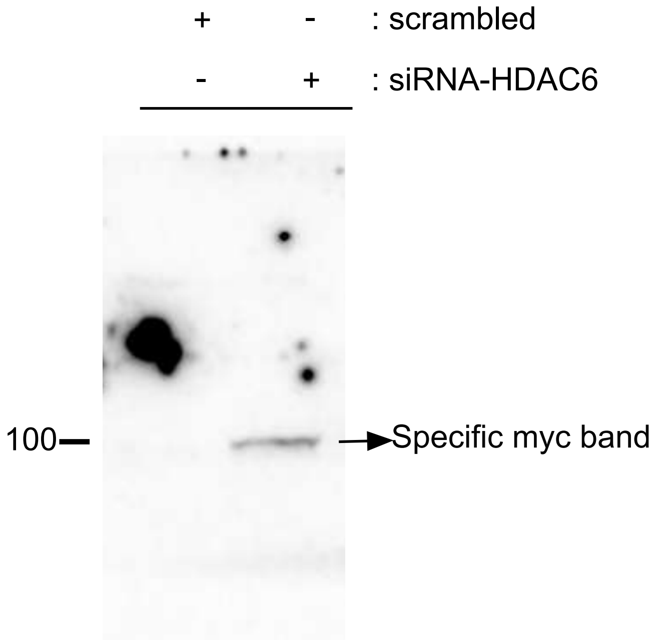

Figure S4D. *Replicate 3 Total α-Tubulin complete gel*

Western-blot associated with Figure 4D
Pérez-Yanes, S., et al.

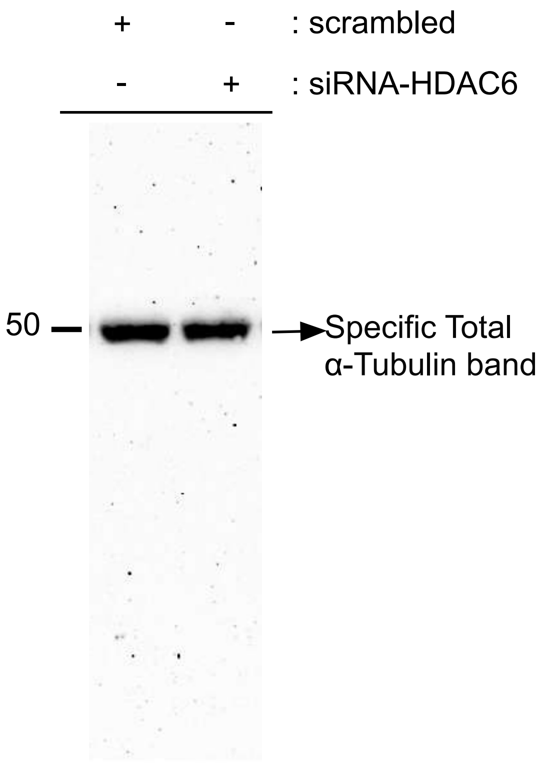

Figure S4D. *Replicate 3 Acetylated α-Tubulin complete gel*

Western-blot associated with Figure 4D
Pérez-Yanes, S., et al.

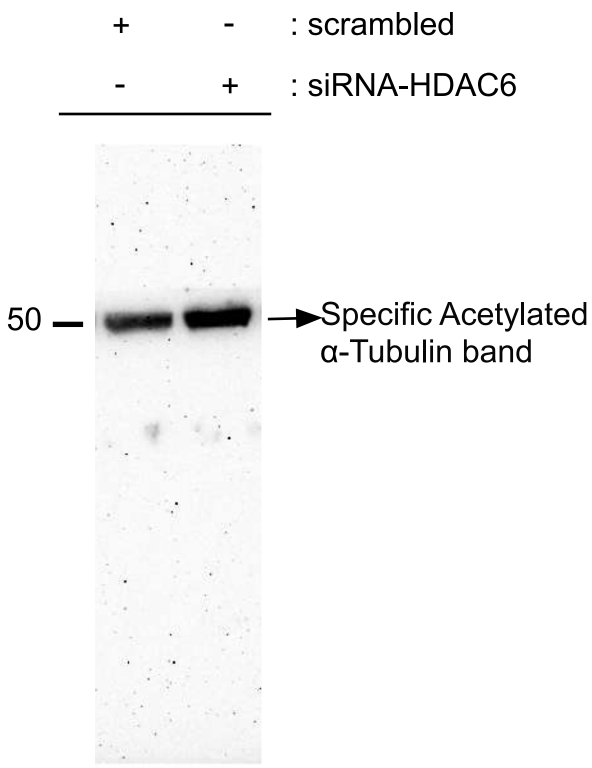

Figure S4D. *Replicate 4 HDAC6 complete gel*

Western-blot associated with Figure 4D
Pérez-Yanes, S., et al.

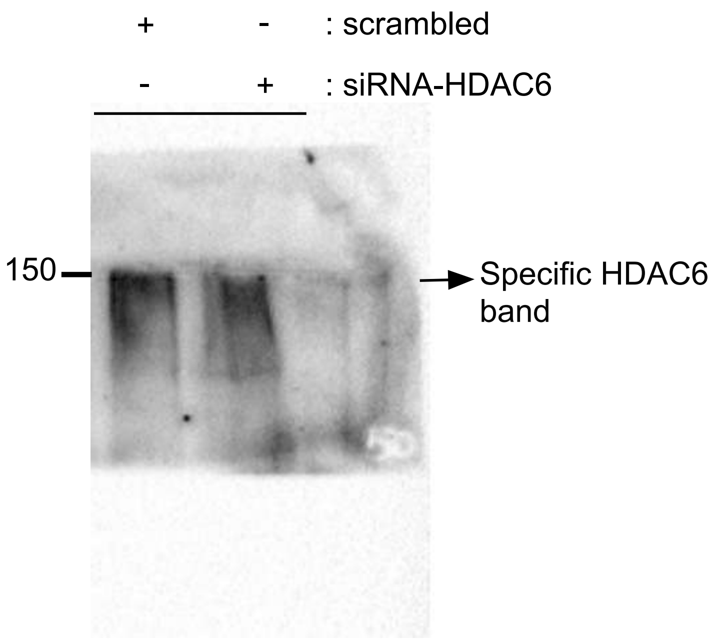

Figure S4D. *Replicate 4 myc complete gel*

Western-blot associated with Figure 4D
Pérez-Yanes, S., et al.

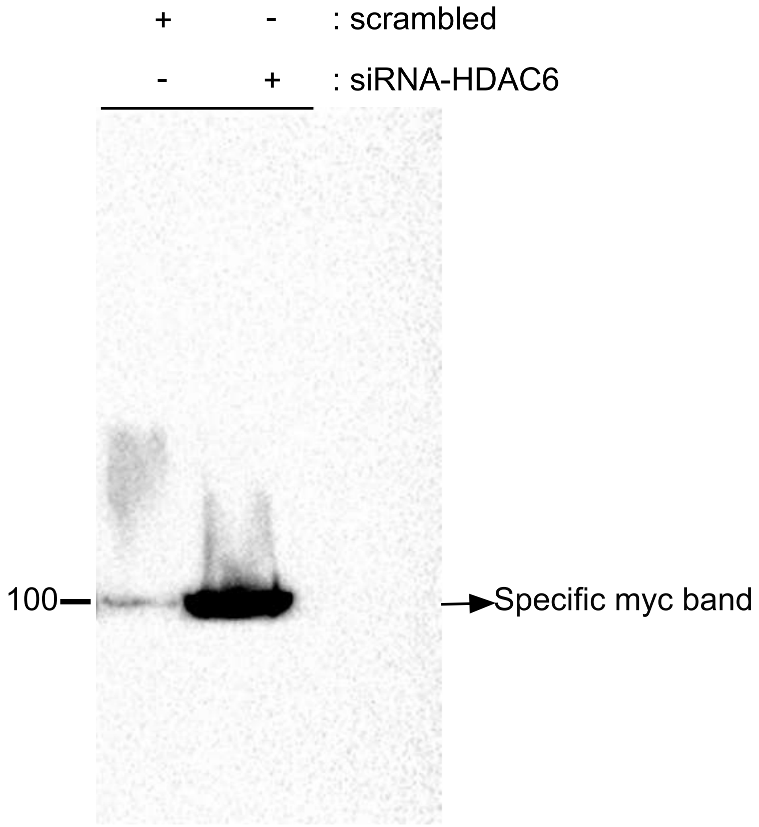

Figure S4D. *Replicate 4 Total α-Tubulin complete gel*

Western-blot associated with Figure 4D
Pérez-Yanes, S., et al.

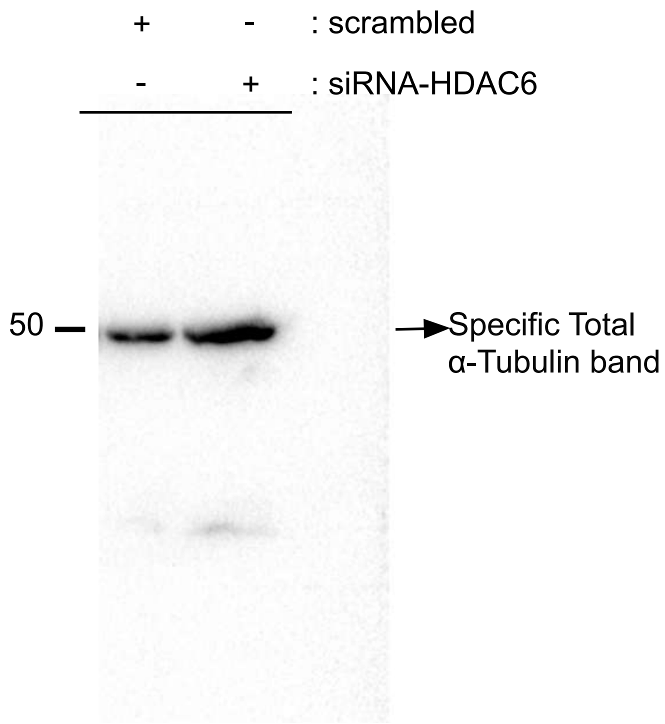

Figure S4D. *Replicate 4 Acetylated α-Tubulin complete gel*

Western-blot associated with Figure 4D
Pérez-Yanes, S., et al.

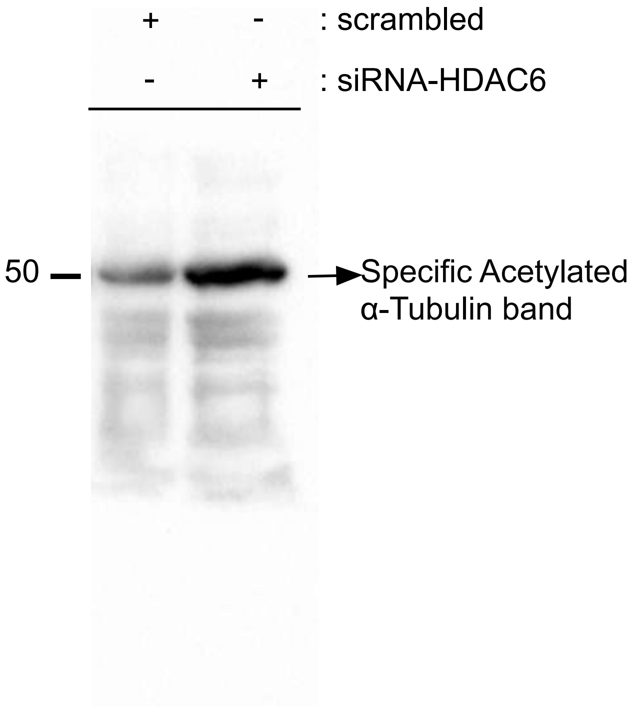

Figura S5B. Western-blot replicates associated with Figure 5B.

Replicate 1 as figure format

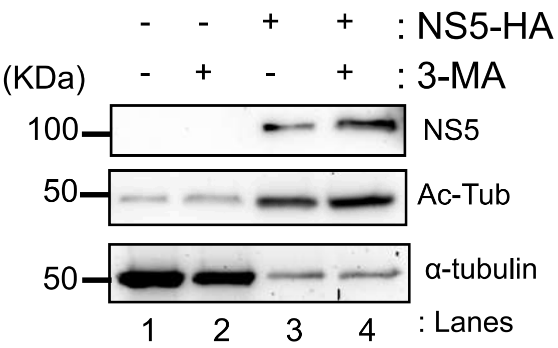

Replicate 2 as figure format

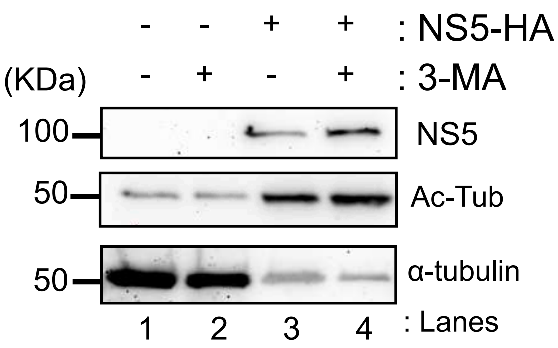

Figure S5B. *Replicate 1 NS5 complete gel*

Western-blot associated with Figure 5B
Pérez-Yanes, S., et al.

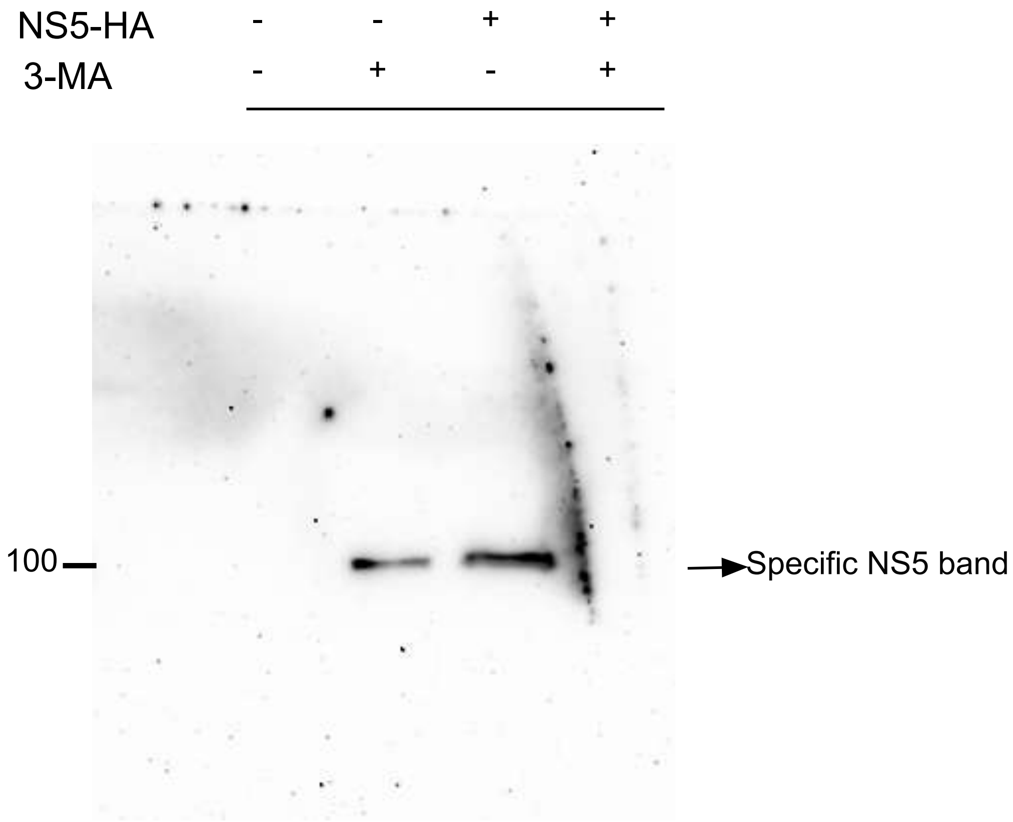

Figure S5B. *Replicate 1 Total α-Tubulin complete gel*

Western-blot associated with Figure 5B
Pérez-Yanes, S., et al.

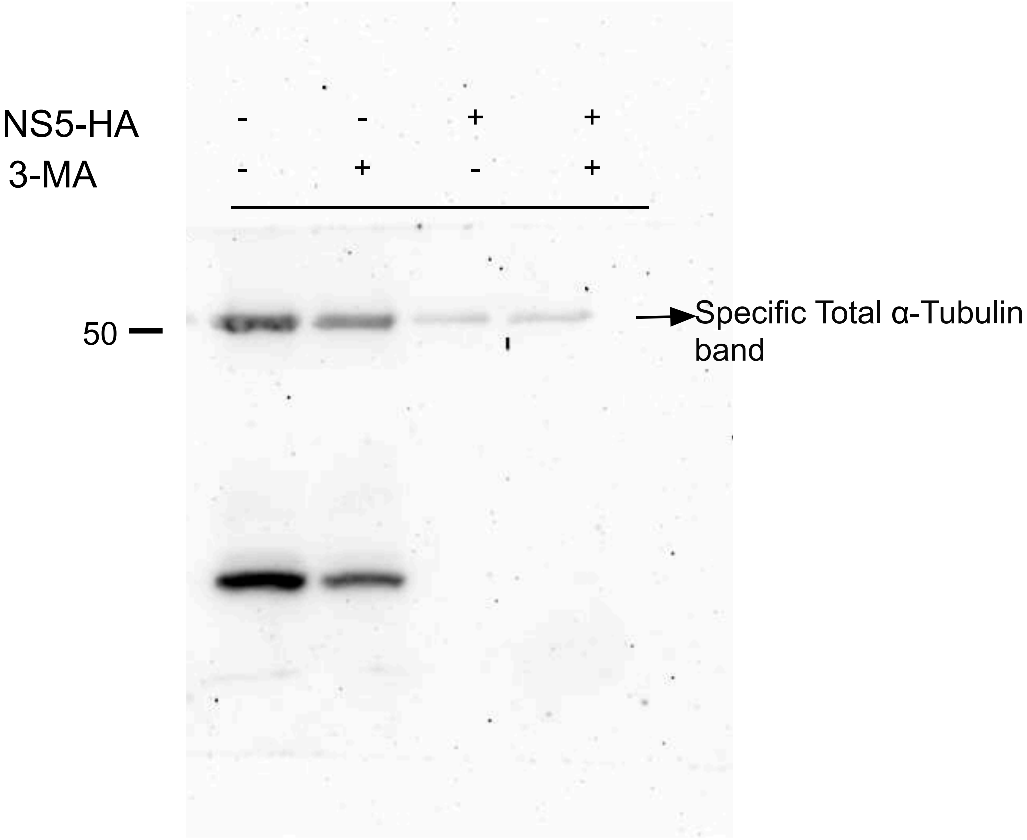

Figure S5B. *Replicate 1 Acetylated α-Tubulin complete gel*

Western-blot associated with Figure 5B
Pérez-Yanes, S., et al.

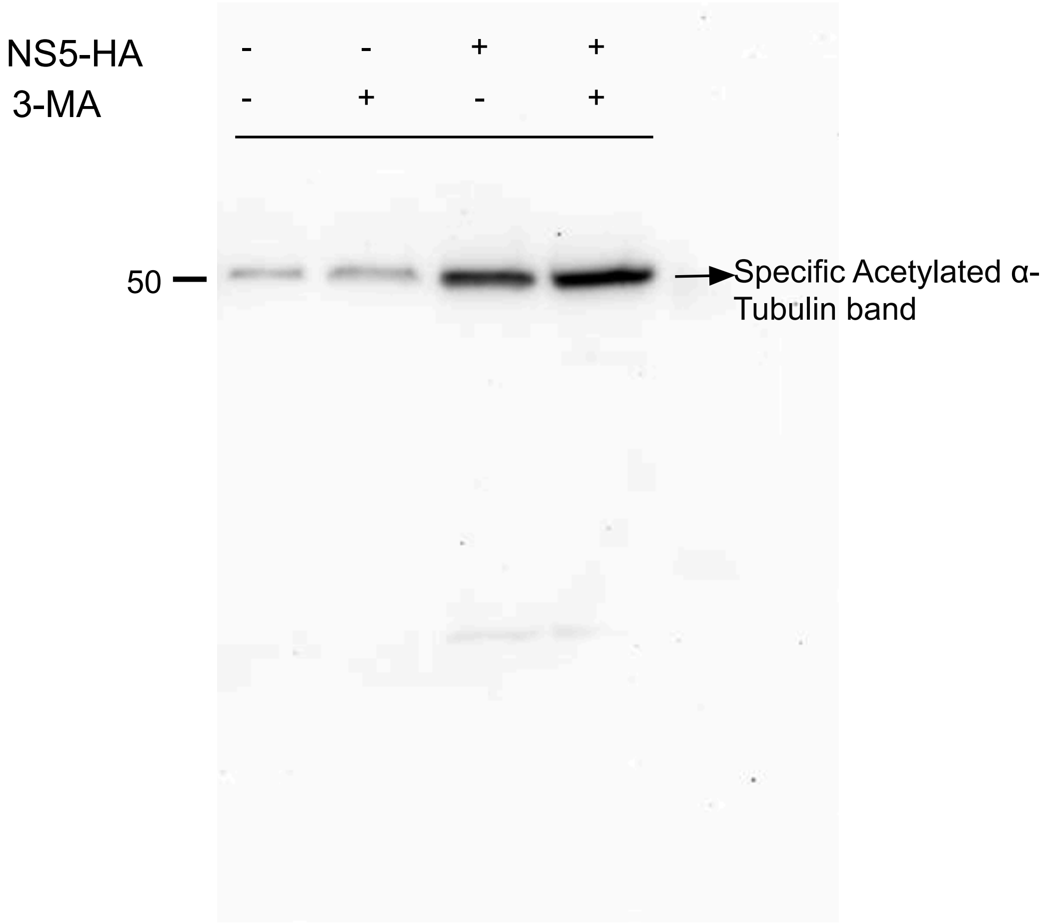

Figure S5B. *Replicate 2 NS5 complete gel*

Western-blot associated with Figure 5B
Pérez-Yanes, S., et al.

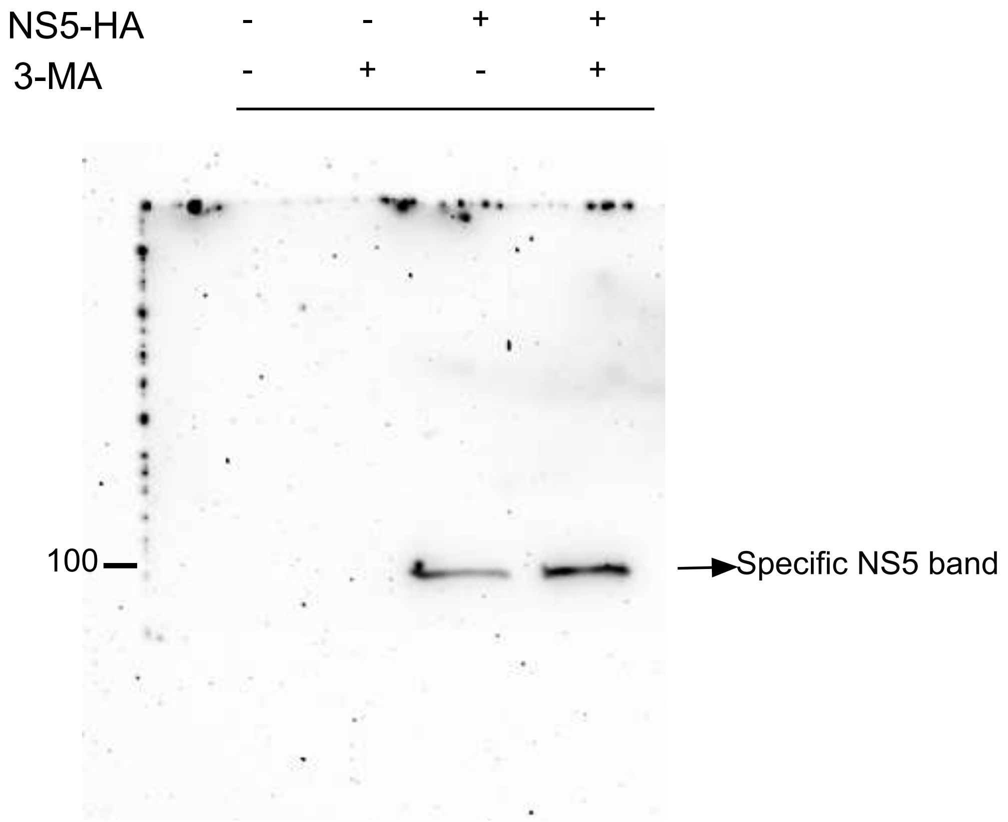

Figure S5B. *Replicate 2 Total α-Tubulin complete gel*

Western-blot associated with Figure 5B
Pérez-Yanes, S., et al.

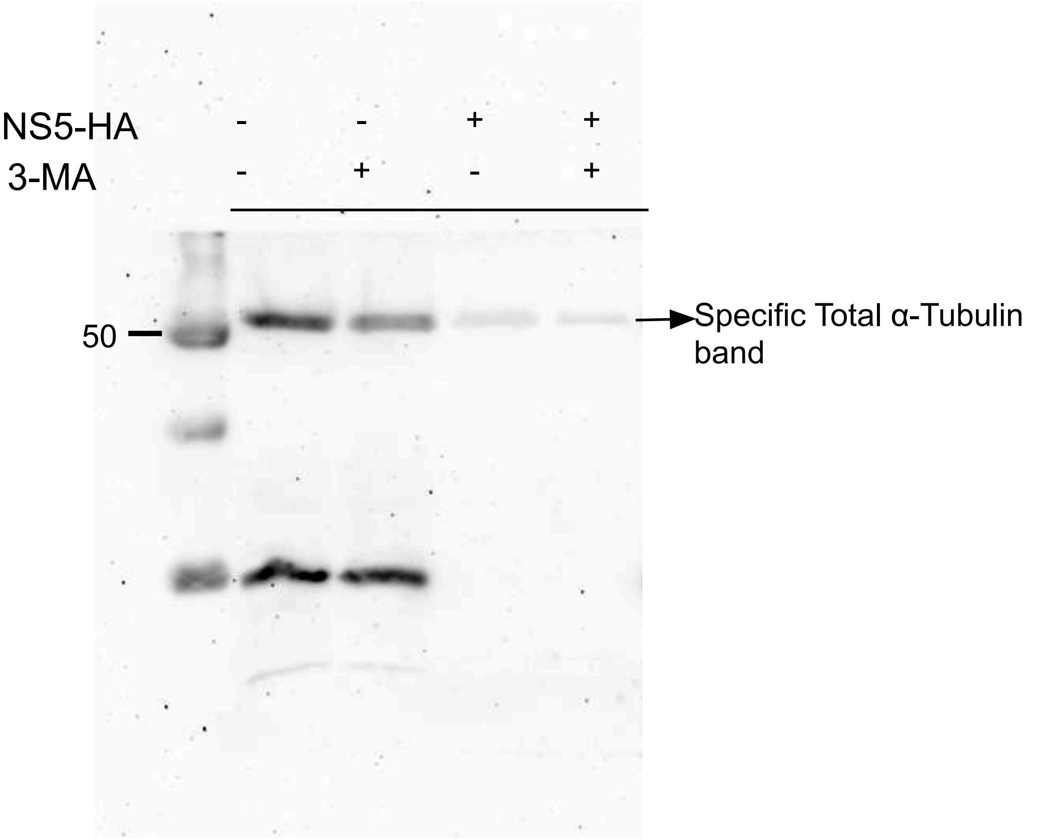

Figure S5B. *Replicate 2 Acetylated α-Tubulin complete gel*

Western-blot associated with Figure 5B
Pérez-Yanes, S., et al.

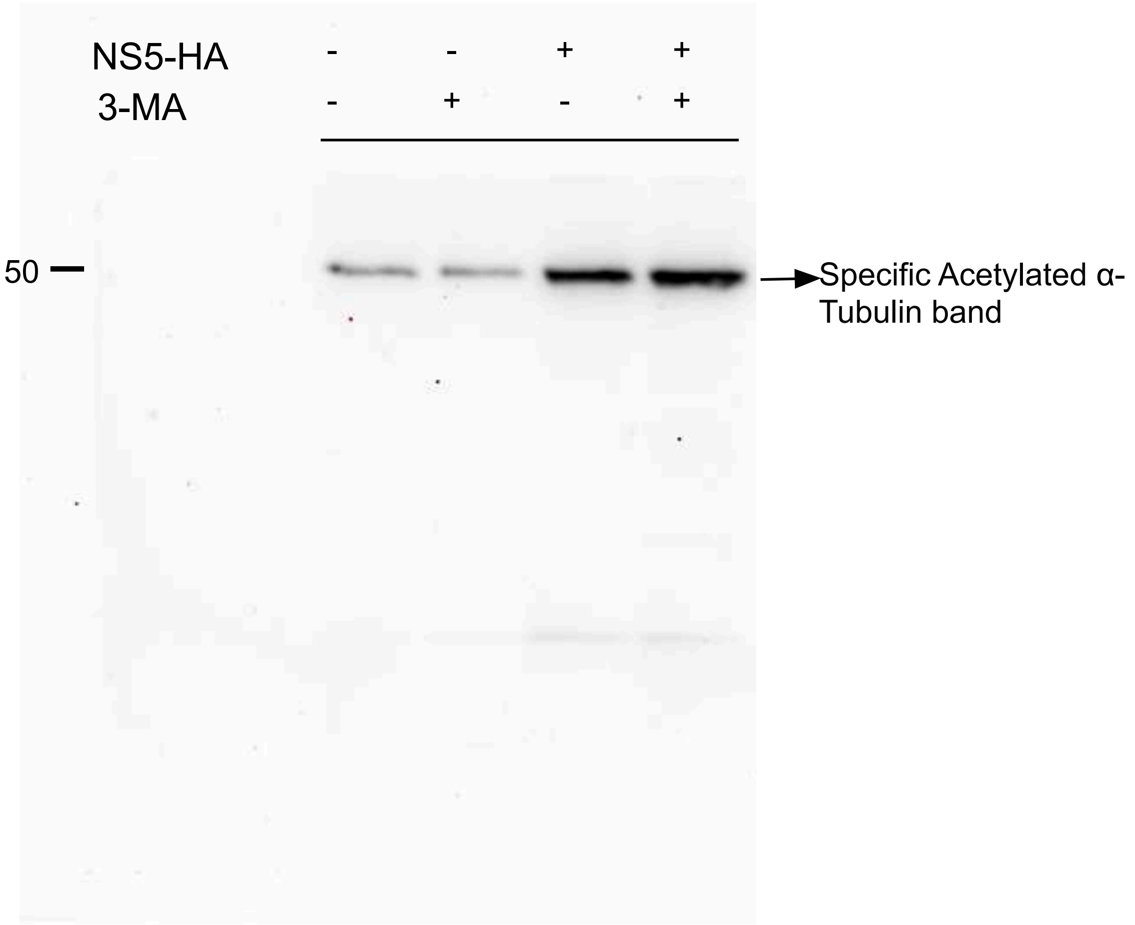

Supplement: Supplementary file 1 [file cells-13-00598-s001.zip › cells-2916729-supplementary.pdf]
